# Multidrug efflux pumps and innate azole resistance of *Mucor lusitanicus*

**DOI:** 10.1093/jac/dkaf343

**Published:** 2025-09-30

**Authors:** Stephanie Toepfer, Erwin Lamping, Jasper E James, Lisa-Maria Zenz, Julia Loacker-Schoech, Katharina Rosam, Olivia Majer, Michaela Lackner

**Affiliations:** Institute of Hygiene and Medical Microbiology, Medical University of Innsbruck, Innsbruck, Austria; Faculty of Dentistry, Sir John Walsh Research Institute, University of Otago, Dunedin, New Zealand; Institute of Hygiene and Medical Microbiology, Medical University of Innsbruck, Innsbruck, Austria; Institute of Hygiene and Medical Microbiology, Medical University of Innsbruck, Innsbruck, Austria; Institute of Hygiene and Medical Microbiology, Medical University of Innsbruck, Innsbruck, Austria; Institute of Hygiene and Medical Microbiology, Medical University of Innsbruck, Innsbruck, Austria; Max Planck Institute for Infection Biology, Berlin, Germany; Institute of Hygiene and Medical Microbiology, Medical University of Innsbruck, Innsbruck, Austria

## Abstract

**Objectives:**

This study aims to characterize the possible contribution of eight pleiotropic drug resistance (PDR) transporters to the azole resistance phenotype of *Mucor lusitanicus*.

**Methods:**

Gene expression analysis (RNA-sequencing and RT-qPCR) was performed on *M. lusitanicus* CBS277.49 cells exposed to three different types of azoles (4.0 mg/L). C-terminally GFP-tagged *M. lusitanicus* PDR transporters were overexpressed in the hypersensitive model host, *Saccharomyces cerevisiae* ADΔΔ. Their efflux pump functions were evaluated by determining the azole susceptibilities of the PDR transporter overexpressing cells and measuring their plasma membrane ATPase activities.

**Results:**

*M. lusitanicus* PDR transporters separated into two phylogenetic clusters: A (*pdr1*, *pdr6*-*8*) and B (*pdr2*-*5*). RNA-sequencing and RT-qPCR revealed strong up-regulation of *pdr1* and *pdr6*, but down-regulation of *pdr7* and *pdr8* in response to 80 min exposures of 4.0 mg/L voriconazole, isavuconazole or posaconazole. The expression of Pdr6 and Pdr7 in *S. cerevisiae* ADΔΔ increased its resistance to short- and mid-length tailed azoles. Pdr1 and Pdr8 expression, however, conferred pan-azole resistance including long-tailed azoles such as itraconazole and posaconazole. No efflux pump function and ATPase activity were detected for Pdr3 and Pdr5. The ATPase activities of Pdr1, Pdr6, Pdr7 and Pdr8 were comparable to *Candida albicans* Cdr1 expressed in ADΔΔ.

**Conclusions:**

All *Mucor* cluster A PDR transporters are multidrug efflux pumps, but Pdr1 and Pdr6 are possibly the major contributors to the innate azole resistance phenotype of *M. lusitanicus*.

## Introduction

Mucormycosis, a severe mould infection affecting mainly immunocompromised individuals, is of growing concern.^1^ Treatment options include surgical debridement^2^ and a limited choice of antifungals including amphotericin B or a select few azoles (mid- or long-tailed).^2,3^ Short-tailed triazoles, such as voriconazole, are not active against mucormycetes.^4,5^ Instead, salvage treatment with posaconazole or isavuconazole is recommended.^6–10^ Understanding the molecular mechanisms of antifungal resistance is essential for the development of more effective antifungals. A main azole resistance mechanism is the overexpression of drug efflux pumps. The *Saccharomyces cerevisiae* pleiotropic drug resistance (PDR) ATP-binding cassette (ABC) transporter Pdr5 and *Candida albicans* drug resistance 1 protein (Cdr1) are the best studied fungal multidrug efflux pumps.^11–14^ The overexpression of Pdr5/Cdr1 homologues such as *Candida glabrata* Cdr1,^15^  *Aspergillus fumigatus* Cdr1B,^16–19^  *Cryptococcus neoformans* Afr1^20–22^ and *Fusarium keratoplasticum* Abc1^23^ frequently causes azole resistance of clinically important fungal pathogens.

PDR transporters actively transport a wide variety of xenobiotics, including azoles, across cell membranes.^24^  *S. cerevisiae* Pdr5^13,25–27^ expression is regulated by the zinc cluster transcription factors Pdr1 and Pdr3^28–30^ and its overexpression due to gain of function mutations in *PDR1* can protect cells against hundreds of xenobiotics. The deletion of Pdr5, however, results in a drug-hypersensitive phenotype.^31–33^ Research of *Mucor* PDR transporters is limited to a publication by Nagy *et al.*,^34^ who studied the possible involvement of PDR transporters in *Mucor circinelloides* (*M. lusitanicus*) CBS277.49.

This study aimed to characterize *M. lusitanicus* PDR transporters in the heterologous host *S. cerevisiae* ADΔΔ^35^ and investigate their possible role in azole resistance. Heterologous overexpression in the drug-hypersensitive ADΔΔ host lacking seven major efflux pumps is a valuable tool to study the efflux pump function of unknown transporters because this strain is devoid of any other efflux pumps that could mask their efflux pump phenotype.^36–38^

## Materials and methods

A detailed description is provided in File S1 (available as Supplementary data at *JAC* Online).

### Culture conditions

*Mucor lusitanicus* CBS277.49 was grown on YPG at 28°C. For RNA extraction, YPG liquid medium was inoculated with 2 × 10^4^ cfu/mL. *S. cerevisiae* ADΔΔ^35^ was maintained on YPD. ADΔΔ uracil prototroph transformants were selected on CSM-URA plates incubated for 3 days at 28°C. A list of strains is provided in Table S1.

### PDR transporter inventories of *Mucor* species

Full-size PDR transporters were identified with a BLAST search of the *M. lusitanicus* CBS277.49 genome^39^ using *S. cerevisiae* S288C Snq2, Pdr5 and YOL075C as queries. Closely related homologues were identified in five other *Mucor* species: *M. circinelloides f. circinelloides* 1006PhL,^40^  *M. ambiguus* NBRC6742, *M. racemosus* UBOCC-A-109155, *M. endophyticus* UBOCC-A-113049 and *M. lanceolatus* UBOCC-A-109153^41^ (File S2).

### Phylogenetic analysis

Sequence alignments were performed with Clustal Omega^42^ and edited with Jalview2.11.4.0.^43^ Tree reconstructions were performed with Phylemon 2.0.^44^ FigTree v1.4.4 (http://tree.bio.ed.ac.uk/software/figtree/) was used for phylogenetic tree generation. Phylogenetic relationships were calculated by maximum likelihood analysis using PhyML v3.0.^45,46^ A list of all manually curated PDR transporter sequences used for phylogenetic tree construction is provided in File S2.

### Isolation of total RNA and RT-qPCR

*M. lusitanicus* CBS277.49 cells were grown to early log-phase and incubated for a further 80 min in the absence or presence of either 4.0 mg/L voriconazole, isavuconazole or posaconazole. Total RNA was extracted with the hot-phenol extraction method.^47^ RNA integrity was confirmed by formaldehyde agarose gel electrophoresis (Figure S1).

First strand cDNA was synthesized using the LunaScript RT SuperMix Kit (NEB, Germany). An average quantification cycle (Cq) value was calculated from technical duplicates. The mRNA transcript levels were normalized to the glyceraldehyde-3-phosphate dehydrogenase housekeeping gene, *gpd3*. The fold change values of mRNA expression levels were calculated using the ΔΔCq method.^48^ DNA oligonucleotide primers are listed in Table S2.

### Transcriptome analysis

RNA samples were sequenced at GENEWIZ Germany GmbH. Data were evaluated using FastQC (v.0.11.5) and sequence reads were trimmed using Trimmomatic (v.0.36). RNA-sequence data were deposited at the NCBI Short Read Archive (SRA) with BioProject accession number PRJNA1075823. Box plot analysis was performed to compare expression values (Figure S2). The similarity within and between each of the treatment groups *versus* the untreated control group was assessed by principal component analysis (Figure S3). A list of all normalized counts calculated from the raw data counts is provided in File S3.

### Plasmid pABC3XL

**Figure 1.**
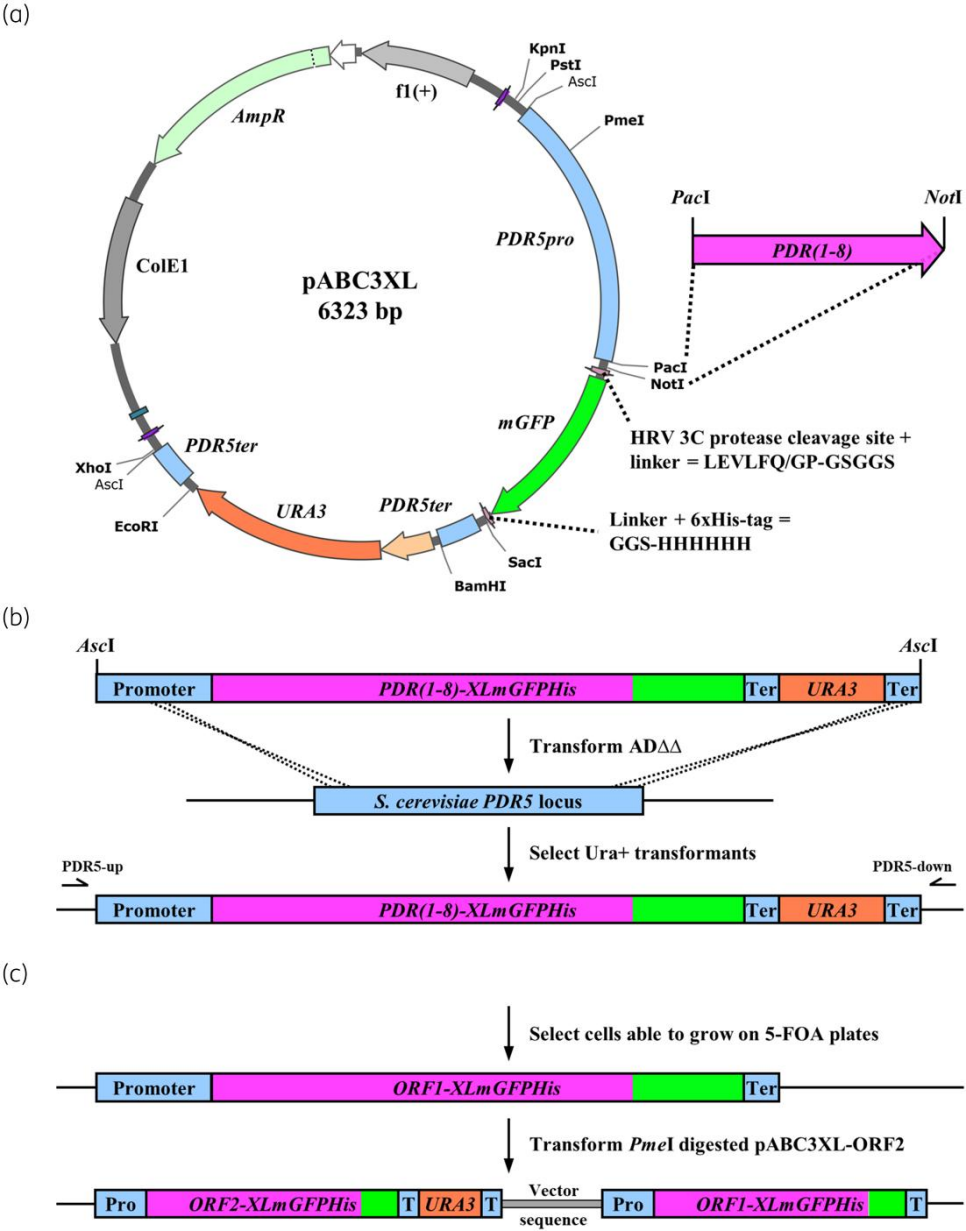
Plasmid pABC3XL and strategies to create ADΔΔ cells overexpressing one or two ORFs stably integrated into the genomic *PDR5* locus. (a) Map of the multifunctional pABC3XL plasmid, with a novel recyclable *URA3* blaster cassette flanked by two identical 196 bp *PDR5* terminator sequences (see Materials and methods for further details). (b) After cloning *M. lusitanicus* PDR transporters *pdr1*-*8* as *Pac*I/*Not*I fragments into pABCXL, the ∼8 kb transformation cassettes containing the *M. lusitanicus* ORFs were excised with *Asc*I, gel-purified and used to transform *S. cerevisiae* ADΔΔ cells. Integration of the entire transformation cassette into the genomic *PDR5* locus occurred via two homologous recombination events (dashed crosses) at 100% efficiency. Correct uracil positive transformants were confirmed by PCR amplification of the gDNA region using DNA oligomer primer pair PDR5-up/PDR5-down and sequencing the entire ORF including ∼100 bp of the flanking regions. Correct uracil prototrophic transformants were used to characterize the expression, the localization and the efflux pump function of the six *M. lusitanicus* PDR transporters. (c) Strategy for creating tandem gene arrays stably integrated into the genomic *PDR5* locus. After removal of the *URA3* marker from ADΔΔ-ORF1-XLmGFPHis cells created in (b) by selecting colonies of cells that are able to grow on 5-FOA plates (top line), the resulting strain (top line) is transformed with the *Pme*I-digested linear pABC3XL-ORF2-XLmGFPHis plasmid to create a uracil prototrophic transformant that has a tandem gene array of ORF2 and ORF1 stably integrated into the genomic *PDR5* locus (bottom line), both ORFs expressed at equal amounts because each ORF is driven by one of the two *PDR5* promoter repeats (see Materials and methods for further details).

The creation of the novel pABC3XL (Figure 1a) and the pABC3 blaster derivatives is described in File S1. Key features of the pABC3XL plasmid are as follows: (i) unique cloning sites between each of the individual modules of the *Asc*I transformation cassette (Figure 1b), (ii) a unique 8 bp *Pme*I restriction site in the *PDR5* promoter and (iii) a ‘true’ *URA3* blaster cassette (Figure 1a). The *URA3*-blaster cassette together with the unique 8 bp *Pme*I restriction site of pABC3XL enables the overexpression of two proteins of interest driven by the same constitutively active *PDR5* promoter stably integrated as tandem-arrays into the genomic *PDR5* locus (Figure 1c).

### Heterologous expression of PDR transporters

Six PDR transporters (*pdr1*, *pdr3*, *pdr5*, *pdr6*, *pdr7* and *pdr8*) were amplified from a cDNA template using oligonucleotide primers (Table S2) containing either *Pac*I or *Not*I restriction sites at their respective 5′ ends. The Q5 High-Fidelity DNA Polymerase (NEB) was used for PCR amplification. Gel-purified *Pac*I/*Not*I digested PCR products were used for ligation into the *Pac*I/*Not*I digested pABC3XL plasmid and transformed into *Escherichia coli* DH5α. Plasmids created in this study (Table 1) were confirmed by DNA sequencing. Correct integration at the genomic *PDR5* locus was confirmed by colony PCR using the KOD One™ PCR Mastermix Blue (TOYOBO Co., Ltd., Japan).

**Table 1.** Plasmids used or pABC3 derivative plasmids described for the first time in this study

Plasmid	Description	GenBank accession #	Reference
pABC3-blaster^a^	pABC3 derivative	PQ407573	This study
pABC3-GFP3-blaster^a^	pABC3-GFP derivative	PQ407574	This study
pABC3-mRFP1-blaster^a^	pABC3-mRFP derivative	PQ407575	This study
pABC3-XmGH	Former pABC3-XLmGFPHis plasmid	PQ407576	^23^
pABC3XL	*Pme*I, XLmGFPHis, *URA3*-blaster	PQ407577	This study
pABC3-Mlus-pdr1-XL	pABC3XL containing *pdr1*	PQ407578	This study
pABC3-Mlus-pdr3-XL	pABC3XL containing *pdr3*	PQ407579	This study
pABC3-Mlus-pdr5-XL	pABC3XL containing *pdr5*	PQ407580	This study
pABC3-Mlus-pdr6-XL	pABC3XL containing *pdr6*	PQ407581	This study
pABC3-Mlus-pdr7-XL	pABC3XL containing *pdr7*	PQ407582	This study
pABC3-Mlus-pdr8-XL	pABC3XL containing *pdr8*	PQ407583	This study

^a^These plasmids do not contain true *URA3* blaster cassettes (see Materials and methods for further details). They only create recyclable *URA3* ‘blaster’ cassettes after integration of the *Asc*I transformation cassettes into the genomic *PDR5* locus.

### Susceptibility testing

Antifungal susceptibility testing was performed according to the EUCAST protocol (definition 7.3.2)^49^ but with minor modifications, because *S. cerevisiae* ADΔΔ does not grow in RPMI 1640. Antifungals tested were anidulafungin, itraconazole, posaconazole, fluconazole (Sigma-Aldrich), oteseconazole (MicroCombiChem), isavuconazole and voriconazole (Pfizer, Inc.).

### Plasma membrane isolation and protein quantification

Crude plasma membranes were isolated from 5 mL YPD overnight cultures. The protein concentration was measured with the Lowry Method (DC^™^ Protein Assay Kit II, Bio-Rad, Germany). Ten micrograms crude plasma membrane proteins were separated by SDS-PAGE (8% polyacrylamide gel) as previously described.^50^ Fluorescent signals were measured with ChemiDoc Imaging System (Bio-Rad) and analysed with Image Lab Software.

### ATPase activities

ATPase activities of *M. lusitanicus* PDR transporters were determined with crude plasma membranes isolated as described by Madani *et al.*^51^ The absorbance was measured with a Varioskan LUX Multimode Microplate Reader (Thermo Fisher, USA) at 750 nm.

### Structured illumination microscopy

The visualization of fluorescence signals of *S. cerevisiae* strains overexpressing C-terminally GFP-tagged *M. lusitanicus* PDR transporters was performed using the Structured Illumination Microscope (SIM) Elyra 7 (Carl Zeiss GmbH, Germany) with a Plan-Apochromat 63×/1.4 Oil DIC M27 objective, Lens 1.6×. Cell images were processed with the associated ZEISS ZEN core software. Maximal resolution of images was calculated according to Gustafsson.^52^

### Visualization of figures

The volcano plots and Venn diagrams were generated on R version 4.2.0,^53^ using DESeq2 version 1.16.1^54^ and ggplot2 package version 3.3.6,^55^ and VennDiagram package version 1.7.3,^56^ respectively. Heatmaps were created using GraphPad Prism 10 (GraphPad Software, LLC).

## Results

### Phylogeny of PDR transporters

**Figure 2.**
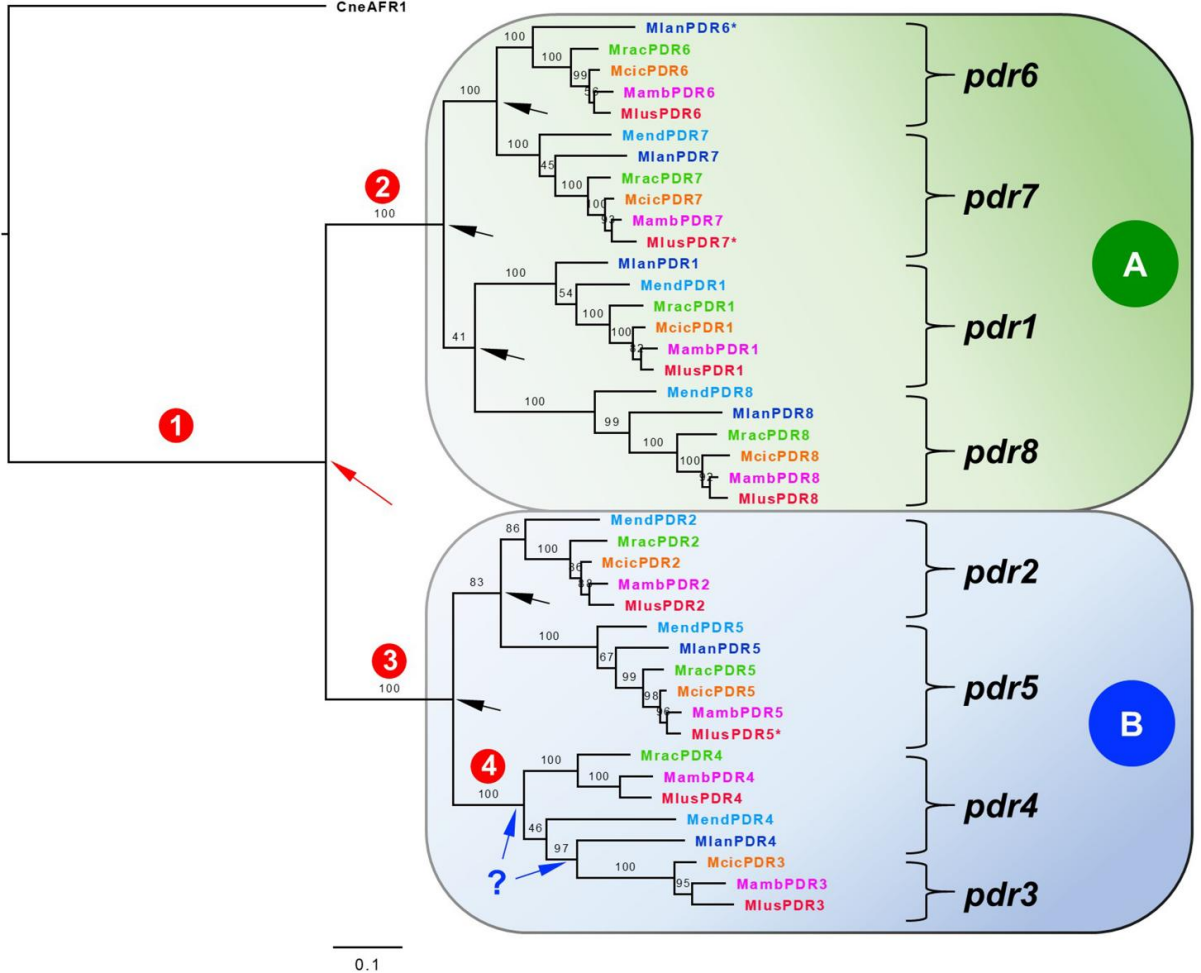
Phylogenetic tree of all full-size *Mucor* PDR transporters of six representative *Mucor* species. *Mucor* PDR transporters are fungal cluster H3 PDR transporters (see Figure 3) with one common ancestor (encircled number 1). In the distant past this ancestor most likely evolved by tandem gene duplication (long arrow) into the common ancestors (2) and (3) of the two major clusters of *Mucor* PDR transporters. Cluster A comprises orthologs of *pdr6*, *pdr7*, *pdr1* and *pdr8*, and cluster B comprises orthologs of *pdr2*, *pdr5*, *pdr4* and *pdr3*. The tandem duplicated *pdr6/pdr2* genes of *M. lusitanicus*, *M. ambiguus* and *M. circinelloides f. circinelloides* are quite possibly remnants of the original tandem gene duplication event mentioned above. Interestingly, these two orthologs have lost their physical connection in the *M. racemosus* genome, quite likely through chromosomal rearrangement in the more recent past. The other two *Mucor* species *M. endophyticus* and *M. lanceolatus* each lost one of these two orthologs (i.e. *pdr6* and *pdr2*, respectively) in the more recent past. At least six additional gene duplications (short arrows) led to the evolution of the remaining six orthologs *pdr1*, *pdr3*, *pdr4*, *pdr5*, *pdr7* and *pdr8*, all of which, apart from the duplication (short arrow with question mark; it is not clear when the duplication actually happened) of the common ancestor (4) of *pdr3* and *pdr4*, happened before the speciation of the six investigated *Mucor* species. The scale bar indicates the calculated substitutions per site. Species abbreviations: Mlus, *M. lusitanicus*; Mamb, *M. ambiguus*; Mend, *M. endophyticus*; Mlan, *M. lanceolatus*; Mcic, *M. circinelloides f. circinelloides*; Mrac, *Mucor racemosus*.

**Figure 3.**
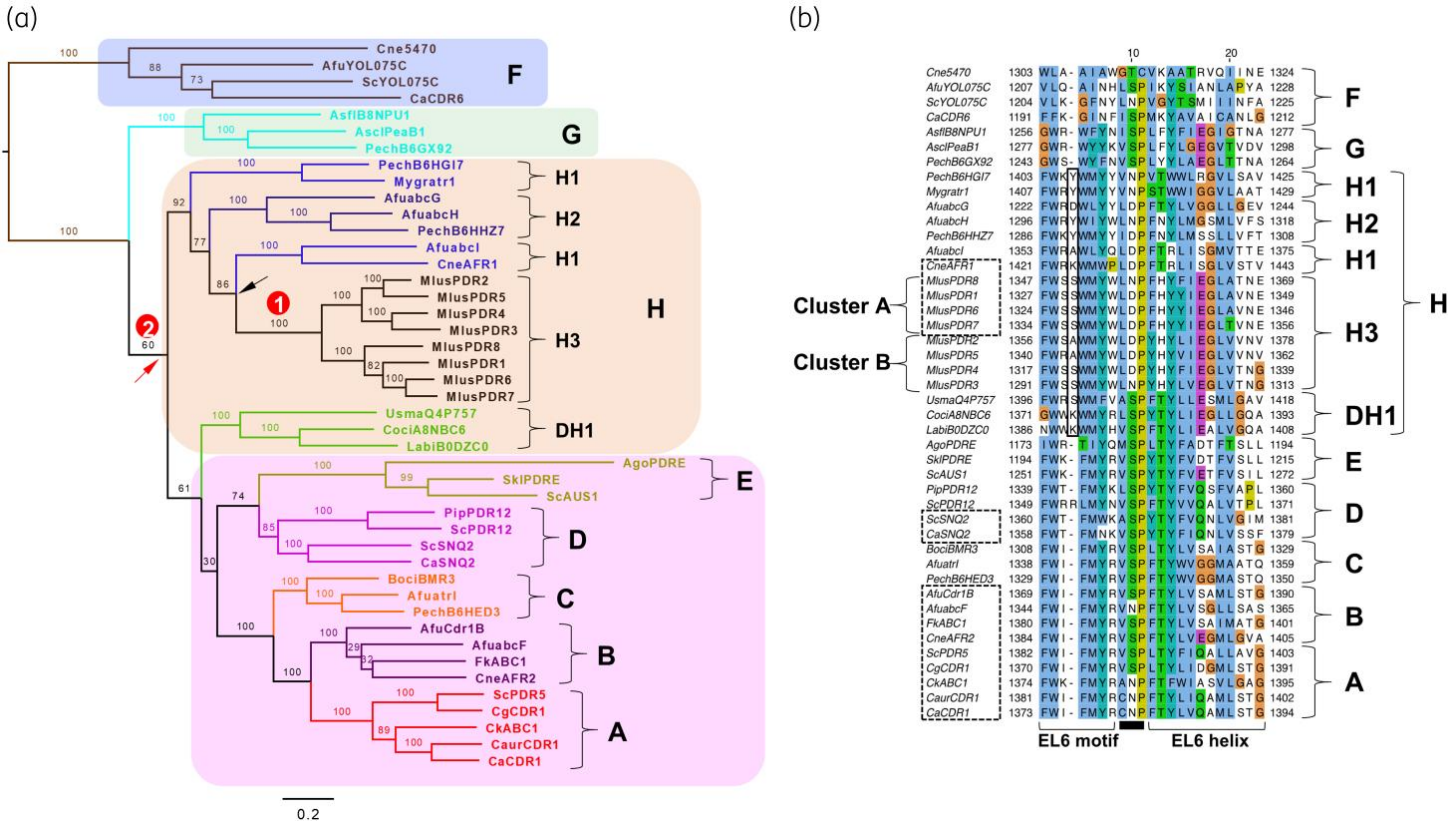
Phylogenetic tree and alignment of EL6 motif residues of all full-size fungal PDR transporters. (a) Maximum likelihood tree providing a tentative model for the evolution of all full-size fungal PDR transporters, including *M. lusitanicus pdr1*-*pdr8*, following the classification of Lamping *et al.*^57^  *Mucor* PDR transporters clearly have one common ancestor (encircled number 1; 100% bootstrap support), possibly derived from a gene duplication event (arrow) of a cluster H1 PDR transporter ancestor. Cluster F PDR transporters are the common ancestor of all full-size fungal PDR transporters. Interestingly, cluster DH1 PDR transporters also have the cluster H hallmark insertion of one extra residue in the PDR transporter defining EL6 motif (b). It is therefore highly likely that cluster DH1 PDR transporters are another sub-cluster of cluster H PDR transporters sharing the common ancestor (encircled number 2) with all cluster H1, H2 and H3 PDR transporters. This observation is possibly the major reason of the weak bootstrap support for the three major branches (i.e. 60%, 61% and 30%) leading from cluster G to cluster E-D PDR transporters. In other words, the DH1 branch should attach near the top of the cluster H branch that has a 92% bootstrap support and the branch leading to all cluster E–A PDR transporters should attach directly to the cluster G branch, because no other PDR transporters have the cluster H hallmark insertion in the EL6 motif. (b) The conserved EL6 motif, proline kink (black line) and EL6 helix^57^ form an elbow helix that embraces the transmembrane domain of the C-terminal half of PDR transporters at the outer water-lipid bilayer interface. This is based on recent structures published for *S. cerevisiae* Pdr5.^58^ This C-terminal and the equivalent N-terminal elbow helix comprising the PDR transporter defining PDRA and PDRB motives^57^ provide two flexible hinge regions surrounding the substrate exit gate^71^ that ensure proper opening and closing of the transporter during substrate translocation. All cluster H PDR transporters have a one amino acid insertion (black rectangle) in the EL6 motif. The numbers to the left and right of the sequences indicate the first and last amino acid position in the respective PDR transporter. Multidrug efflux pumps that are known or likely to be involved in drug resistance of various fungal pathogens and *S. cerevisiae* are highlighted with a dashed rectangle.

*M. lusitanicus* had eight full-size PDR transporters, named *pdr1* to *pdr8* according to the nomenclature introduced by Nagy *et al.*^34^ A phylogenetic tree of manually curated *M. lusitanicus* PDR transporters (*n* = 8) and all PDR transporters of five additional *Mucor* species confirmed two distinct *Mucor* PDR transporter clusters (Figure 2), as reported by Nagy *et al.*^34^ Cluster A included *pdr1*, *pdr6*, *pdr7* and *pdr8* orthologs and cluster B contained the remaining four orthologs *pdr2*, *pdr3*, *pdr4* and *pdr5*. The percent identity between orthologs within each cluster varied from 73.3% to 97.1% (File S4). Interestingly, three cluster A (*pdr1*, *pdr7*, *pdr8*), but only one cluster B (*pdr5*) orthologs were conserved in all *Mucor* species (Figure 2). Further analysis revealed that all *Mucor* PDR transporters belong to fungal cluster H PDR transporters (Figure 3a) with a characteristic one amino acid insertion (Figure 3b) in the conserved EL6 motif (FWX_2_WhYX_3_P).^57^ They do, however, form a new, distinct, sub-cluster (H3). A description of the possible evolutionary history of *Mucor* PDR transporters is provided in Figures 2 and 3.

### Response of PDR transporter transcripts to azole exposure

**Figure 4.**
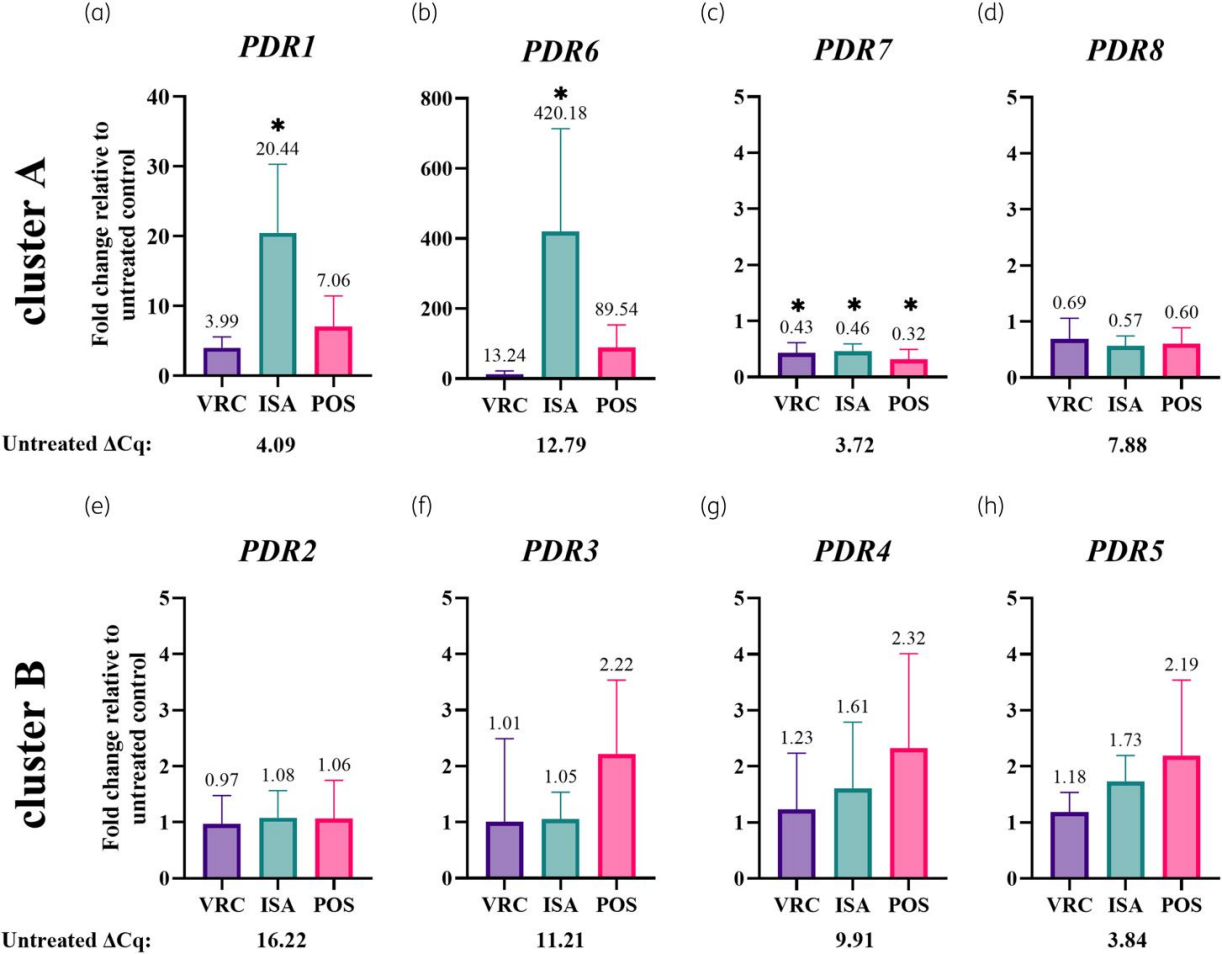
mRNA expression levels of *M. lusitanicus* PDR transporters exposed to three different azoles. The mRNA expression levels of *M. lusitanicus* PDR transporters in response to azole exposure were determined by RT-qPCR. Logarithmic *M. lusitanicus* cells were exposed to 4.0 mg/L VRC, ISA and POS for 80 min at 30°C. The X-fold changes of the mRNA levels relative to untreated control (UT, DMSO only) cells were calculated for the *gpd3* normalized mRNA expression levels. Statistical analysis was performed with GraphPad Prism 10 (GraphPad Software, LLC) using a one-way ANOVA test to compare with the untreated control. Bars represent the standard deviations of three biological replicates, and the asterisks indicate significant difference to the null hypothesis (*P* < 0.05). Numbers above the bar indicate the averages of three biological triplicates. The *gpd3* normalized mRNA expression levels (ΔCq values) in the untreated control cells are shown underneath each graph. VRC, voriconazole; ISA, isavuconazole; POS, posaconazole.

**Figure 5.**
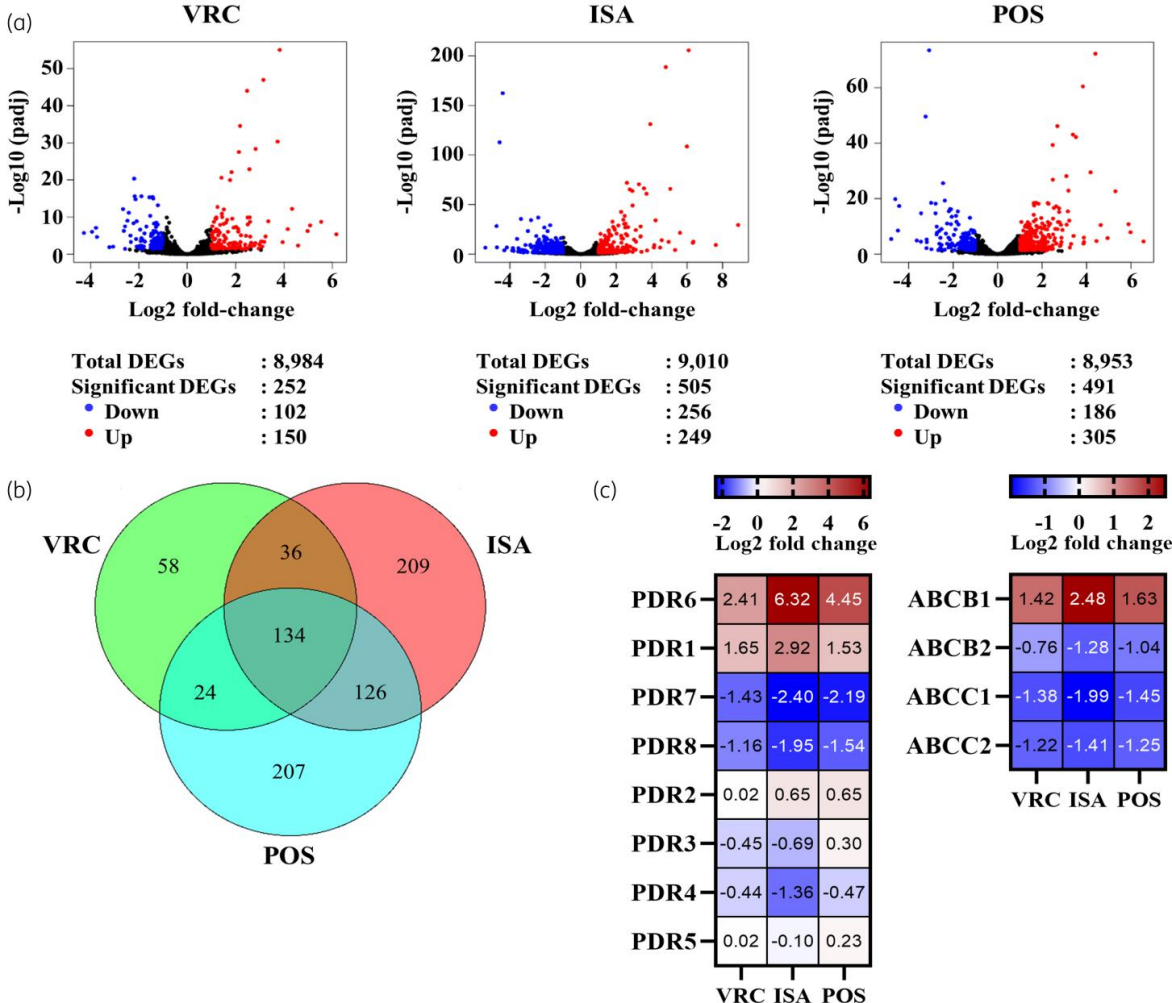
Analysis of DEGs. (a) Volcano plots of DEGs in *M. lusitanicus* CBS277.49 log-phase cells exposed to 4.0 mg/L VRC, ISA and POS for 80 min relative to log-phase cells grown for a further 80 min in the absence of azoles. Cut-off values of adjusted *P* < 0.05 and log2-fold changes > |1| were applied to determine significant DEGs. (b) A three-way Venn diagram depicting the relationship between the significant DEGs of *M. lusitanicus* cells exposed to 4.0 mg/L VRC, ISA and POS for 80 min. (c) Log2 fold changes (averages of three independent experiments) of mRNA expression levels determined by RNA-sequencing for all eight *M. lusitanicus* PDR transporters including four other ABC transporters that were among the list of significant DEGs. The heatmap was created using GraphPad Prism 10. VRC, voriconazole; ISA, isavuconazole; POS, posaconazole.

Expression levels were measured by two-step RT-qPCR (Figure 4) and by RNA-sequencing (Figure 5) the entire transcriptome of untreated cells and cells exposed to azoles. There was an excellent correlation (Pearson *r* = 0.996) between the fold up- and down-regulation of the mRNA expression levels determined by RT-qPCR or by RNA-sequencing (Figure S4).

The normalized expression levels (i.e. ΔCq values) of untreated cells revealed highest mRNA expression levels for *pdr7* followed by *pdr5*, *pdr1* and *pdr8*, respectively. Their ΔCq values were 3.7, 3.8, 4.1 and 7.9, respectively (Figure 4). These rather high expression levels are consistent with the observation that they are also the only four orthologs found in all six *Mucor* species investigated. In comparison, *pdr4*, *pdr3*, *pdr6* and *pdr2* had rather low expression levels with ΔCq values of 9.9, 11.2, 12.8 and 16.2, respectively (Figure 4).

Azole exposure strongly up-regulated the mRNA expression levels for *pdr1* (4-, 20- and 7-fold) and *pdr6* (13-, 420- and 90-fold; Figure 4a and b). The mRNA expression levels of *pdr3*, *pdr4* and *pdr5* did not respond to azole exposure apart from a slight increase (∼2-fold) in response to posaconazole (Figure 4f–h). The *pdr7* and *pdr8* mRNA expression levels were, however, significantly (>2-fold, Figure 4c) or slightly (∼1.6-fold, Figure 4d) reduced in response to the three azoles. The *pdr2* mRNA expression levels (Figure 4e) did not change in response to any of the three azoles.

The number of differentially expressed genes (DEGs) was similar between the three azole treatment groups. Among the ∼9000 DEGs 252 (voriconazole), 505 (isavuconazole) and 491 (posaconazole) were recognized as significantly differentially expressed (Figure 5a). Cells exposed to voriconazole had 150 up- and 102 down-regulated genes, to isavuconazole 249 up- and 256 down-regulated genes and to posaconazole 305 up- and 186 down-regulated genes. Among the significantly DEGs, 134 were common between the three treatment groups (Figure 5b) and 58, 209 and 207 DEGs appeared unique to voriconazole, isavuconazole or posaconazole treated cells, respectively. There were eight ABC and 23 MFS transporters among the DEGs (Table 2; File S5 and Figure S5). Apart from *pdr1* and *pdr6*, the RNA-sequencing results revealed one further ABC transporter, *abcb1*, an ABCB-type ABC transporter, as significantly up-regulated in response to all three azoles (Figure 5c). Consistent with the RT-qPCR results, the RNA-sequencing results also identified *pdr7* and *pdr8* as significantly down-regulated genes in response to all three azoles. One additional ABCB-type (*abcb2*) and two ABCC-type (*abcc1*, *abcc2*) ABC transporters were also down-regulated in response to all azoles (Figure 5c and Table 2). Eight of the 23 DEGs encoding MFS transporters were up-regulated (*mfs1*–*8*), the rest was down-regulated in response to two (*mfs17*) or all three azoles tested (*mfs9–16, mfs18–23*) (Figure S5). Although *mfs1* was the highest up-regulated MFS transporter in response to isavuconazole (21-fold), its expression level (1.62 TPM; transcripts per million) was still rather low (∼170-fold lower than *pdr1* (280 TPM)).

**Table 2.** Normalized expression levels of all *M. lusitanicus* PDR transporters including all ABC transporters that were significantly (log2 = > ± 1) up- or down-regulated in response to 4 mg/L VRC, ISA or POS

ABC transporters	Gene	log2 fold change of expression			TPM (averages of three independent experiments)			
		VRC	ISA	POS	UT	VRC	ISA	POS
Cluster A PDR	*pdr1*	1.7	2.9	1.5	31	107	280	97
	*pdr6*	2.4	6.3	4.5	0.7	4	66	16
	*pdr7*	−1.4	−2.4	−2.2	69	28	15	16
	*pdr8*	−1.2	−2.0	−1.5	4	2	1	2
Cluster B PDR	*pdr2*	0.02	0.7	0.7	0.1	0.1	0.1	0.1
	*pdr3*	−0.5	−0.7	0.3	0.8	0.6	0.5	1
	*pdr4*	−0.4	−1.4	−0.5	2	1	1	1
	*pdr5*	0.02	−0.1	0.2	112	114	104	131
ABCB	*abcb1*	1.4	2.5	1.6	23	67	152	77
	*abcb2*	−0.8	−1.3	−1.0	6	4	3	3
ABCC	*abcc1*	−1.4	−2.0	−1.5	2	1	1	1
	*abcc2*	−1.2	−1.4	−1.3	76	36	34	34
	*gpd3* ^a^	—	—	—	687	661	378	483

TPM, transcripts per million; VRC, voriconazole; ISA, isavuconazole; POS, posaconazole; UT, untreated control cells.

^a^The normalized expression levels of the *GAPDH* housekeeping gene *gpd3* were included for a comparison of the absolute expression levels expressed as TPM.

### *Mucor* PDR transporters expressed well in ADΔΔ

**Figure 6.**
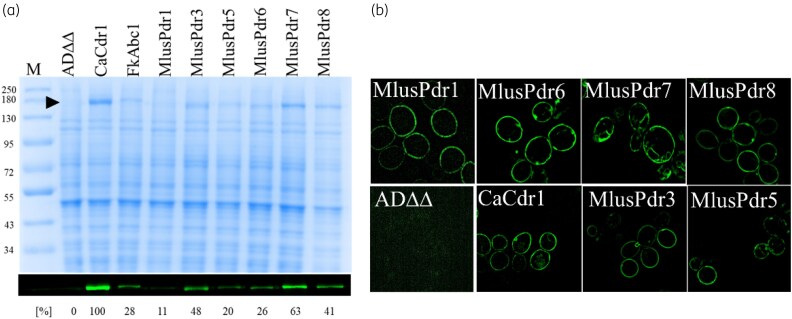
Protein expression levels and localization of full-size *M. lusitanicus* PDR transporters overexpressed in *S. cerevisiae* ADΔΔ. (a) SDS-PAGE of plasma membrane preparations (10 µg protein) isolated from *S. cerevisiae* ADΔΔ (negative control) cells overexpressing *C. albicans* Cdr1 (CaCdr1), *F*. *keratoplasticum* Abc1 (FkAbc1) and the six *M. lusitanicus* PDR transporters *pdr1*, *pdr3*, *pdr5*, *pdr6*, *pdr7* and *pdr8*; M is the Color Prestained Protein Standard (New England Biolabs GmbH) with the indicated molecular weights (kDa). The protein bands were stained with Coomassie blue R250 (Bio-Rad). The image underneath shows the green fluorescence signals of the proteins tagged with a C-terminal mGFPHis double tag that were determined before the proteins were stained with Coomassie blue. Triangle points towards the protein of interest. The protein expression levels are presented as percentages relative to CaCdr1 and are listed below the image. (b) Fluorescence microscopy of ADΔΔ cells overexpressing *M. lusitanicus pdr1*, *pdr3*, *pdr5*, *pdr6*, *pdr7*, *pdr8* and *C. albicans CDR1* each with a C-terminal mGFPHis double tag. Green fluorescent protein signals were detected with the Structured Illumination Microscope (SIM) Zeiss Elyra 7; Plan-Apochromat 63x/1.4 Oil DIC M27 objective, Lens 1.6×. Images were processed with the accompanying ZEN software. An image of cells of the host strain ADΔΔ is included as a negative control. SIM images were used for accurate protein localization studies, but they were not used for the quantification of the protein expression levels. The GFP signal intensities between the different SIM images are not comparable because the fluorescence signals had to be adjusted for some PDR transporters to achieve optimal visualization.

A new plasmid, pABC3XL, was used to overexpress *pdr1*–*pdr8* from the genomic *PDR5* locus of the hypersusceptible *S. cerevisiae* ADΔΔ host strain (Figure 1). The pABC3XL is an improved pABC3-XLmGFPHis^23,51^ derivative plasmid (File S1). Out of the eight transporters, the cDNA ORFs of six (*pdr1*, *pdr3*, *pdr5*, *pdr6*, *pdr7* and *pdr8*) were successfully transformed into ADΔΔ cells. Their expression levels were compared with CaCdr1 (Figure 6a). *F. keratoplasticum* Abc1 was used as another control to compare the expression levels with those of another mould species.^23^ FkAbc1 expression levels were ∼28% of CaCdr1 and, as expected, no GFP signal was detected for the negative control strain ADΔΔ (Figure 6a). The expression levels of Pdr1, Pdr3, Pdr5, Pdr6, Pdr7 and Pdr8 were 11%, 48%, 20%, 26%, 63% and 41% of CaCdr1, respectively.

### PDR transporters localized correctly to the plasma membrane

The expected plasma membrane localization of PDR transporters was confirmed by SIM (Figure 6b). The majority of CaCdr1 localized to the plasma membrane with some of the protein visible in the rough endoplasmic reticulum surrounding the cell nucleus. All *M. lusitanicus* PDR transporters showed similar localization patterns with the majority of all proteins localized to the plasma membrane.

### Antifungal susceptibilities of recombinant strains

The potential drug efflux pump activities of the PDR transporters were determined by measuring their antifungal susceptibilities. Also included was *M. lusitanicus* CBS277.49, which proved to be multidrug resistant to the tested drugs (Table S3). The overexpression of *pdr8* caused the most pronounced multidrug resistance phenotype with significantly increased resistance levels observed for all test drugs apart from anidulafungin, which was included as a ‘negative’ control drug, usually not a substrate of drug efflux pumps^59^ (Table 3; Table S3). ADΔΔ cells overexpressing *pdr8* were 48 times more resistant to voriconazole and 32 times more resistant to fluconazole, oteseconazole and isavuconazole, but only 4 and 5 times more resistant to itraconazole and posaconazole, respectively. Overexpression of *pdr1* and *pdr6* also caused multidrug resistance, but their substrate range was limited with a preference for short-length tailed azoles fluconazole and voriconazole, as well as, oteseconazole. The resistance conferred to ADΔΔ by the overexpression of *pdr1* or *pdr6* was ∼2–10 times lower (Table 3) than that of *pdr8*. They were, however, comparable to *pdr8* after accounting for their significantly reduced expression levels. Pdr1 could also transport long-tailed azoles causing 5- and ∼3-fold increased resistance to posaconazole and itraconazole, respectively. In fact, Pdr1 appeared to be the most efficient posaconazole and itraconazole efflux pump (Table S3) after accounting for its ∼4 times lower than Pdr8 expression level. Pdr6 could not transport those two substrates, but it could transport isavuconazole which was not an efflux pump substrate of Pdr1 (Table 3). Pdr7 was the least effective efflux pump. It had a narrow substrate range, only able to transport voriconazole, fluconazole and oteseconazole and even those not as well as Pdr1, Pdr6 or Pdr8 causing only 2–4-fold increased resistance levels against voriconazole, fluconazole and oteseconazole (Table 3) despite its relatively high expression level (61% of Cdr1; Figure 6a). Pdr3 and Pdr5 were not able to transport any of the seven antifungal test substrates causing no significant changes to the drug resistance levels (Table 3).

**Table 3.** Fold changed antifungal susceptibilities of *S. cerevisiae* ADΔΔ cells overexpressing the indicated PDR transporters, *F. keratoplasticum* Abc1 and *C. albicans* Cdr1

	Strains	fold change based on MIC_90_^a^ relative to ADΔΔ						
		FLC^b^ (306)	VRC (349)	ISA (438)	OTC (527)	POS (701)	ITC (706)	AFG (1140)
	ADΔΔ	1	1	1	1	1	1	1
	FkAbc1	32	24	256	512	32	32	1
	CaCdr1	>64	>250	>512	512	43	>43	1
Cluster A	MlusPdr1	8	8	1	6	5	2.7	0.5
	MlusPdr6	16	12	4	8	1.4	1	1
	MlusPdr7	4	4	1	2	1	0.7	1
	MlusPdr8	32	48	32	32	5	4	2
Cluster B	MlusPdr3	0.5	2	1	1	0.7	0.7	2
	MlusPdr5	1	2	1	1.5	1	0.7	1

FLC, fluconazole; VRC, voriconazole; ISA, isavuconazole; OTC, oteseconazole; POS, posaconazole; ITC, itraconazole; AFG, anidulafungin.

^a^The median MIC_90_ values varied no more than ±2-fold. The drug susceptibilities (mg/L) of the sensitive host strain ADΔΔ were as follows: 1 mg/L for FLC; 0.016 mg/L for VRC; 0.0078 mg/L for ISA and OTC; 0.094 mg/L for ITC and POS; and 0.5 mg/L for AFG. MIC_90_ values for all strains are provided in Table S3.

^b^The antifungals are listed in order of increasing molecular weight, shown in brackets underneath each drug.

### ATPase activities of PDR transporters

The CaCdr1 ATPase activity of 170 nmol/min/mg was comparable to previously published results.^36,60^ FkAbc1 had a much lower, but still detectable ATPase activity of 18 nmol/min/mg. *M. lusitanicus* Pdr1, Pdr6, Pdr7 and Pdr8 had ATPase activities of 13, 37, 89 and 42 nmol/min/mg, respectively (Table 4). Their protein expression normalized ATPase activities of ∼120, ∼140 ∼140 and ∼100 nmol/min/mg were comparable to those of CaCdr1. *M. lusitanicus* Pdr3 and Pdr5 had practically no detectable ATPase activities even though their expression levels were similar (Pdr5) or even 2–4 times higher (Pdr3) than Pdr1 and Pdr6.

**Table 4.** PDR transporter-specific ATPase activities of plasma membrane preparations of ADΔΔ cells overexpressing *M. lusitanicus* PDR transporters and *C. albicans* Cdr1 and *F. keratoplasticum* Abc1

	Strains	ATPase activity (nmol/min/mg)	Normalized ATPase^a^ (nmol/min/mg)
	CaCdr1	170 ± 18	170
	FkAbc1	18 ± 3	65
**Cluster A**	MlusPdr1	13 ± 3	120
	MlusPdr6	37 ± 5	140
	MlusPdr7	89 ± 15	140
	MlusPdr8	42 ± 3	100
**Cluster B**	MlusPdr3	7 ± 5	100
	MlusPdr5	Not detectable	Not detectable

Data are the mean of three independent experiments of technical duplicates from at least two separate crude plasma membrane preparations ± standard deviations.

^a^Normalized ATPase activities take the protein expression levels into account (see Figure 6a).

## Discussion

Treatment of mucormycosis is challenging due to the innate resistance of mucormycetes to echinocandins and azoles such as fluconazole or voriconazole.^61^ To the best of our knowledge, there is just one report^34^ on the possible contribution of PDR transporters to azole resistance in *M. lusitanicus* MS12 (*leuA^−^* and *pyrG^−^*).^62^ These authors concluded that *pdr1* and *pdr2* contribute to the innate azole resistance of *M. lusitanicus*. However, deletion of *pdr1* or *pdr2* or both caused only minor (<2-fold) reductions to the drug susceptibilities of cells against posaconazole and isavuconazole, while all knock-out strains remained resistant (>64 mg/L) to fluconazole and itraconazole. This is the disadvantage of gene knock-out studies in the native host because the deletion of efflux pumps is often masked by other efflux pumps with overlapping transport function. The innate azole resistance of *M. lusitanicus* CBS277.49 is likely caused by a combination of factors, including the overexpression of efflux pumps and the presence of the intrinsically azole resistant *cyp51* F5 gene with its characteristic F129 residue.^61^ The deletion of only one or two efflux pumps and the presence of *cyp51* F5 may explain why Nagy *et al.*^34^ observed only minor changes to the azole susceptibilities of the single or double gene knock-out strains. This highlights the importance of testing the potential efflux pump activity of uncharacterized PDR transporters in the drug-hypersusceptible heterologous expression host ADΔΔ devoid of efflux pumps and containing an azole susceptible *ERG11* gene.

Phylogenetic analysis revealed eight *Mucor* PDR transporter orthologs of two distinct clusters, cluster A (*pdr1*, *pdr6*, *pdr7* and *pdr8*) and cluster B (*pdr2*, *pdr3*, *pdr4* and *pdr5*) (Figure 2), all of which have one common ancestor and form a unique sub-cluster, H3, of fungal cluster H PDR transporters (Figure 3a).^57^

As so often is the case for fungal PDR transporters involved in azole resistance,^63–66^  *M. lusitanicus* cluster A PDR transporters *pdr1* and *pdr6* were strongly up-regulated in response to azoles, while the mRNA levels of the four cluster B PDR transporters remained largely unaffected. The *pdr7* and *pdr8* mRNA levels, however, dropped ∼30%–70% in response to either of those three azoles (Figure 4 and Table 2). Perhaps not surprisingly, *pdr1*, *pdr5*, *pdr7* and *pdr8* were the only four orthologs that were conserved in all six *Mucor* species (Figure 2). The elevated basal expression levels of *pdr1*, *pdr5*, *pdr7* and *pdr8* (Table 2) and their preservation in all six *Mucor* species suggests important biological transport functions for these four *M. lusitanicus* orthologs, whereas the much lower expression levels of *pdr2*, *pdr3*, *pdr4* and *pdr6* suggest more specialized transport functions for these orthologs.

A potential limitation of this study is the use of sub-MIC_90_ concentrations (4.0 mg/L) of azoles. Exposing cells to supra-MIC_90_ levels (>32 mg/L) of azoles may possibly elicit a somewhat different response on some of the genes. Azole exposure in ascomycetes such as *S. cerevisiae* or *C. albicans* induces up-regulation of dedicated efflux pumps.^15,67–69^ There are, however, exceptions to this rule. *C. neoformans AFR1* causes azole resistance in clinical isolates but it does not respond to fluconazole exposure.^20^  *C. glabrata CDR1* is another example that does not respond to voriconazole exposure.^70^

Six of the eight *M. lusitanicus* PDR transporters were successfully expressed in *S. cerevisiae* ADΔΔ.^35^ However, *M. lusitanicus pdr2* and *pdr4* cDNA ORFs could not be amplified, possibly because of their low expression level (*pdr2*; ΔCq = 16.2) or for some other unknown reason (*pdr4*). Antifungal drug susceptibilities of the PDR transporter overexpressing cells revealed Pdr1, Pdr6, Pdr7 and Pdr8 (cluster A) as potential contributors to the innate azole resistance phenotype of *M. lusitanicus*. All four PDR transporters caused increased MIC_90s_ for fluconazole and voriconazole. Pdr1, Pdr6 and Pdr8 appear to be efficient efflux pumps of short-tailed azoles. Pdr7 is a less efficient fluconazole and voriconazole transporter and it also has a much narrower substrate range (Table 3). Pdr8 was the most efficient multidrug efflux pump conferring resistance against all six azoles tested (4–48-fold). Pdr1 was also a very prolific multidrug efflux pump protecting cells against five of the six azoles apart from isavuconazole. But Pdr1 was the most efficient posaconazole and itraconazole efflux pump: the normalized resistance levels of Pdr1 overexpressing cells were 45- and 25-fold compared to 12- and 10-fold, respectively, increased in Pdr8 overexpressing cells (Table S4). Pdr6 was an efficient efflux pump of all short- and mid-length azoles but it was unable to efflux long-tailed azoles posaconazole and itraconazole (Table 3). Overexpression of *M. lusitanicus* Pdr3 and Pdr5 had no significant effect on the drug susceptibilities and neither protein had any measurable ATPase activity. However, all cluster A PDR transporters had ATPase activities (≥60%) comparable to CaCdr1 (Table 4).

Characterization of *M. lusitanicus* PDR transporters in the host ADΔΔ revealed that all four cluster A PDR transporters are potential multidrug efflux pumps. The high expression levels of *pdr1*, *pdr5* and *pdr7* (Table 2) indicate important transport functions during normal *M. lusitanicus* growth. The dramatic up-regulation of *pdr1* (∼10-fold) and *pdr6* (∼100-fold) upon azole exposure and the fact that both transporters are efficient efflux pumps suggests that Pdr1 and Pdr6 are likely the two major multidrug efflux pumps that protect *M. lusitanicus* against azoles. Even though Pdr8 was the most efficient efflux pump in *S. cerevisiae*, its rather low expression level and the fact that it is further down-regulated upon azole exposure makes Pdr8 less likely to contribute significantly to azole resistance of *M. lusitanicus*, although it is possible that *pdr8* is up-regulated during host invasion and it might, therefore, also contribute to the innate azole resistance phenotype. There was one additional ABC transporter that may contribute to the azole resistance phenotype of *M. lusitanicus*. The ∼4-fold induced expression levels of *abcb1* (Figure 5c) reached levels that were as high as those of *pdr1* and *pdr6* in response to azole exposure (Table 2).

To summarize, *Mucor* cluster A PDR transporters are multidrug efflux pumps with Pdr1 and Pdr6, and possibly also Abcb1, being the major efflux pumps contributing to the innate azole resistance phenotype of *M. lusitanicus*.

## Supplementary Material

dkaf343_Supplementary_Data

## Data Availability

All RNA-sequencing raw data are available from the NCBI Short Read Archive (SRA) under the BioProject number PRJNA1075823 with accession numbers SRX23605683, SRX23605684, SRX23605685, SRX23605686, SRX23605687, SRX23605688, SRX23605689, SRX23605690, SRX23605691, SRX23605692, SRX23605693 and SRX23605694. The plasmid sequences are available from GenBank with the accession numbers PQ407573 (pABC3-blaster), PQ407574 (pABC3-GFP3-blaster), PQ407575 (pABC3-mRFP-blaster), PQ407576 (pABC3-XLmGFPHis) and PQ407577 (pABC3XL).

## Supplementary data

Figures S1–S5, Tables S1–S4 and Files S1–S5 are available as Supplementary data at *JAC* Online.
