# Supplementary material for: Multidrug efflux pumps and innate azole resistance of Mucor lusitanicus
Source: J Antimicrob Chemother. 2025 Sep 30;80(11):3065–78. doi: 10.1093/jac/dkaf343 (PMC12596049; doi:10.1093/jac/dkaf343)
Supplement: dkaf343_Supplementary_Data [file dkaf343_supplementary_data.zip › JAC_MlusPDR Supplementary File 1_2_Figures and Tables_Revision_250730.docx]

Supplementary Material

# Supplementary Tables

**Table S1**. Fungal strains used in this study.

| Strain | Genotype | Reference |
| --- | --- | --- |
| *Mucor lusitanicus* CBS277.49 | Clinical isolate, azole resistant | ^1^ |
| *S. cerevisiae* ADΔ/CaCDR1-GFP | ADΔ, ∆*pdr5*::pABC3-CaCDR1A-GFP | ^2^ |
| ADΔΔ | ADΔ, ∆*his1* | ^3^ |
| ADΔΔ/FkABC1-XLmGFPHis | ADΔΔ, ∆*pdr5*::pABC3-*Fker-abc1*-*XLmGFPHis* | ^4^ |
| ADΔΔ/MlusPDR1-XL | ADΔΔ, ∆*pdr5*::pABC3XL-*Mlus*-*pdr1* | This study |
| ADΔΔ/MlusPDR3-XL | ADΔΔ, ∆*pdr5*::pABC3XL-*Mlus*-*pdr3* | This study |
| ADΔΔ/MlusPDR5-XL | ADΔΔ, ∆*pdr5*::pABC3XL-*Mlus*-*pdr5* | This study |
| ADΔΔ/MlusPDR6-XL | ADΔΔ, ∆*pdr5*::pABC3XL-*Mlus*-*pdr6* | This study |
| ADΔΔ/MlusPDR7-XL | ADΔΔ, ∆*pdr5*::pABC3XL-*Mlus*-*pdr7* | This study |
| ADΔΔ/MlusPDR8-XL | ADΔΔ, ∆*pdr5*::pABC3XL-*Mlus*-*pdr8* | This study |

**Table S2.** DNA oligomer primers used in this study.

| **Name**^a^ | **Sequence (5’ to 3’)^b^** |
| --- | --- |
| Housekeeping gene | |
| MlusGAPDH-F102* | GTCAAGGTCGTCGCTATCAAT |
| MlusGAPDH-R184* | ACCAGTTTACCGTCCTTGTG |
| PDR transporters | |
| MlusPDR1-PacI-F | CGCTCGTTCGAAAGACTTAATTAAAAAATGACTTCCACTCATCATCTCCAAG |
| MlusPDR1-F29* | CTTCCACTCATCATCTCCAAGGATC |
| MlusPDR1-R98* | TTTCAAATTCGTGGAGGCCGT |
| MlusPDR1-R165 | CACATATTCGTCTTTGGCGGCATCG |
| MlusPDR1-F673 | TACCGAGGCCAAGTCTGTTACAACG |
| MlusPDR1-F1312 | TACCTTGCCTCTGATGTCTACAAGC |
| MlusPDR1-F1935 | TACCACCAGTTTCTTCTTGGCTACG |
| MlusPDR1-F2588 | ATCTCACAGCGTTGATGGGCTCCTC |
| MlusPDR1-F3269 | TCCTTGAGTGTGTGGGTGCCGGTAC |
| MlusPDR1-F3852 | TTCCATGCTCGTCTGTTGGGCTGTC |
| ppABC3-MlusPDR1-X-R | CCTTGGAACAAGACTTCCAAGCGGCCGCCACGTCTAGGCTTTCTAAAGACATAA |
| MlusPDR2-PacI-F | CGCTCGTTCGAAAGACTTAATTAAAAAATGGATATGCCATCAGCGATTGAAG |
| MlusPDR2-F57* | GGCCAATTACCCTAACCGAGATGAT |
| MlusPDR2-R162* | CTGCGAGTCTCTGATCTCTCCAAA |
| MlusPDR2-R173 | CCACCTTCCGTTCTGCGAGTCTCTG |
| MlusPDR2-F629 | GAGAAATGCTCCTGGTATTGGGTCG |
| MlusPDR2-F1277 | ACATGGGCTTCTACTGTCCTGATCG |
| MlusPDR2-F1873 | GCTGACATTCCTTTGGCGTTAATCC |
| MlusPDR2-F2538 | TACCGTCAACGACCAAGAGATGGAG |
| MlusPDR2-R2664 | TTTGCCAGCACCAGAAGAGCCCATC |
| MlusPDR2-F3159 | TGGCTGGCCCGTGCTTTGTACTATC |
| MlusPDR2-F3758 | GAATGTGGTTCAGACGTGAGTATGC |
| ppABC3-MlusPDR2-X-R | CCTTGGAACAAGACTTCCAAGCGGCCGCCGCGCTTTTGTTTTCTGAACAAAAAG |
| MlusPDR3-PacI-F | CGCTCGTTCGAAAGACTTAATTAAAAAATGATTGATCCCATAGCTCGTACCT |
| MlusPDR3-F29* | TGATCCCATAGCTCGTACCTACTC |
| MlusPDR3-R107* | GCATTAGATCGCTCCGTCAATGTTC |
| MlusPDR3-F621 | TCTGCATTACCCTACCTTGACTACG |
| MlusPDR3-F1247 | CTCAAATGATGCGTGAACGTGATGC |
| MlusPDR3-F1923 | GCATCCTTGGCTCATGTGGATTTAC |
| MlusPDR3-F2569 | GGCAACATCTACATGAACGGCGAAC |
| MlusPDR3-F3118 | GGTGGTCCTGTCTGTGCTCCTGAAG |
| MlusPDR3-F3823 | ATCAACCCGTTCTTCCTCACGGTGC |
| ppABC3-MlusPDR3-X-R | CCTTGGAACAAGACTTCCAAGCGGCCGCCTCGCTTTTGCTTTCTAAATGCGTAT |
| MlusPDR4-PacI-F | CGCTCGTTCGAAAGACTTAATTAAAAAATGGCATATCAAAACAGAATCAGTA |
| MlusPDR4-F45* | CAGTAATGACAGCACAAGCCGATCT |
| MlusPDR4-R99* | CGAGACTGAGATTGAGAGAGCTGA |
| MlusPDR4-F565 | TGCTCTACTCTTCTCCGCCTCTTGG |
| MlusPDR4-F1267 | CAAGACCGCCAAGTCCCAATGAATG |
| MlusPDR4-F1813 | CTTATGGGTCTCGCTTCTGATGCTG |
| MlusPDR4-F2438 | AACTTGAAGCAGTGTCGGGCGGAAC |
| MlusPDR4-F3106 | TTGTTACTTCTGATGCGAGGCGGTC |
| MlusPDR4-F3783 | AAACGTGTCTGAGCGCGTGGGCTAC |
| ppABC3-MlusPDR4-X-R | CCTTGGAACAAGACTTCCAAGCGGCCGCCTCGCTTTTGCTTTCTAAACAAGTAC |
| MlusPDR5-PacI-F | CGCTCGTTCGAAAGACTTAATTAAAAAATGGATATGCCAGCCTTGCAAGGTA |
| MlusPDR5-F119* | GAAGGCGTTCACGGTAAAGAAGA |
| MlusPDR5-R159* | TTCACCGAAAGCACCGCCATTA |
| MlusPDR5-R248 | CGGCGAGAGATGCGAGATTGCTGTG |
| MlusPDR5-F610 | GGTGCTGGTTGTACTTCTCTGTTGC |
| MlusPDR5-F1238 | TCTTCTGTCCTAGCCGTAAATCCAC |
| MlusPDR5-F1873 | GTCTACTTTATGATGGGCCTTGTGC |
| MlusPDR5-F2568 | TGTCAAGGGCGGCAAACTCAAGCTC |
| MlusPDR5-F3257 | AATCCAACGGTGGTCCCAAGTGCTC |
| MlusPDR5-F3855 | TGTGTCTGACCGTGTAGGCTTCTTC |
| ppABC3-MlusPDR5-X-R | CCTTGGAACAAGACTTCCAAGCGGCCGCCTCGCTTGGCCTTTCTGAATAAGAAG |
| MlusPDR6-PacI-F | CGCTCGTTCGAAAGACTTAATTAAAAAATGGAAGAAAAACCAACTGAACACA |
| MlusPDR6-F52* | AGGTGCCTCATCACCATCCGAATT |
| MlusPDR6-R107* | CTGTCTCGCCATAAGTGCCATTTC |
| MlusPDR6-R226 | CTGAATCTGCCTGTCCTTCCTCAAC |
| MlusPDR6-F667 | AGAGGCCAAGTGTGCTACAATGAAG |
| MlusPDR6-F1262 | AAGACTCGGCTCCCAAGCACGCTTC |
| MlusPDR6-F1927 | ACCTCCAGTTTCTTCTTGGCTACTC |
| MlusPDR6-F2618 | CCACCTTGCTTGATGTACTTGCTCG |
| MlusPDR6-F3254 | AGTATATTCTCGAAGTGGTGGGTGC |
| MlusPDR6-F3888 | TGCTGTAGCTGAACTGCCTACCATG |
| ppABC3-MlusPDR6-X-R | CCTTGGAACAAGACTTCCAAGCGGCCGCCACGTCTACCCTTTCTTCTCAAGTAA |
| MlusPDR7-PacI-F | CGCTCGTTCGAAAGACTTAATTAAAAAATGGAGGAGAAAAAAAACGCTCTAT |
| MlusPDR7-F130* | GTCAGTGCCCACAACAAGGAGAATT |
| MlusPDR7-R238* | CCTCAGCTTTGGAGAGTGATAAACG |
| MlusPDR7-F641 | TTGGCGGTGATGTGAGCTACGGTGG |
| MlusPDR7-F1247 | CTGGTCTCTGTAATCCTCTGGAACG |
| MlusPDR7-F1875 | TGGCAGATTCTTCACCTCGTTCCTC |
| MlusPDR7-F2422 | GCTCCCAAGCCTCGTACTACTGAAG |
| MlusPDR7-F3114 | CATCCATCAGCCTTCTGCCATCTTG |
| MlusPDR7-F3827 | GTATGCGAAACACACCCGCAGCTTG |
| ppABC3-MlusPDR7-X-R | CCTTGGAACAAGACTTCCAAGCGGCCGCCTCTTCTGCCCTTTCTCTTCCAGTAA |
| MlusPDR8-PacI-F | CGCTCGTTCGAAAGACTTAATTAAAAAATGTCAAATCAGAAATTAGAGCTGT |
| MlusPDR8-F34* | AATTAGAGCTGTCTGACGCCA |
| MlusPDR8-R94* | GGTGCCTTCTTCTTGAGATTCCGA |
| MlusPDR8-R204 | AAACTCATCAATCGCAGCTTCCACG |
| MlusPDR8-F648 | CATTTCCAACATGCGAGGCTCATTC |
| MlusPDR8-F1196 | TGTACTTTGGTCCGGTGGCCTCTGC |
| MlusPDR8-F1801 | CGTCCTTCTGCATTCTACCTTGCTC |
| MlusPDR8-F2477 | CGCAAGAAGAGATTGCAGACCAACG |
| MlusPDR8-F3292 | CCCAAGTGCTCGCCTGCTGCTAATC |
| MlusPDR8-F3927 | TTGGGCTGTTACACTAGGCTTTGTC |
| ppABC3-MlusPDR8-X-R | CCTTGGAACAAGACTTCCAAGCGGCCGCCACGTCTTCCCTTACGCTTCCAGTAC |
| pABC3 vector and *S. cerevisiae* AD∆∆ sequences | |
| PDR5-up | GCATAAAACAGAGAGGCGATATAGG |
| PDR5-promoter | CACACACATATATATAAGCCTGAATGC |
| pABC3-for | ATAAATTGGCAACTAGGAACTTTCG |
| pABC3-GFP-R87 | AGCATCACCTTCACCTTCACCGGAG |
| pABC3-GFP-F329 | ATGACGGTAACTACAAGACCAGAGC |
| pABC3-up-F | GAATTGTAATACGACTCACTATAGGGCG |
| pABC3-PmeI-R | CTTTG**G**TTT**A**AACTTTTGTTCTCTCTCTCAAC |
| pABC3-PmeI-F | GTTGAGAGAGAGAACAAAAGTT**T**AAA**C**CAAAG |
| pABC3-PDR5TGFPHIS-SacI-R | ATTCTATTATTTCTGGAGCTCATTTAATGATGATGGTGATGATGACTGCC |
| pABC3-PDR5T-SacI-F | AAATGAGCTCCAGAAATAATAGAATTTTGAATTTGGTTAA |
| pABC3-PDR5T-BamHI-R | CACCGGATCCTTTCGGACATTGAACTTTGATTTATC |
| pABC3-URA3-R402 | CTTTGTTACTTCTTCTGCCGCCTGC |
| PDR5ter | TTTAGGCACTCTTGCTAACCAGTAGA |
| PDR5-down | AGAAGACGGTTCGCCATTCGGACAG |
| Fragment amplification | |
| pABC3-up-F/pABC-PmeI-R | Fragment A |
| pABC3-PmeI-F/pABC3-PDR5TGFPHIS-SacI-R | Fragment B |
| pABC3-PDR5T-SacI-F/pABC3-PDR5T-BamHI-R | Fragment C |

^a^ Numbers indicate the ORF position that the 3’ end of the primer binds to.

^b^ Underlined are parts of *S. cerevisiae PDR5* genomic regions or novel restriction sites of pABC3XL, and in red are the *Pac*I/*Not*I cloning sites.

* Primers used for qPCR.

F/for stands for forward; R/rev stands for reverse

Up = upstream; down = downstream; ter = terminator

**Table S3.** Antifungal susceptibilities of *S. cerevisiae* AD∆∆ cells overexpressing the indicated PDR transporters including the clinically resistant *M. lusitanicus* CBS277.49 isolate.

| Strains | MIC_90_ [mg/L]^*^ | | | | | | |
| --- | --- | --- | --- | --- | --- | --- | --- |
|  | FLC | VRC | ISA | OTC | ITC | POS | AFG |
| ADΔΔ | 1 | 0.016 | 0.0078 | 0.0078 | 0.094 | 0.094 | 0.5 |
| -FkAbc1 | 32 | 0.38 | 2 | 4 | 3 | 3 | 0.5 |
| -CaCdr1 | >64 | >4 | >4 | 4 | >4 | 4 | 0.5 |
| -MlusPdr1 | 8 | 0.125 | 0.0078 | 0.047 | 0.25 | 0.5 | 0.25 |
| -MlusPdr6 | 16 | 0.19 | 0.031 | 0.063 | 0.094 | 0.125 | 0.5 |
| -MlusPdr7 | 4 | 0.063 | 0.0078 | 0.016 | 0.063 | 0.094 | 0.5 |
| -MlusPdr8 | 32 | 0.75 | 0.25 | 0.25 | 0.38 | 0.5 | 1 |
| -MlusPdr3 | 0.5 | 0.031 | 0.0078 | 0.0078 | 0.063 | 0.063 | 1 |
| -MlusPdr5 | 1 | 0.031 | 0.0078 | 0.012 | 0.063 | 0.094 | 0.5 |
| CBS277.49^**^ | >16 | >16 | >16 | >4 | >8 | >16 | 16^***^ |

Abbreviations: FLC = fluconazole, VRC = voriconazole, ISA = isavuconazole, OTC = oteseconazole, ITC = itraconazole, POS = posaconazole, AFG = anidulafungin.

*The median MIC_90_ values shown varied no more than ± 2-fold. The test concentration range [mg/L] of the antifungals used for the recombinant AD∆∆ strains was as follows: 0.125 – 64.0 for FLC; 0.0078 – 4.0 for VRC, ISA, OTC, ITC, POS; and 0.0312 – 16.0 for AFG.

**The test concentration range [mg/L] of the antifungals used for *M. lusitanicus* CBS277.49 were: 0.0312 – 16.0 for FLC, VRC, ISA, POS and AFG; 0.0078 – 4.0 for OTC, 0.0156 – 8.0 for ITC. The internal quality control strain *Aspergillus flavus* (ATCC 204304) was within the target range recommended by EUCAST^5^.

***Endpoint susceptibilities of minimum effective concentrations (MEC) that caused morphological changes to the fungus.

**Table S4.** Normalised fold increased antifungal susceptibilities of *S. cerevisiae* AD∆∆ cells overexpressing *F*. *keratoplasticum* Abc1, *C. albicans* Cdr1, or *M. lusitanicus* cluster A PDR transporters.

| Strains  (% Cdr1 expression) | fold change based on MIC_90_^*^ relative to AD∆∆ | | | | | | |
| --- | --- | --- | --- | --- | --- | --- | --- |
|  | FLC**  (306) | VRC  (349) | ISA  (438) | OTC  (527) | POS  (701) | ITC  (706) | AFG  (1140) |
| ADΔΔ | 1 | 1 | 1 | 1 | 1 | 1 | 1 |
| -FkAbc1 (28) | >64 | 86 | >512 | 1830 | 114 | >43 | 1 |
| -CaCdr1 (100) | >64 | >250 | >512 | 512 | 43 | >43 | 1 |
| -MlusPdr1 (11) | 73 | 73 | 1 | 55 | 45 | 25 | 0.5 |
| -MlusPdr6 (26) | 62 | 46 | 15 | 31 | 2.5 | 1 | 1 |
| -MlusPdr7 (63) | 6 | 6 | 1 | 3 | 1 | 0.7 | 1 |
| -MlusPdr8 (41) | 78 | 117 | 78 | 78 | 12 | 10 | 2 |

^*^The median MIC_90_ values varied no more than ± 2-fold. The drug susceptibilities [mg/L] of the sensitive host strain AD∆∆ were as follows: 1 mg/L for FLC; 0.016 mg/L for VRC; 0.0078 mg/L for ISA and OTC; 0.094 mg/L for ITC and POS; and 0.5 mg/L for AFG.

^**^The antifungals are listed in order of increasing molecular weight, shown in brackets underneath each drug. Abbreviation: FLC fluconazole, VRC voriconazole, ISA isavuconazole, OTC oteseconazole, POS posaconazole, ITC itraconazole, AFG anidulafungin.

# Supplementary Figures


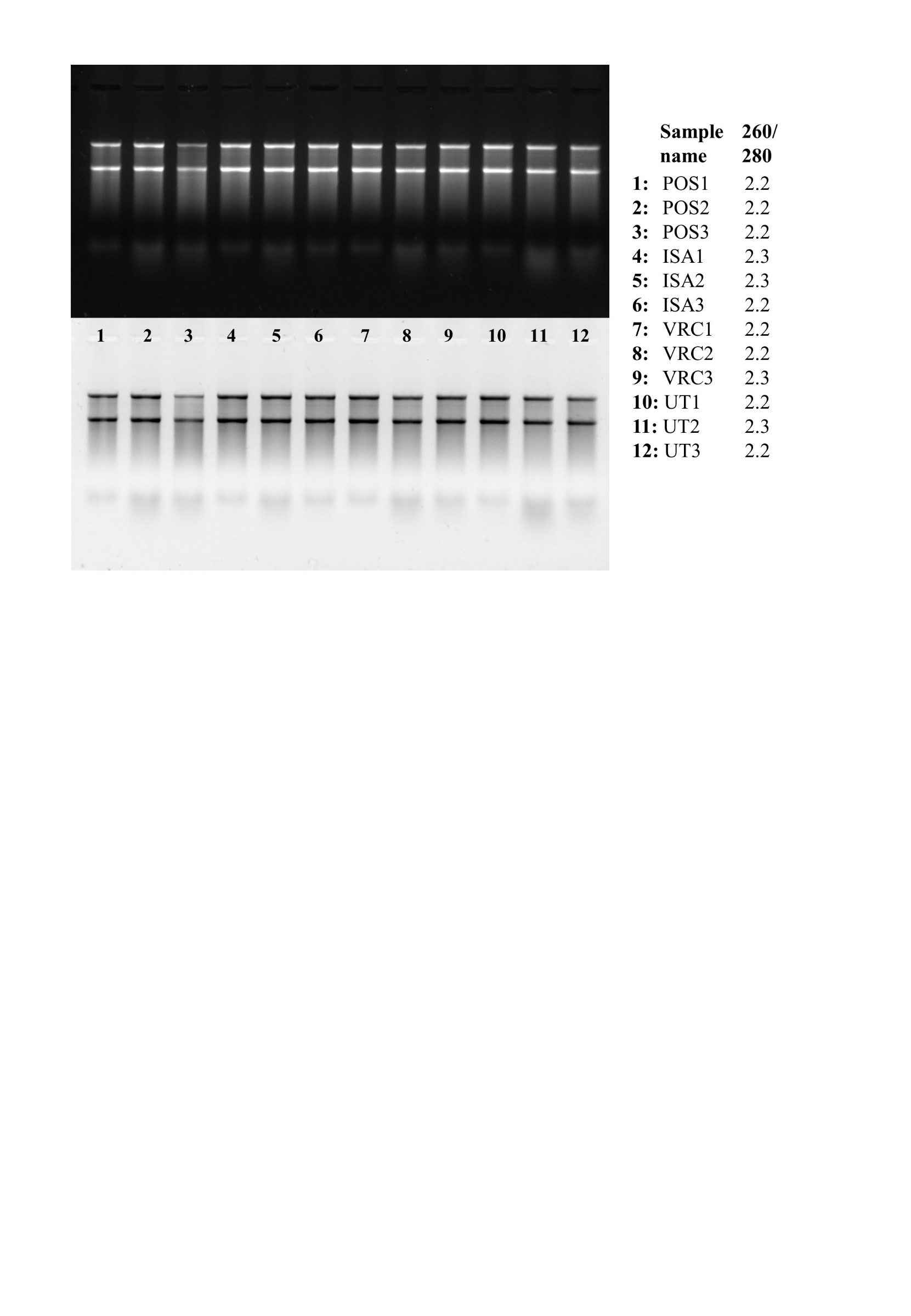


**Figure S1.** **Total RNA samples (1.0 μg each) of *M. lusitanicus* CBS277.49** log-phase cells grown for 80 min in the presence of 4.0 mg/L POS (1-3), ISA (4-5) and VRC (7-9) or total RNA samples of untreated *M. lusitanicus* control (UT, 10-12) cells separated by formaldehyde gel electrophoresis which ensures the complete denaturation of RNA secondary structure. Abbreviation: VRC voriconazole, ISA isavuconazole, POS posaconazole.


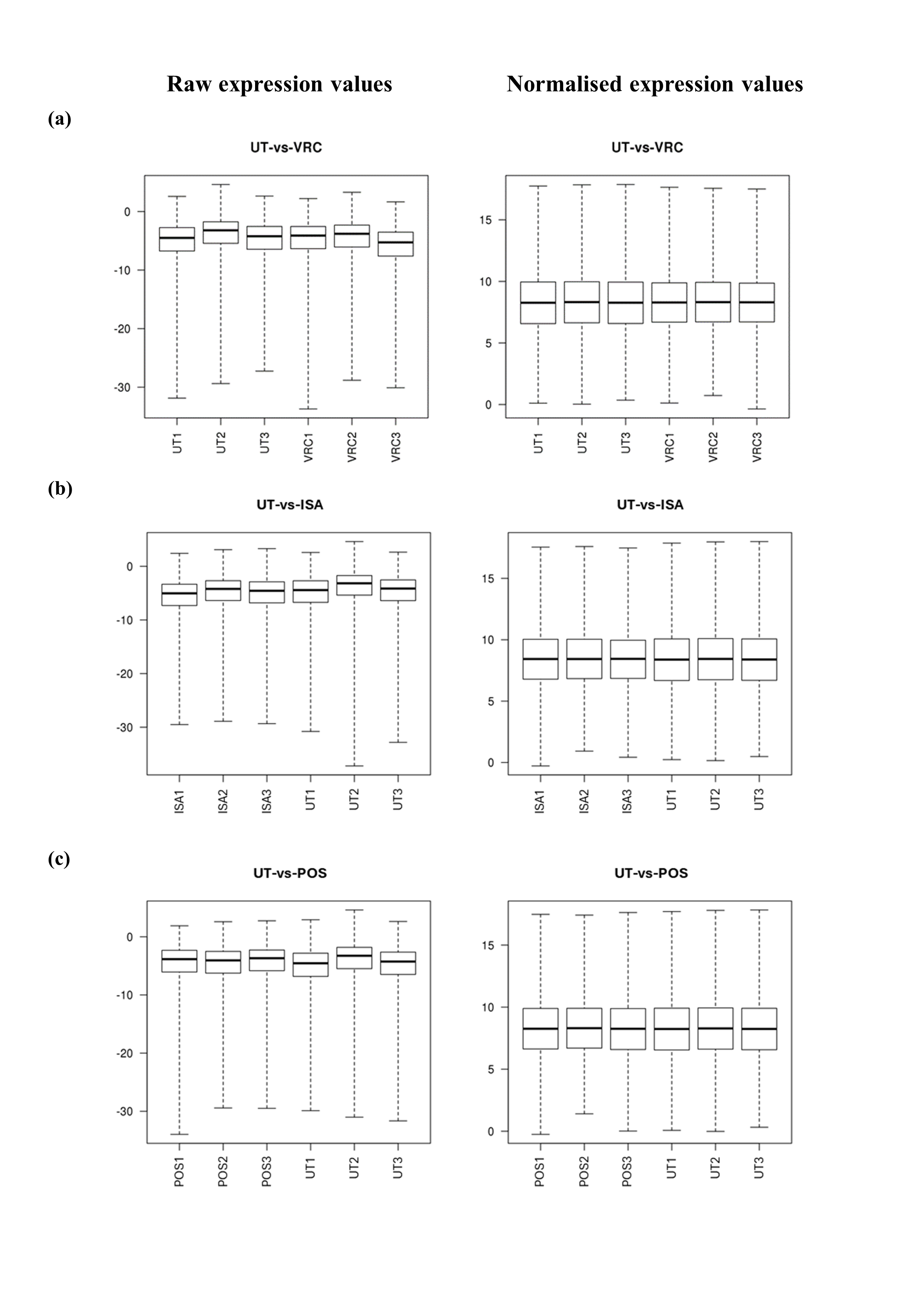


**Figure S2**. **Box plot analysis** showing comparisons between the raw (left) and normalised (right) expression values of 4.0 mg/L (**a**) VRC, (**b**) ISA and (**c**) POS treated samples and the untreated control (UT). Abbreviation: VRC voriconazole, ISA isavuconazole, POS posaconazole.


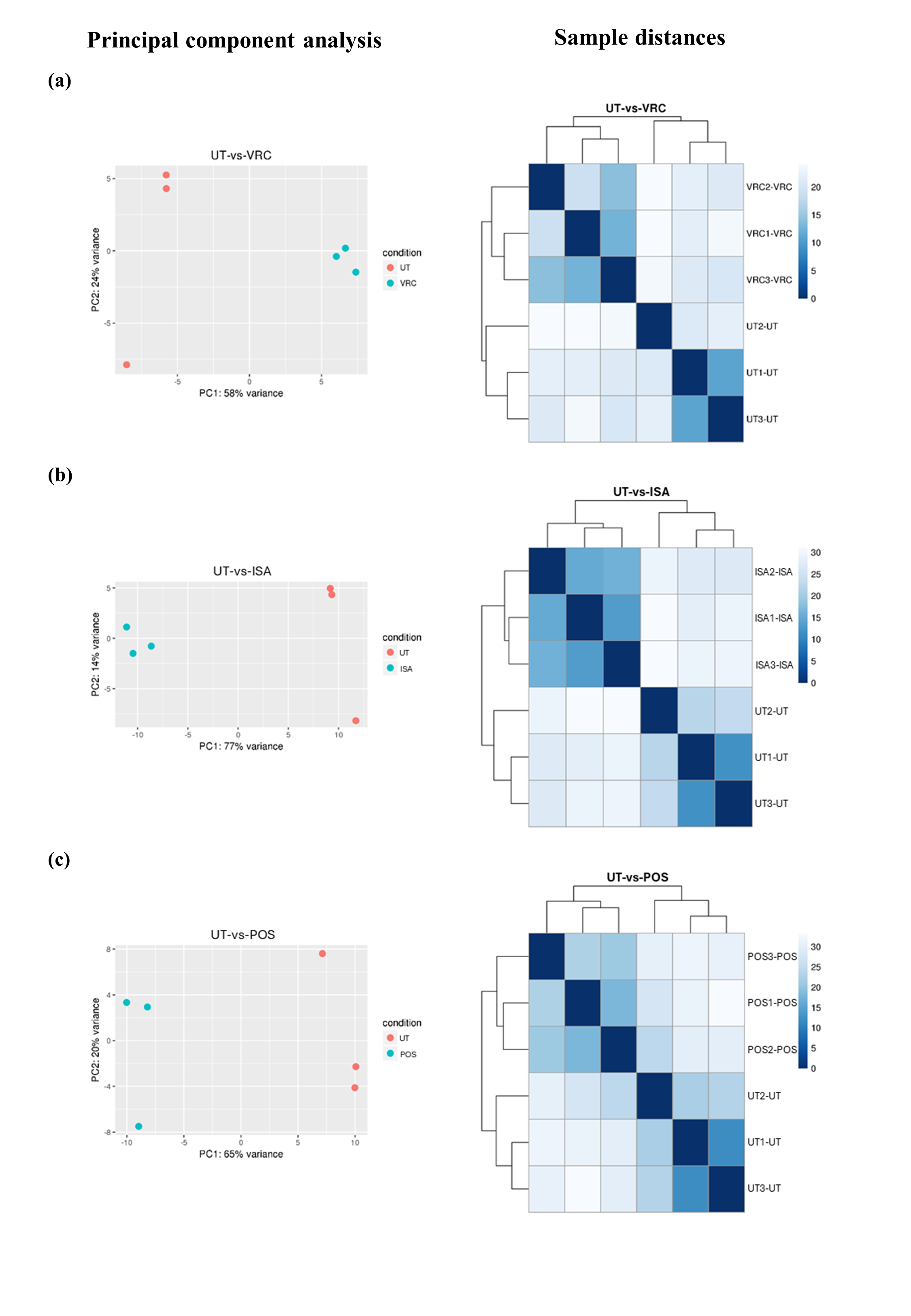


**Figure S3.** **Principal component analysis (left) and sample distances (right)** demonstrating the similarity within and between treatment groups: 4.0 mg/L (**a**) VRC, (**b**) ISA and (**c**) POS treated vs. untreated control (UT). Abbreviation: VRC voriconazole, ISA isavuconazole, POS posaconazole.

**
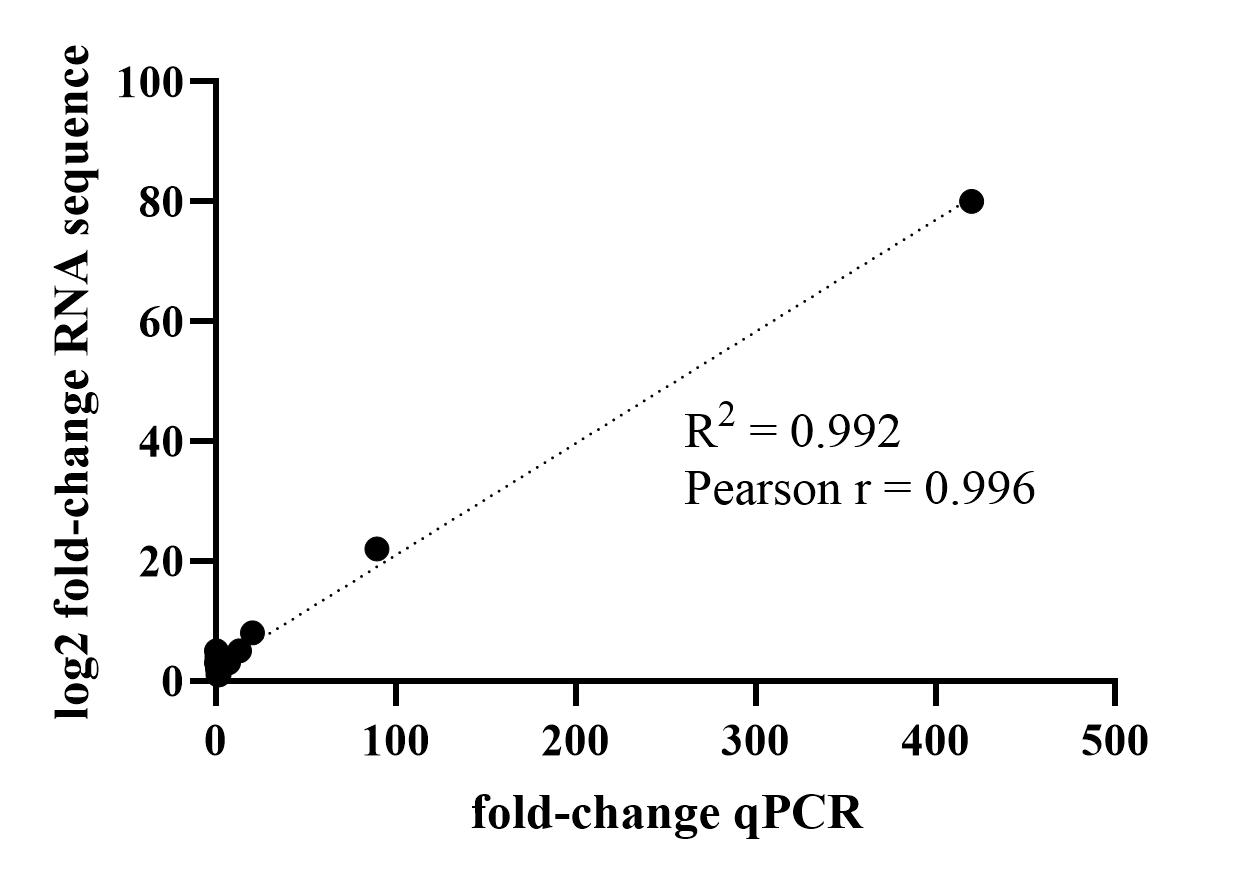
**

**Figure S4**. **There was a strong correlation between the mRNA expression levels determined by sequencing the entire transcriptome or by RT-qPCR.** The Y-axis are the X-fold increased mRNA expression levels determined by RNA-sequencing and the X-axis are the X-fold increased mRNA expression levels of the same PDR transporters determined by RT-qPCR.


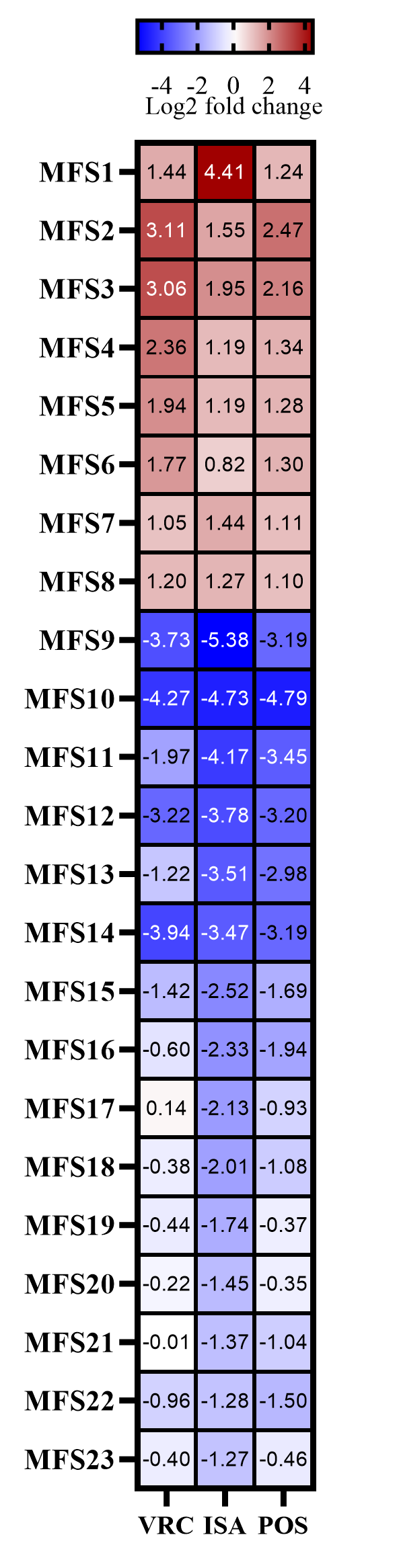


**Figure S5. Significantly DEGs of *M. lusitanicus* MFS transporters.** Heatmaps (column-clustered) of the RNA-seq log2 fold changes determined for all DEGs encoding MFS transporters of *M. lusitanicus* CBS277.49 log-phase cells exposed to 4.0 mg/L VRC, ISA and POS for 80 min. Abbreviation: VRC voriconazole, ISA isavuconazole, POS posaconazole.

# Supplementary File 1 Material and Methods

**This section provides a detailed description of the methods used in this study.**

# S1 Strains and culture conditions

*Mucor lusitanicus* CBS277.49 was grown on yeast peptone glucose (YPG) agar medium containing, in weight/volume, 0.3 % yeast extract (Formedium Ltd, Norfolk, UK), 1 % peptone (Formedium), 2 % glucose (Sigma-Aldrich, MO, USA) and 1.5 % agar (VWR, OH, USA). YPG plates inoculated with three drops of 10 µL spore suspensions (~ 10^8^ cfu/mL each) were incubated at 28 °C in the presence of an artificial light source for 7 d. For total RNA extraction, spores were harvested from the 7 d old YPG plate cultures by gently resuspending the mycelial cells in ~ 8 mL sterile saline (0.9 % NaCl) and scraping them off the plate surface with the end of a 1 mL pipette tip. Large hyphal fragments were removed by filtering the cell suspension through a 40 µm cell strainer (pluriSelect Life Science, Leipzig, Germany). Spores were harvested by centrifugation for 5 min at 8,000 × g and cells of a 1:100 dilution of the inoculum were counted using a haemocytometer. For the azole exposure experiments aliquots of the above spore suspensions were used to inoculate 50 mL YPG liquid medium to a cell density of 2 × 10^4^ cfu/mL.

*Saccharomyces* *cerevisiae* ADΔΔ^3^ was maintained on yeast peptone glucose (YPD, 1 % yeast extract, 2 % peptone, 2 % glucose) agar (1.5 %) plates. *Saccharomyces* *cerevisiae* ADΔΔ uracil prototroph transformants were selected on CSM-URA agar plates containing 0.077 % yeast complete supplement mixture without uracil (Formedium), 0.63 % yeast nitrogen base without amino acids (Formedium), 2 % glucose and 1.5 % agar. Agar plates were incubated at 28 °C for two to three days until colonies became clearly visible on the plate surface. A list of all the yeast strains used in this study is provided in **Table S1**.

# S2 PDR transporter inventories of six *Mucor* species representatives

Full-size PDR transporters were identified with a BLAST search of the *M.* *lusitanicus* CBS277.49 genome^6^ using *S.* *cerevisiae* S288C Snq2, Pdr5 and YOL075C as queries. We also searched for closely related homologs in five other *Mucor* species namely *M. circinelloides* *f.* *circinelloides* 1006PhL^7^, *M.* *ambiguus* NBRC6742, *M. racemosus* UBOCC-A-109155, *M. endophyticus* UBOCC-A-113049 and *M.* *lanceolatus* UBOCC-A-109153^8^. The whole genome sequences are publicly accessible from the MycoCosm^9^ database of the Joint Genome Institute (JGI), date of access: 22 October 2022. A list of all manually curated *Mucor* PDR transporter sequences (some had obviously wrongly predicted start codons and/or intron/exon predictions) is provided in **Supplementary File 2**.

# S3 Isolation of total RNA and RT-qPCR

*Mucor lusitanicus* CBS277.49 cells were grown in 50 mL YPG medium for 9.5 h to early log-phase and incubated for a further 80 min in the absence or presence of either 4.0 mg/L voriconazole, isavuconazole (both kindly provided by Pfizer Inc., NJ, USA) or posaconazole (Sigma-Aldrich), respectively. An equal volume of DMSO (Sigma-Aldrich) was added to the control culture grown in the absence of azoles. Total RNA was extracted with the hot-phenol extraction method using the protocol developed for *F. keratoplasticum*.^10^ RNA concentration was determined using a NanoPhotometer NP80 (Implen GmbH, Munich, Germany), and RNA integrity was confirmed by separating 1 µg total RNA samples using formaldehyde agarose gel electrophoresis (**Fig. S1**).

First strand cDNA was synthesised from 1 µg total RNA using the LunaScript RT SuperMix Kit (NEB, Frankfurt, Germany) following the manufacturer’s instructions. Expression levels of *pdr* genes were measured by RT-qPCR performed with the CFX96 Touch Real-Time PCR Detection System (Bio-Rad Laboratories Inc., Hercules, CA, USA). The qPCR assays contained 1X Luna Universal qPCR Master Mix (NEB), 400 nM forward and reverse primers each and cDNA templates derived from 5 ng total RNA. Thermal cycling steps included a 95 °C denaturation step for 60 s followed by 40 cycles of denaturation at 95 °C for 15 s and DNA synthesis at 60 °C for 30 s. Negative controls without cDNA template were included by adding an equal volume of H_2_O and a total RNA sample but without the addition of reverse transcriptase. An average quantification cycle (Cq) value for each sample was calculated from two technical replicates. mRNA transcript levels (2^–ΔCq^) were normalised to the GAPDH housekeeping gene, *gpd3*. The fold change values of mRNA expression levels relative to cells grown in the absence of azoles was calculated using the ΔΔCq method (2^–ΔΔCq^)^11^. DNA oligonucleotide primers used for RT-qPCR and the amplification, cloning and sequencing of the entire cDNA ORFs of *M. lusitanicus* PDR transporters *pdr1 – 8* are listed in **Table S2**.

# S4 RNA sequencing, transcriptome analysis and identification of DEGs

Twelve RNA samples (three biological replicates for each of the four growth conditions) were sent for RNA sequencing at GENEWIZ Germany GmbH, Leipzig, Germany. The service provided was the strand-specific RNA-seq on Illumina Novaseq 6000, 150 bp paired-end reads with an approximate output of 20 million reads per sample. RNA-sequence data were initially evaluated using FastQC (v.0.11.5) and sequence reads were trimmed using Trimmomatic (v.0.36) to remove adapter sequences. The clean RNA-sequence data for the 12 biological replicates are accessible at the NCBI Short Read Archive (SRA) with BioProject accession number PRJNA1075823. The trimmed reads were mapped to the reference genome *M. lusitanicus* CBS277.49 available on ENSEMBL using the STAR aligner (v.2.5.2b). Unique gene hit counts were calculated by using featureCounts from the Subread package v.1.5.2. Only unique reads that fell within exon regions were used for differential expression analysis using DESeq2 (v1.16.1). Box plot analysis was used to compare expression values between each of the azole-treated samples before and after normalisation (**Fig. S2**). The similarity within and between each of the treatment groups versus the untreated group were assessed with the principal component analysis and calculating the samples distances (**Fig. S3**). The Wald test was used to generate p-values and log2 fold change values, and significant DEGs were classified as those with an adjusted p-value <0.05 and log2-fold change >|1|. A list of all normalised counts calculated from raw data counts, their sequence identifiers and their mRNA expression levels ordered from most up- to most downregulated genes, in *M. lusitanicus* CBS277.49 cells grown in the absence (UT) or presence of voriconazole, isavuconazole and posaconazole is provided in **Supplementary File 3**.

# S5 Creation of plasmids pABC3-, pABC3-GFP3- and pABC3-mRFP-blaster and recycling of the *URA3* marker

Blaster derivatives of pABC3, pABC3-GFP3 and pABC3-mRFP^2^ were created for the convenient removal and possible recycling of the *URA3* marker from uracil positive transformants. The *URA3* blaster technology provides an easy method to remove the *URA3* marker from uracil positive yeast transformants.^12-14^ To create blaster versions of these plasmids, the *S. cerevisiae* *PGK1* terminator between the unique *Not*I and *Bam*HI sites was replaced with the 196 bp *PDR5* terminator flanked by the same *Not*I and *Bam*HI sites. The 196 bp *PDR5* terminator sequence included 5 bp and the stop codon of the *PDR5* ORF. The plasmids were called pABC3-blaster, pABC3-GFP3-blaster and pABC3-mRFP-blaster (**Table 1**), respectively. The integration of the *Pac*I/*Not*I ORF containing *Asc*I-transformation cassettes derived from any of these pABC3-blaster plasmids into the genomic *PDR5* locus of AD∆∆ cells creates direct repeats of the 196 bp *PDR5* terminator between the *URA3* selection marker. The removal of the *URA3* marker can be achieved at high frequency (10^-4^) by plating ~10^6^ logarithmic phase cells onto 5-FOA containing CSM agar plates and isolating single colonies after 2-3 d growth on those plates. Only cells that have lost the *URA3* marker can grow on these plates because the lack of the Ura3 enzyme prevents the incorporation of toxic 5-FOA molecules into the DNA of these cells. After excision via a homologous recombination event between the two repeats, only one copy of the repeat sequence remains. ORFs cloned as *Pac*I/*Not*I fragments into pABC3-GFP3-blaster and pABC3-mRFP-blaster have either a C-terminal green fluorescent protein (yEGFP3) or a monomeric red fluorescent protein (mRFP) tag for multicolour visualisation^15^ of the heterologous proteins in the AD∆∆ host. Sequences of plasmids pABC3-blaster, pABC3-GFP3-blaster and pABC3-mRFP-blaster were submitted to GenBank and are accessible via accession numbers PQ407573, PQ407574 and PQ407575, respectively.

# S6 Creation of pABC3XL

pABC3XL (**Fig. 1a**) is a pABC3-XLmGFPHis^4^ derivative plasmid. pABC3XL was created by fusing three PCR fragments and cloning the resulting 2.2 kb *Pst*I/*Bam*HI digested and gel purified fusion PCR fragment into the gel purified 4.2 kb *Pst*I/*Bam*HI fragment of pABC3. Fragments A (339 bp; primer pair pABC3-up-F/pABC3-PmeI-R) and B (1737 bp; pABC3-PmeI-F/pABC3-PDR5TGFPHIS-SacI-R) were PCR amplified using pABC3-XLmGFPHis and fragment C (206 bp; pABC3-PDR5T-SacI-F/pABC3-PDR5T-BamHI-R) using pABC3-GFP3-blaster as DNA templates. The Phusion High-Fidelity DNA Polymerase (NEB, MA, USA) was used for PCR amplification on a C1000 Touch™ thermal cycler (Bio-Rad, CA, USA). The thermal cycling conditions were 35 cycles denaturation at 98 °C for 10 s, annealing at 65 °C for 10 s and extension at 72 °C for 5 s (fragments A and C) or annealing at 55 °C for 10 s and extension at 72 ˚C for 40 s (fragment B). All three fragments were column purified, mixed at equimolar amounts, and using primers pABC3-up-F and pABC3-PDR5T-BamHI-R fused by PCR using 35 cycles of denaturation at 98 °C for 10 s, annealing at 65 °C for 10 s and extension at 72 °C for 1 min. The resulting fusion PCR fragment was cloned as a *Pst*I-*Bam*HI digested DNA fragment into the unique *Pst*I-*Bam*HI sites to create the interim plasmid pABC3-XLmGFPHis-blaster. The last cloning step for the creation of pABC3XL required the replacement of the 262 bp *PDR5* ‘downstream region’ between the *Eco*RI and *Asc*I-*Xho*I sites (from 3914 to 4175) of pABC3-XLmGFPHis with another 196 bp *PDR5* terminator fragment but this one flanked by *Eco*RI and *Asc*I-*Xho*I sites and cloned as an *Eco*RI-*Xho*I digested DNA fragment into the unique *Eco*RI-*Xho*I sites of the interim pABC3-XLmGFPHis-blaster plasmid created in the previous step. This modification created a ‘true’ *URA3*-blaster cassette that can also be used as a recyclable *URA3* marker anywhere else in the yeast genome. Plasmids pABC3-, pABC3-GFP3-, pABC3-mRFP- and pABC3-XLmGFPHis-blaster do not contain ‘true’ *URA3* blaster cassettes because they only create a repeat flanking the *URA3* marker ‘after’ integration of the linear *Asc*I-transformation cassettes into the genomic *PDR5* locus. Oligonucleotide primers used for PCR amplification and fusion PCR are listed in **Table S2**. All plasmids were confirmed by DNA sequencing. A map of pABC3XL is provided in **Fig. 1a**. The GenBank accession numbers of pABC3-XLmGFPHis, renamed pABC3-XmGH, and pABC3XL are PQ407576 and PQ407577, respectively.

# S7 Plasmid pABC3XL and a method for the creation of tandem gene-arrays stably integrated into the genomic *PDR5* locus

Key features of the improved pABC3XL plasmid are: (i) unique cloning sites between each of the individual modules of the *Asc*I transformation cassette (bold type faced restriction sites; **Fig. 1b**), (ii) a unique 8 bp *Pme*I restriction site in the *PDR5* promoter, and (iii) a ‘true’ *URA3* blaster cassette (**Fig. 1a**). The unique restriction sites between the individual modules allow the easy modification of pABC3XL; e. g. a change in promoter and/or terminator, a change of the repeat sequences or a change of C-terminal tags. And the *URA3*-blaster cassette together with the unique 8 bp *Pme*I restriction site of pABC3XL enable the overexpression of two proteins of interest driven by the same constitutively active *PDR5* promoter stably integrated as tandem-arrays into the genomic *PDR5* locus (**Fig. 1c**). After cloning the first gene of interest as uracil prototrophic transformants into the genomic *PDR5* locus of AD∆∆ cells and the removal of the *URA3* marker, creating AD∆∆-ORF1 (top line; **Fig. 1c**), the unique *Pme*I site can be used to direct integration of the second *ORF2* into the *PDR5* promoter just upstream of *ORF1*. This can be achieved by digesting pABC3XL-ORF2 with *Pme*I and transforming the gel-purified linear DNA fragment into the AD∆∆-ORF1 strain and selecting for uracil prototrophic transformants on CMS-ura plates. Correct transformants containing both ORFs integrated in tandem into the genomic *PDR5* locus can be confirmed by PCR and DNA sequencing. The advantage of this strategy is that two proteins of interest can be overexpressed to the same extent from stably integrated tandem-expression-arrays with no need for expensive selective media to prevent the loss of plasmid(s).

# S8 Heterologous expression of *M. lusitanicus* PDR transporters in *S. cerevisiae* AD∆∆

Six *M. lusitanicus* PDR transporters (*pdr1, pdr3, pdr5, pdr6, pdr7* and *pdr8*) were amplified from a cDNA template that originated from a 10 ng total RNA sample using oligonucleotide primers (**Table S2**) containing either *Pac*I or *Not*I restriction sites at their respective 5’ ends. The Q5 High-Fidelity DNA Polymerase (NEB) was used for PCR amplification with 40 cycles of denaturation at 98 °C for 10 s, annealing at 65 °C for 10 s, and extension at 72 °C for 2.5 min. Gel-purified *Pac*I/*Not*I digested PCR products were used for ligation into the *Pac*I/*Not*I digested pABC3XL plasmid and the ligation products were transformed into *Escherichia* *coli* DH5α (kindly provided by Priv.-Doz. Ingo Bauer, PhD). All plasmids created in this study (**Table 1**) were confirmed by DNA sequencing. *Asc*I digestion of these plasmid constructs released the ~8 kb transformation cassettes from the plasmid backbone. The transformation cassettes were separated from the plasmid backbone by agarose gel electrophoresis, after which they were gel-purified and ~1 µg each used to transform *S.* *cerevisiae* ADΔΔ cells. Correct integration at the genomic *PDR5* locus was confirmed by colony PCR using oligonucleotide primers PDR5-up and PDR5-down (**Table S2**) that bind just ~ 40 bp upstream and downstream of the integration sites (**Fig. 1b**). Colony PCR amplification was carried out using the KOD One™ PCR Mastermix Blue (TOYOBO Co., Ltd., Osaka, Japan) with 45 cycles of denaturation at 98 °C for 10 s, annealing at 65 °C for 10 s, and extension at 68 °C for 45 s per kb.

# S9 Plasma membrane isolation and quantification of protein expression levels

Recombinant strains were grown overnight in 5 mL YPD media and harvested by centrifugation at 3,000 × g for 5 min at 4 °C. Afterwards, 1 g ice-cold zirconium beads (0.5 mm, BioSpec, Berlin, Germany) and 250 µL homogenization buffer (20 % glycerol, 50 mM Tris, 0.5 mM EDTA, pH 7.5, freshly supplemented with 0.5 mM phenylmethylsulfonylfluoride, PMSF, Roche Diagnostics GmbH, Mannheim, Germany) were added. Cell breakage was performed by vortexing on Vortex-Genie 2 (MoBio, Carlsbad, CA, US) for 15 min at maximum speed at 4 °C. Zirconium beads were washed with 1 mL homogenization buffer and the pellet of unbroken cells and cell debris was removed by centrifugation at 2,000 × g for 10 min, 4.0 °C. The supernatant was transferred into a fresh 1.5 mL microfuge tube and centrifuged for 60 min at 15,000 × g, 4 °C. Subsequently the pellet was washed with 1 mL GTED-20 (like the homogenization buffer but with 10 mM Tris) and centrifuged at 15,000 × g for 60 min, 4 °C. The resulting crude plasma membrane pellet was re-suspended in 100 µL GTED-20 and the protein concentration measured using the Lowry Method (DC™ Protein Assay Kit II, Bio-Rad, Feldkirchen, Germany) following the manufacturer’s instructions. A total of 10 µg plasma membrane protein was separated on an 8 % SDS PAGE as previously described.^16^ Green fluorescent signal was measured with ChemiDoc Imaging System (Bio-Rad) and analysed with the corresponding Image Lab Software before staining proteins with Coomassie blue R250 (Bio-Rad) for two hours. De-staining with a 40 % MetOH, 10 % acetic acid solution was performed overnight on an orbital shaker.

# S10 ATPase activities of *M. lusitanicus* PDR transporters

ATPase activities of *M. lusitanicus* PDR transporters were determined from crude plasma membrane preparations as described by Madani *et al*.^17^ Crude plasma membranes were isolated from cells grown in YPD and harvested at mid-logarithmic growth phase of an OD_600_ ~ 1. Two to 5 µg crude plasma membrane proteins in technical duplicates were incubated with and without the ATPase inhibitor vanadate (0.2 – 100 µm, Sigma-Aldrich) in 120 µL assay cocktail (50 mM Tris-HCl pH 7.5, 50 mM potassium nitrate, 0.2 mM ammonium molybdate, 5 mM sodium azide) containing 6 mM Mg-ATP. The ATPase assay was stopped after 60 min incubation at 30 °C by the addition of 130 µL development reagent (1 % SDS, 1.6 % sodium L-ascorbate, 1.2 % ammonium molybdate in 6 M H_2_SO_4_). Phosphate (KH_2_PO_4,_ Sigma-Aldrich) standards for the quantification of the ATPase activities were also included, they ranged from 0 – 100 nmol per well. The phosphomolybdate complex was allowed to develop for 10 min at RT after which the absorbance of the blue Pi-dye complex was measured in Varioskan LUX Multimode Microplate Reader (Thermo Fisher, MA, USA) at 750 nm. The ATPase activities were corrected for the background vanadate-sensitive ATPase activity of crude plasma membranes of the ADΔΔ host strain.

# S11 Structured illumination microscopy

*Saccharomyces* *cerevisiae* strains overexpressing *M.* *lusitanicus* PDR transporters were incubated for 16 h in YPD at 30 °C. The optical cell density (OD_600_) of the overnight culture was adjusted to OD_600_ = 1 with fresh YPD medium and the cells were incubated a further 4 h at 30 °C to reach logarithmic growth. Hundred µL of cells were centrifuged for 3 min at 6,540 × g after which the cells were re-suspended in 100 µL Gibco^TM^ FluoroBrite^TM^ Dulbeccos’s modified eagle medium (Thermo Fisher). An aliquot of this cell suspension was placed onto microscopy slides following the protocol of Pemberton.^18^ The visualisation of GFP fluorescence signals was performed using the Structured Illumination Microscope (SIM) Elyra 7 (Carl Zeiss GmbH, Oberkochen, Germany) with a Plan-Apochromat 63x/1.4 Oil DIC M27 objective, Lens 1.6x. Cell images were processed with the associated ZEISS ZEN core software. Maximal resolution of the images was calculated using the protocol by Gustafsson.^19^

# Supplementary File 2

**Following is the FASTA file of all *Mucor* PDR transporters (manually corrected and experimentally verified sequences highlighted with asterisks) that were used to create the phylogenetic tree presented in Figure 2. *Cryptococcus neoformans* PDR multidrug efflux pump *CneAFR1* was used as the outgroup.**

>CneAFR1

MSAAGVPAELNNLGAPITATTQNPSGLANSQVTSGPVSSATQHDEHRSSAGNTLADEEDDKAVEAEKAEAIDAAGDGKQKRLPADSSEDIVAELEPHHVSVHRGKEEFAALERKYSNLSQRSQHELHRPTTRHSVRSSFSRKDRVVSRLTQDDAEKAKEGEGEFNLVEVLRSGRENQDEAGIKRKAVGVVWEDHEVIGAGGMRINIRNFSSAIIEQFMMPAIKVLGIFGFNPFAPKPKAILHPSSGLLKPGEMCLVLGRPEAGCTTFLKTITNQRAGYMEINGNVEYAGVGWKEMRKRYAGEVVYNQEDDDHLPTLTVAQTIRFALATKTPKKKIPGVSAKQFQDDMLDLLLSMLNIKHTANTIVGNAFVRGVSGGERKRVSIAEMFCSGATVCSWDNSTRGLDASTALDYAKSLRLLTDIMGQTTFVSLYQAGEGIYDQFDKVLVLNEGHVAYFGPAKEARQYMIGLGYRDLPRQTTADYLSGCTDVNERRFADGRDATNVPATPEEMGQAYRESEICARMTREREEYKHLMAEDATARENFKQAVLEQKHKGVGKKSPYTVSFLQQVFIIFKRQLRLKFQDHFGISTGFATSIIIALIVGSVYFRLPETASGAFTRGGLLFLGLLFNALTSFSELPSQMLGRSVLYRQNEYRFYRPAAFALAAVLADVPYNASVIFLFSIVLYFMGGLYSSGGAFFMFFLFVFLTFMVMSAFFRTLGVATSDYNVAARLASVLISFMVTYTGYMIPVQRMKRWLFWIFYLNPLSYGYEAIFANEFSRISLTCDSSYTIPRNIPEAGITGYPDTLGPNQMCSIFGSTPGDPNVSGSDYMAVGYSYYKAHIWRNFGILLGFFTFFMFLQMLFIEVLEQGAKHFSINVYKKEDKDLKAKNERLAERREAFRAGELEQDLSELKMRPEPFTWEGLSYTVPVPGGHRQLLNDIYGYVKPGSLTALMGASGAGKTTLLDVLASRKNIGVVEGDILMNGRPIGTDFQRGCAYAEQQDTHEWTTTVREALQYSAYLRQPQHVPKQEKDDYVEDIIELLELQELADAMIGFPNYGLSVEARKRVTIGVELAAKPELLLFLDEPTSGLDGQSAYNIVRFLKKLCAAGQKILCTIHQPNALLFQSFDRLLLLQRGGECVYFGDIGPDSKVLIDYLERNGAEVPHDANPAEFMLEAIGAGSRKRIGSDWGEKWRNSPEFAEVKREIQELKAEALAKPIEEKSNRTEYATSFLFQLKTVLHRTNVALWRNADYQWTRLFAHLAIGLIVTLTFLQLDNSVQSLQYRVFAIFFATVLPALILAQIEPQYIMSRMTFNREASSKMYSSTVFALTQLLSEMPYSLGCAVSFFLLLYYGVGFPYASSRAGYFFLMILVTEVYAVTLGQAVAALSPTILIAALFNPFLLVLFSIFCGVTAPPPTLPYFWRKWMWPLDPFTRLISGLVSTVLQDQEVVCKDGEYQVFPAPSGQTCQQWAGAFAEAVGGYINNPDSTGDCQFCQYRSGQAFFVPLEISFSTRWRDFGIFICYVVFNILVLLIAARFLKWQRR

>MlusPDR1

MTSTHHLQGSSGEKGREYDQHSSDGSTQTVYQNGLHEFENHGDAFGEPTANAVNIDAAKDEYVDLKRELSRISRLSTHASKLEEGAAEADEFNLDEFLHDLRKNQNENGHELKNLGLIWKNLTVKGQAADAHTIPTVFTFLQFWKFFGVGVSKNKKVILNDLTGHCKPGDMLLVLGRPGAGCTSFLKVMANMRGAYTDVEGTVSYGGIDAETFAKRYRGQVCYNEEEDQHYPTLTAKQTLQFALRMKTPGKRLPDESKTDFVNKVLYMLGNMLGLTKQMNTMVGNAWVRGLSGGERKRMSIAEQMTTSSSINCWDCSTRGLDAASALDYVRSLRIMTDVFKKTTVATLYQASNNIFTLFDKVLLLDEGRCLYFGPTAGAKEYFESLGFVCPKRKSIPDFLTGLCNPNEREVIPGWEDVAPKFASDFEQKYLASDVYKQMMAEFAEYEQTVQNENPADVFKKAVDEEHQKRAPKKAPFTASFYQQVKALTIRQYYLNITDLGALISRYGTILIQSLITASCFFKMTQDGTGAFARGGALFFAVLFNSFISQSELMGFLMGRPILEKHKQYALYRPSAFYIAQVVMDIPYAIVQVLLFEICAYFMMGLKLTAGAFFSFFIILFFMNLCMNGFFRFFGASTTSFFLATQVSGVILIAVTNYTGYTIPYNKMHPWLSWIYWINPMTYCYKALLINELKGVEYSCEGAGNAVPYGPGYDDWNYKVCTMAGGKPGNDYVLGDDYLNDYLSYNPSQMWAPDFIVIIAFFLLFTVMTAGAMELVGLSKSGTLTKLYLPGKAPKPRTAEEEDARRRKQADFGKNMESVSAGTTFSWQNIDYSVPIKGGSLQLLNHVSGVVRPGNLTALMGSSGAGKTTLLDVLARRKTIGKVEGRVYLNNEALMTDFERITGYCEQMDVHQPAVTVREALRFSAYLRQPADVPKEEKDAYVETILELLEMEDIGDAQIGVVEFGFGISVEERKRLTIGMELVGKPKLLFLDEPTSGLDAQSSYNIIRFIRKLADSGWPVLCTIHQPSAILFEHFDHLLLLVRGGRTAYHGEIGKDSQIMIDYFESNGGPKCSPDANPAEYILECVGAGTAGKAKADWATIWENSPEAKALTEELEEIHNNSNPNPTRDAKTYATGLGTQFKLVFSRMSLAYWRSPDYNIGRFLNVMLTSLVTGFTFWKLGSSSSDMLNKVFALFGTFIMAMTLIILAQPKFITERQYFRREYASRYYSWMPWAISAVLVELPYIFFYSACFMFGFYWTAGMNPSAESGGYFYIMFSMLVCWAVTLGFVIAAVSESPLMASVINPLVISILILFCGLMQAPSAMPKFWSSWMYWLDPFHYYIEGLAVNELGDLKVQCNEGDLLRFNAPPGQTCGEYTANFFSYGAPGYIANPNATSPEMCGYCTFNSGPEFYESRFDWSASHKWRNFGILCGYFAFNVILFIGLVYVFRKPRR

>MamPDR1

MTSTHHLQESSGEKGRESDRHSSDGSTQTVYQNGLYEYENNGDAFGEPTANAVNIDAAKHEYVDLKRELSRMSRLSTHASKLEEGTAEADEFNLDEFLHDLRKDQNENGHELKNLGLIWKNLTVKGQAADAHTIPTVFTFLQFWKFLGIGVSKNKKVILNDLTGHCKPGEMLLVLGRPGAGCTSFLKVMANMRGAYTDVDGVVSYGGIDAETFAQRYRGQVCYNEEEDQHYPTLTAKQTLQFALRMKTPGKRLPDESKTDFVNKVLYMLGNMLGLTKQMNTMVGNAWVRGLSGGERKRMSIAEQMTTSSSINCWDCSTRGLDAASALDYVRSLRIMTDVFRKTTVATLYQASNNIFTLFDKVLLLDEGRCLYFGPTAGAKEYFESLGFVCPKRKSIPDFLTGLCNPNEREVLPGYENVAPRYAVDFENKYLASDVYKQVMAEFVEYENTVQNENPAAVFKKAVDEEHQKRAPKKAPFTASFYQQVKALTIRQYYLNITDLGALISRYGTILIQSLITASCFFKMTQDGTGAFARGGALFFAVLFNSFISQAELIGFLMGRPILEKHKQYALYRPSAFYLAQVVMDIPYAIVQVLLFEICAYFMMGLKLTAGAFFSFFIILFFMNLCMNGFFRFFGASTTSFFLATQVSGVILIAVTNYTGYTIPYNKMHPWLSWIYWINPMTYCYKALLINELKGVEYSCEGAGNAVPYGPGYDDWNYKVCTMAGGKPGNNYVLGDDYLNDYLSYNPSQMWAPDFVVVIAFFLLFTVMTAGAMELVGLSKSGTLTKLYLPGKAPKPHTAEEEEARLRKQADFGKNMASVSDGTTFSWQNIDYTVPIKGGSLQLLNHVSGVVRPGNLTALMGSSGAGKTTLLDVLARRKTIGKVEGRVYLNNEALMTDFERITGYCEQMDVHQPAVTVREALRFSAYLRQPADVPKEEKDAYVETILELLEMEDIGDAQIGVVEFGFGISVEERKRLTIGMELVGKPKLLFLDEPTSGLDAQSSYNIIRFIRKLADSGWPVLCTIHQPSAILFEHFDHLLLLVRGGRTAYHGEIGQDSQIMIDYFESNGGPKCSPDANPAEYILECVGAGTSGKAKADWATIWENSPEAKALTEELEEIHNGSNPNPTRDAQTYATSLGTQFKLVFSRMSLAYWRSPDYNIGRFLNVMLTSLVTGFTFWKMGSSSSDMLNKVFALFGTFIMAMTLIILAQPKFITERQYFRREYASRYYSWLPWAISAVLVELPYIFFYSACFMFGFYWTAGMNPSAESGGYFYIMFSMLVCWAVTLGFVIAAVSESPLMAAVINPLVISILILFCGLMQAPSAMPKFWSSWMYWLDPFHYYIEGLAVNELGDLKVECNEGDLLRFSTPPGQTCGEYTANFFSYGAPGYIANPNATSPEMCGYCTFSSGPEFYESRFDWSASHKWRNFGILVSYFAFNIILFIGLVYVFRKPRR

>McicPDR1

MTSTHHLQGSIGEKGREYDRHSSDGSTQTVYQNGLHDYESNGDAFGEPTANAVNIDAAKDEYVDLKRELSRISRLSTHASKLEEGAAEADEFNLDEYLHDLRKDQNDNGHELKNLALIWKNLTVKGQAADAHTIPTVFTFLQFWKFLGIGVSKNKKVILNDLTGHCKPGEMLLVLGRPGAGCTTFLKVMANMRGSYTDVDGTVSYGGIDAETFAKRYRGQVCYNEEEDQHYPTLTAKQTLQFALRMKTPGKRLPDESKTDFVNKVLYMLGNMLGLTKQMNTMVGNAWVRGLSGGERKRMSIAEQMTTSSSINCWDCSTRGLDAASALDYVRSLRIMTDVFKKTTVATLYQASNNIFTLFDKVLLLDEGRCLYFGPTAGAKEYFESLGFVCPKRKSIPDFLTGLCNPNEREVIPGWESVAPKYAADFEEKYLVSDVYKQMMAEFTEYENTVQNENPADVFRKAVDEEHQKRAPKKAPFTASFYQQVKALTIRQYYLNITDLGALISRYGTILIQSLITASCFFKMSEDGAGAFGRGGALFFAVLFNSFISQAELMSFLMGRPILEKHKQYALYRPSAFYIAQVVMDIPYAIVQVLLFEICAYFMMGLKLTAGAFFSFFVILFFMNLCMNGFFRFFGASTSSFFLATQVSGVILIAVTNYTGYTIPYNKMHPWLSWIYWINPMTYCYKALLINELKDVEYSCEGAGNAIPYGPGYDDWNYKVCTMAGGKPGNNYVLGDDYLNDYLSYNPSQMWAPDFIVIIAFFLLFTVMTAGAMELVGLSKSGTLTKLYLPGKAPKPRTAEEEDARRRKQADFNENMDSISTGTTFSWQNIDYTVPVKGGDLQLLNHVSGVVRPGHLTALMGSSGAGKTTLLDVLARRKTIGKVEGRVYLNNEALMTDFERITGYCEQMDVHQPAVTVREALRFSAYLRQPADVPKEEKDKYVETILELLEMEDIGDAQIGVVEFGFGISVEERKRLTIGMELVGKPKLLFLDEPTSGLDAQSSYNIIRFIRKLADSGWPVLCTIHQPSAILFEHFDHLLLLVRGGKTAYHGEIGKDSQTMINYFESNGGPKCSPDANPAEYILECVGAGTAGKAKADWATIWENSPEAKALTEELEEIHNSSNPNPTRDAQTYATSLGTQFKLVFSRMSLAYWRSPDYNIGRFLNVMLTSLVTGFTFWKIGSSSSDMLNKVFALFGTFIMAMTLIILAQPKFITERQYFRREYASRYYSWLPWAISAVLVELPYIFFYSACFMFGFYWTADMNPSSESAGYFYIMFSMLVCWAVTLGFVIAAVSESPLMAAVINPLVISILILFCGLMQAPSAMPKFWSSWMYWLDPFHYYIEGLAVNELADLKVDCNEGDLLRFSAPPGQTCGEYTANFFSYGAPGYIANPNATSPEMCGYCTFNSGPEFYESRFDWSASHKWRNFGIMVSYFAFNIILFIGLVYVFRKPRR

>MracPDR1

MTSTKYLHGGEKGRESDQHSSDGSTQTVYQNGLHEYESHGDAFGEPTANAVDIDAAKDEYVDLKRELSRISRLSTHASKLEEGAAVSDEFNLDEYLNDLRKDEDNNGHELKNLGLIWKNLTVQGQAADAHTIPTLFTFLQFWKFFGVGVSKNKKVILNDLTGHCKEGEMLLVLGRPGAGCTSFLKVMANMRGAYTDVDGTVSYGGIDAGTFAKRYRGQVCYNEEEDQHYPTLTAKQTLQFALRMKTPGKRLPDESKTEFVNKVLYMLGNMLGLTKQMNTMVGNAWVRGLSGGERKRMSIAEQMTTASSINCWDCSTRGLDAASALDYVRSLRIMTDVFKKTTIATLYQASNNIFTLFDKVLLLDEGRCLYFGPTAGAKEYFESLGFHCPKRKSIPDFLTGLCNPNEREVRPGFEDVAPRFAIDFEKRYLESQIHKQVMAEFTEYESMIQNEKPGDVFAKAVDEEHQKRAPKKSPFTASFYQQVKALTVRQYYLNITDLGALISRYGTILIQSLITASCFFKMGSDGAGAFSRGGALFFALLFNSFISQAELVNFLMGRPILEKHKQYSLYRPSAFYVAQVVMDIPYAVVQVLLFEICAYFMMGLKLTAGAFFSFFIILFFMNMCMNGFFRFFGASTSSFFLATQVSGVILIAVTTYTGYTIPYNKMHPWLSWIYWINPLTYCYKALLINELKGVEYSCEGAGNAVPYGPGYDDWNYKVCTMAGGKPGASYVLGDDYLNDYLSYNPEQMWAPDFIVIIGFFLLFTVMTAGAMELVGLSKSGTLTKLYLPGKAPKPRTPEEEDARRKKQANFGQNMDSVSTGTTFSWQNIDYTVPVKGGDLQLLNHVSGVVRPGHLTALMGSSGAGKTTLLDVLARRKTIGKVDGRVYLNNEALMTDFERITGYCEQMDVHQPAVTVREALRFSAYLRQPADVPKEEKDSYVETILELLEMEDIGDAQIGQVEYGFGISVEERKRLTIGMELVGKPKLLFLDEPTSGLDAQSSYNIIRFIRKLADSGWPVLCTIHQPSAILFEHFDHLLLLVRGGRTAYHGEIGQDSQIMIDYFESNGGPKCSPDANPAEYILECVGAGTAGKAKADWATIWENSPEAKSLTEELEEIHGASDVNPTRDAQTYATGLGTQFKLVYKRMALAYWRSPDYNIGRFLNVMFNALVTGFTFWKLGSSSSDMMNKAFALFGTFIMAMTLIILAQPKFITERQYFRREYASRYYSWLPWAISAVLVELPYIFFYSACFMFGFYWTAGMNASSESAGYFYIMFSMLVCWAVTLGFVIAAVSESPLMAAVINPLVISILILFCGLMQAPSQMPKFWSSWMYWVDPFHYYIEGLVVNELGNLKVNCDDGDLLKFNVPAGQTCGEYTANFFSYGSPGYIANPNATSPEMCGYCTFSSGPEFYETRFDWSADHKWRNFGILVAFFIFNIFVFIGLVYVFRKPRR

>MendPDR1

MTSNNYLPPSGEKSRDSDRHSSDGSTQTIYQNGLNDYDPNEGGFGEPTANAVNIAAAKDEYSEVKRELSRISRISTQASKLEEGNAESEEFNLDEFLNGLRRDQTSAGHQIKNLGLIWKDLTVKGQAADAHTIPTVFSYLQFWKFFGLGVSKNKKIILNNLTGHCKEGEMLLVLGRPGAGCTSFLKVMANMRGSYTDVDGLVSYGGIDAKTFAKHYRGQVCYNEEEDQHYPTLTAKQTLEFALRMKTPGKRLPDESKTDFVNKVLYMLGNMLGLTKQMNTMVGNAWVRGLSGGERKRMSIAEQMTTSSSINCWDCSTRGLDAASALDYVRSLRIMTDVFKKTTVATLYQASNNIFTLFDKVLLLDEGRCLYFGPVENAMEYFESLGFHCPNRKSIPDFLTGLCNPNEREIKPGFESTAPQFSADFEKRYLESNIYKQVMSEFQEYQSLVERENPADIFKQAVDEEHQKHAKKSAPYTASFYQQVKYLTIRQYHLNITDMGALISRYGTILIQALITASCFFKMADDGAGAFSRGGALFFCLLFNAFISQSELVNFLMGRPILEKHKQYALYRPSAFYIAQVVMDIPYAIVQVLLFEICAYFMMGLKMTAGAFFSFFIIIFFLNMCMNGFFRFFGASTSSFFLATQVSGVILIATTTYTGYTIPYNKMHPWLSWIYWINPLTYSYKALLINELGGQQYSCEGMGNSVPFGPGYDNWDYKVCTMAGGKPGANYVNGDDYLNDYLSYVPEQMWAPDFIVIIGFFLLFTLMTALAMEFVGLSKSGTLTKLFLPGKAPKPRTAEEEEARRRKQAHISNDMDSVSTGTTFSWQNIDYTVPVKGGNLQLLNSVSGVVRPGHLTALMGSSGAGKTTLLDVLARRKTIGKVDGRVYLNNEALMCDFERITGYCEQMDVHQPAVTVREALRFSAYLRQPSDVPKEEKDKYVETILDLLEMEDIGDCQIGQVETGYGISVEERKRLTIGMELVGKPQLLFLDEPTSGLDAQSSYNIIRFIRKLADSGWPVLCTIHQPSAVLFEHFDHLLLLVRGGRTAYYGEIGRDSKTMIDYFERNGGPKCSPDANPAEYILECVGAGTAGKAKADWADLWEKSAESKALTQELDEIHNSSNANPTRHAQTYATSLATQFKLVHNRMALAYWRSPDYNVGRFLNVMFTALVTGFTFWKLGSSSSDMLNKLFALFGTFIMAMTLIILAQPKFITERMYFRREYASRYYSWLPWGISAVLVELPYIFFFSACFMFGFYWTAGMNSSSEAAGYFYITFSMLVCWAVTLGFVIAAVSESPLMAAVINPLIMSLLILFAGLMQAPSQMPKFWSSWMYWLDPFHYYIEGLAVNELADLEVKCTQGDLLRFNAPPGMTCGEYTKNYFIYGAPGYIDNPDATAPEMCGYCTFKSGPEFYGSRFEWSASHKWRNFGILVAYFAFNVIVFLGLVYLRRKPRR

>MlanPDR1

MTSNSFLPSRGDKSREFDHHSSDGSTQTIYGNALQDDANGNDNSYGEPDANAVDILAAKGEYSELKRELSRISRISTHASQLEEGTAAADEFNLDEFLNGLRQDQNTAGHELKNLGLIWKNLTVKGQAADAHTIPTVFTFLQFWKLFGLGVSKNEKVILNDITGHCKEGEMLLVLGRPGAGCTTFLKVMANMRGSYTSVDGTVSYGGIDSETFAKRYRGQVCYNEEEDQHYPTLTAKQTLQFALRMKTPGTRLPDESKADFVNKVLYMLGNMLGLTKQMNTMVGNAWVRGLSGGERKRMSIAEQMTTSSSINCWDCSTRGLDAASALDYVRSLRIMTDVFKKTTIATLYQASNSIFSLFDKVLLLDEGKCLYFGPIEGAKEYFESLGFHCPNRKSIPDFLTGICNPNEREIKPGFEHSAPKFAVDFEKRYLESQVYKQTMADFTEYEALIESEKPGNSFRKAVDEEHQKRANKNTPFTASFYQQVKALTIRQYYLNITDLGALISRYGTILIQSLITASCFFKMVADGSGAFSRGGALFFCVLFNSFISQSELINFLMGRPILEKHKQYALYRPSAFYIAQVVMDVPYAIVQVLLFEICAYFMMGLKLTAGAFFSFFIILFFMNLCMNGFFRFFGASTSNFFLATQISGIMLISITNYTGYTIPYNKMHPWLSWIYWINPMTYCYKALLINELGGEEYACEGPGNSVPFGPGYDDWNYRVCTMTGGKPGENFVRGDDYLNEYLSYNPDHMWAPDFIAVVGFFILFTFMTATAMELVGLSKSGTLTKLYLPGKSPKPRTPEEEEARRRRQANVNNEMDNVSDGTTFSWQNIDYTVPIKGGELQLLNHVSGVVRPGHLTALMGSSGAGKTTLLDVLARRKTIGKIDGRVYLNNEALMCDFERITGYCEQMDVHQPAVTVREALRFSAYLRQPSDVPKEEKDKYVETILELLEMEDIGDAQIGQVERGFGISVEERKRLTIGMELVGKPKLLFLDEPTSGLDAQSSYNIIRFIRKLADSGWPVLCTIHQPSAILFEHFDHLLLLVRGGRTAYHGEIGKDSQIMIDYFEKNGGPKCSPEANPAEYILECVGAGTAGKAKADWANIWEHSAESKALEAELEEIHSSTNANPTRPAHTYATSLSTQFKLVYKRMALAYWRSPDYNFGRFLNVMVTALITGFTFWKLGTSSSDMNNKLFALFGTFIMAMTLIILAQPKFMTERMFFRREYASRYYSWLPWGISAVLVELPYIFFFSACFMFGFYWTAGMDGASEASGYFYITFTVLVCWAVTLGFVIASVSESPLMAAVINPLIMSMLILFAGLMQAPSQMPKFWSSWMYWLDPFHYYIEGLAVNELEHLTVVCTDNDLLKFSPPPGQTCGEYTANFFTYGAPGYIANPNATQPDLCGYCTFKSGPEFYSSRFQWDAAHKWRNFGILVAYFVFNVIIFLSLVYLRRKPRR

>MlusPDR2

MDMPSAIEGIRANYPNRDDRYDQRQQLRNENYPTSNLNTNEPAHHPDDDSISYDLERSETRRTEGGYGETEGNAVDIDGAIERYQSIRREFTAQSRRKSSIAAASAPAAAAADDVEKGEQQDFDLTEYLTEQHSQIMAAGLKPKNMGVIWKNLTVQGLGADAKVISTNWTWFTSFIQFWKWGKHQGTDFTILKDNNGFCKAGEMLLVLGRPGAGCTSLLRVLANMRASYTNIEGEVTYGGIDAEEFGKHFRGEVCYNEEEDLHYPTLTTQQTLRFALKNKTPGKRLPGESKAEFIDKLLYMLGNMLGLTKQMNTMVGNAFVRGLSGGERKRLSIAEQMTTRSSINCWDCATRGLDASSALDYVRSLRIMTDILGKTTISTLYQASDSIFHLFDKVMVLDEGRCIYFGPTSTAKQFFIDMGFYCPDRKSTPDFLTGLCNMNEREYREGFEGKVPINAVQFEKVYQESPLFAKMMQERDEYEQKINQDRPAETFREAFSDAHQKHAPKHSPFVATYLEQVKALTVRQFQLILGDKGALVSRYGGVVVKGLIMASVFYMMPQDASGAFSRGGAFLFSLLFNALIAQSELAAFMQGRRVLEKHKHFALYHPSAFYIATVIADIPLALIQVIIFELCVYFMMGLQLEAGRFFTFFIVLVMTNLCMNGFFRFWGAVSPNFFTASQLSSILLIAALIYCGYQIPYTQMHPWLFWIYWINPLAYGYKALISDEMRNLHFSCEGANSVPYGPTYTDQQYKTCILPGAAPGASYILGDDYLAVNYGYYVWQRWINFVAVVLFFFFFTILTALAMEYVDLQKEGSITKVYKKGCAPEADSDEKLMQQVTTVNDQEMEAVTDGTTFSWYDLHYCVPVKGGTRELLNGVGGIVKPGHLCALMGSSGAGKTTLLDVLAKRKTIGKVEGNIYLNGEALAADFERITGYCEQMDVHNPNATVREALRFSAYLRQPQEVSKEEKDEYVEQILGLMEMQKIGDALIGDLEAGIGISVEERKRLTIATELVGKPKLLFLDEPTSGLDAQSSYNIVRFIRKLADAGWPVLCTIHQPSATLFEHFDHLLLLMRGGRTAYFGEIGKDSRTMIDYFESNGGPKCSPQANPAEYILECVGAGTAGKSTQDWADVWAGSSQAKALEQELEHIHSTVDHSASRKVNTYALPFWEQLKLVYMRMNVSWWRCPTYNMGRLFNVCFIGLISGFSFWKLGSSPADMQNRMFSVFTTLLMSNALIILAQPRFMQERMWFRREYASKYYGWAPFALSCVLVEIPYLIVLGTIFLFCFYWTAGLQNESDRIGFFYIHFIMFLFYSVSLGFTIASFSATPPMAAVINPFFTSILILFAGIMQPPSAMPHFWSAWMYWLDPYHYLIEGLVVNVMDGVEVVCGEGDYLILNAPPGQTCGEYMQEFFNNGGPGYLGNPDATGSCNYCQYKTGNDFYEERIGWHYSHRWRNFAILCAYTVFNVMLFMFFVFLFRKQKR

>MambPDR2

MDMPSANEGIRADYPNRDDRYDQRQQIRHENNQVSHLSANEHTHHLDADNISYVDMERSDTRRTEGGYGETEGNAVDIEGAIERYQSIRRELTAQSRRKSSIAAISASSAAAAAAAASAGADVEKGEQQDFDLTEYLTEQHSQIMAAGLKPKNMGVIWKNLTVQGLGADAKVIPTNWTWIASFVQFWKWGKHKGTDFTILKDNNGFCKAGEMLLVLGRPGAGCTSLLRVLANMRASYTSIEGDVTYGGIDAEEFGKHFRGEVCYNEEEDLHYPTLTTQQTLRFALKNKTPGKRLPGESKAEFIDKLLYMLGNMLGLTKQMNTMVGNAFVRGLSGGERKRLSIAEQMTTRSSINCWDCATRGLDASSALDYVRSLRIMTDILNKTTISTLYQASDSIFHLFDKVMVLDEGRCIYFGPTSTAKEFFTDMGFYCPDRKSTPDFLTGLCNMNEREYREGFEGKVPVNAVQFEKVYEESALFAKMMQERSEYEQTINQDRPAETFREAFSDAHQKHAPKRSPFVATYLEQVKALTVRQFQLILGDKGALVSRYGGVVVKGLIMASVFFMMPQDASGAFSRGGAFLFSLLFNALIAQSELAAFMQGRRVLEKHKHFALYHPSAFYIATVIADIPLALIQVIIFEICVYFMMGLQLEAGRFFTFFIVLVMTNLCMNGFFRFWGAVSPNFFTASQLSSILLIAALIYCGYQIPYTQMHPWLFWIYWINPLAYGYKALISDEMRNLKFSCEGTSSVPYGPTYTEQQYKTCILPGAAPGASYILGDDYLAFNYGYYVWQRWINFVAVVLFFFFFTILTALAMEYVDLQKEGSITKVYKKGCAPEADSDEKLMQQVTTVNDQEMEAVTDGTTFSWHHIDYRVPVKGGTRQLLNDVAGIVKPGHLCALMGSSGAGKTTLLDVLAKRKTIGKIEGNIYLNGEALADDFERITGYCEQMDVHNPNATVREALQFSAYLRQPADVSKEEKDAYVEQIIVLMEMQKIADALIGDLEAGVGISVEERKRLTIATELVGKPKLLFLDEPTSGLDAQSSYNIVRFIRKLADAGWPVLCTIHQPSATLFEHFDHLLLLMRGGRTAYFGEIGKDSRTMIDYFESNGGPKCSPQANPAEYILECVGAGTAGKSTQDWADVWAGSPQAESLEQELEHIHSTADHTASRKATAYALPFWEQLKLVYMRMNVSWWRCPTYNMGRLFNVCFIGLISGFSFWKLGSSPADMQNRMFSVFTTLLMSNALIILAQPRFMQERMWFRREYASKYYGWAPFALSCVLVEIPYLIVLGTIFLFCFYWTAGLQNESDRIGFFYIHFIMFLFYSVSLGFTIASFSATPPMAAVINPFFTSILILFAGIMQPPSAMPHFWSAWMYWLDPYHYLIEGLVVNVMDGVEVVCGEGDYLVLNAPPGQTCGEYMQEFFNSGGAGYLGNPDATGSCNYCQYTTGNDFYEERIGWNFSNRWRNFGILSAYTVFNVLLFMFFVFLFRKQKR

>McicPDR2

MDMPSANEGIRADYPNRDDRYAQRQQIRNENNPATDLSMNEPASNLDADSISYVNVERSDTRRTEGGYGETEGNAVDIDGAIERYQSIRRELTAQSRRKSSIAASTAAAANAANADVEKGEQQDFDLTEYLTEQHSQIMAAGLKPKNMGVIWKNLTVQGLGADAKVISTNWTWITSFVQFWKWGKHKGTDFTILKDNNGFCKAGEMLLVLGRPGAGCTSLLRVLANMRASYTSIDGEVSYGGIDAEEFGKHFRGEVCYNEEEDLHYPTLTTQQTLRFALKNKTPGKRLPGESKSEFIDKLLYMLGNMLGLTKQMNTMVGNAFVRGLSGGERKRLSIAEQMTTRSSINCWDCATRGLDASSALDYVRSLRIMTDILNKTTISTLYQASDSIFHLFDKVMVLDEGRCIYFGPTSTAKDFFVDMGFYCPDRKSTPDFLTGLCNMNEREYREGYEGNVPLNSLQFEKVYRESTLYAKMMQERDEYEQKINQDRPAETFREAFSDAHQKHAPKHSPFVATYLEQVKALTVRQFQLILGDKGALVSRYGGVVVKGLIMASVFFMMPQDATGAFSRGGAFLFSLLFNALIAQSELAAFMQGRRVLEKHKHFALYHPSAFYIATVIADIPLALIQVIIFEICVYFMMGLQLEAGRFFTFFIVLVMTNLCMNGFFRFWGAVSPNFFTASQLSSILLIAALIYCGYQIPYTQMHPWLFWIYWINPLAYGYKALISDEMRNLHFSCEGANSVPYGPTYTDQQYKSCILPGAAPGASYILGDDYLAVNYGYYVWQRWINFVAVVLFFFFFTILTALAMEYVDLQKEGSITKVYKKGCAPEADSDEKLMQQVTTVNDQEMEAVTDGTTFSWHHIDYTVPVKGGTRQLLNDVAGIVKPGNLTALMGSSGAGKTTLLDVLAKRKTIGKIEGNIYLNGEALADDFERITGYCEQMDVHNPNATVREALRFSAYLRQPQEVSKEEKDDYVEQIIVLMEMQKIADALIGDLEAGIGISVEERKRLTIATELVGKPKLLFLDEPTSGLDAQSSYNIVRFIRKLADAGWPVLCTIHQPSATLFEHFDHLLLLMRGGRTAYFGEIGKDSRTMIDYFESNGGPKCSPQANPAEYILECVGAGTAGKSTKDWADVWAGSPQAEALEQELEHIHSTVDHTATRKVTTYALPFWEQLKLVYMRMNVSWWRCPTYNMGRLFNVCFIGLISGFSFWKLGNTPADMQNRMFSVFTTLLMSNALIILAQPRFMQERMWFRREYASKYYGWAPFALSCVLVEIPYLIVLGTIFLFCFYWTAGLQNESDRVGFFYIHFIMFLFYSVSLGFTIASFSATPPMAAVINPFFTSILILFAGIMQPPSAMPHFWSAWMYWLDPYHYLIEGLVVNVMDGVEVVCGEGDYLVINAPPGQTCGEYMQEFFNNGGPGYLGNPDATGSCNYCQYKTGNDFYEERIGWNFSNRWRNFGILCAYTVFNVMLFMFFVFLFRKQKR

>MracPDR2

MDMPSANDGLRAEYPNRDDRYDQRQKIRNENNAPSENIDADSTSYIDVERSDTRRTEGGYGETEGNAVDIDSAIERYQSVRREFTAQSHRKSSIAAASAAAAAAAAAVDAEKGDQQDFDLTEYLTEQHSQIMAAGLKPKNMGVIWKDLVVQGLGADAKVISTNWTWIASALQFWKWGKHKGTDFTILKDSNGFCKAGEMLLVLGRPGAGCTSLLRVLANMRASYTSIEGEVTYGGIDAEEFGKYFRGEVCYNEEEDLHYPTLTTQQTLRFALKNKTPGKRLPDESKNDFIDKLLYMLGNMLGLTKQMNTMVGNAFVRGLSGGERKRLSIAEQMTTRSSINCWDCATRGLDASSALDYVRSLRIMTDILNKTTISTLYQASDSIFHLFDKVMVLDEGRCIYFGPTSTAKDFFTDMGFYCPDRKSTPDFLTGLCNMNEREYREGYEGKVPLNSLQFEKVYQESALNAKMMQERDEYEQRINQDRPAETFREAFSDAHQKHAPKHSPFVATYLEQVKALTVRQFQLILGDKGALVSRYGGVVVKGLIMASVFFMMPQDATGAFSRGGAFLFCLLFNALIAQSELAAFMQGRRVLEKHKHFALYHPSAFYIATVIADIPLALIQVIIFEICVYFMMGLQLEAGRFFTFFIVLVMTNLCMNGFFRFWGAVSPNFFTASQLSSILLIAALIYCGYQIPYTQMHPWLFWIYWINPLAYGYKALISDEMRNLHFSCEGTNSVPYGPTYTDQQYKSCILAGAAPGASYILGDDYLAVNYGYYVWQRWINFVAVVLFFFFFTILTALAMEYIDLAKEGSITKVYKKGCAPEAESDEKLMQQATTVNDQEMEAVTDGTTFSWHHIDYTVPVKGGTRQLLNNVAGIVKPGNLTALMGSSGAGKTTLLDVLAKRKTIGTIEGNIYLNGETLANDFERITGYCEQMDVHNPNATVREALRFSAYLRQPREVPKEEKDEYVEQIIILMEMEKIADALIGDLEAGIGISVEERKRLTIATELVGKPKLLFLDEPTSGLDAQSSYNIVRFIRKLADAGWPVLCTIHQPSATLFEHFDHLLLLMRGGRTAYFGEIGKDSRTMIDYFEANGGPKCSPQANPAEYILECVGAGTAGKSTQDWADIWAVSPEAQTLEQELEQIHSSVNHTSSRKVTTYALPFWEQLKLVYMRMNVSWWRCPTYNMGRLFNVCFIGLISGFSFWKLGSSPPDMQNRMFSVFTTLLMSNALIILAQPRFMQERMWFRREYASKYYSWAPFALSCVLVEIPYLIVLGTIFLFCFYWTAGLQNESDRIGFFYIHFIMFLFYSVSLGFTIAAFSATPPMAAVINPFFTSILILFAGIMQPPNAMPHFWSAWMYWLDPYHYLIEGLVVNVMDGVQVICSENDFIKINAPPGQTCADYMQEFFNNGGPGYLGNPDATGSCNYCQYTTGNDFYEERIGWNFNNRWRNFGILTCYTIFNVMLFTFFVFLFRKQKR

>MendPDR2

MDMPAAAPGIGREYPNRDDEYQQRWNAADNTLVGESNYVDEDNTQPANTGGSGYANMERTETRRTEGGFGESEGNAVDINEALERYHSVRREFTQQSRKSKTAATAPETTTAADAEKGDEHDFDLTEYLSDQHSQITEAGLKPKNMGVIWKALVVQGLGADAKVISTNWTWISSFVQFWKWGKHKGTDFTILKGNDGFCKAGEMLLVLGRPGAGCTTLLRVLSNMRASYTKIDGAVSYGGIEAEEFAKHFRGEVCYNEEEDLHYPTLTTQQTLRFALKNKTPGKRLPGESKEEFINKVLYMLGNMLGLTKQMNTMVGNAFVRGLSGGERKRLSIAEQMTTRSSINCWDCSTRGLDASSALDYVRSLRILTDIFEKTTIATLYQASDSIFHLFDKVMVLDEGRCIYFGPTNIAKQFFVNQGFYCPERKSTPDFLTGLCNMNEREVQPGFDGKVPLNAVQFEKVYRESSLSQQMLAERDAYEEQINKDKPAETFREAFSEAHQKHAPRHSPFIATYFEQVKALTVRQFQLILGDKGSLISRYGGVVVKGLIMASVFYMMPRDASGAFSRGGSFLFSLLFNALIAQSELAAFMQGRRVLEKHKHFALYHPSAFYIATVISDIPLAIIQVIIFEICVYFMMGLVLEAGRFFTFFIILVMTNMCMNGFFRFWGAVSPNFFTASQLSSVLLIACLIYCGYQIPYVLMHPWLFWIYWINPLAYGYKALISSELRDLQFTCIGVGNSVPNGPAYNNVDQAYKSCILPGGQPGASYVLGDDYLAVAYGYYTWQLWINFVAVVLFFLFFTLLTALAMEYVDLQKEGSITKVYKKGCAPEDVDEKELLHQTNTINDQEMDAVTEGTTFSWHHIDYTVPVKGGTRQLLNDVCGIVKPGNLTALMGSSGAGKTTLLDVLSKRKTIGKIEGNIYLNGEKLAEDFERITGYCEQMDVHNPNATVREALQFSAYLRQPAEVPKAEKDAYVEQIIELMEMQKIADALIGDLEAGIGISVEERKRLTIATELVGKPKLLFLDEPTSGLDAQSSYNIVRFIRKLADAGWPVLCTIHQPSATLFEHFDHLLLLMRGGRTAYFGEIGKDSRIMIDYFESNGGPKCSPNANPAEYILECVGAGTAGKSTQDWADIWADSSQAKTLEEELNGIHGYVDHSQTRNVTTYALPFFQQLKLVYKRMNVSWWRCPTYNMGRLFNVCFIGLISGFSFWKLGNTPADMQNRMFSVFTTLLMSNALIILAQPRFMQERMWFRREYASKYYGWAPFALSCVLVEIPYLIVLGTIFLFCFYWTAGLQNESDRVGYFYIHFLMFLFYSVSLGFTIAAFSATPPMAAVINPFFTSLLILFAGIMQPPSAMPKFWSSWMYWLDPYHYLIEGLVVNVMDGVEVVCGSNDYIKIRPPPGKSCGDYMAPYFQSGGVGYLGNPSASNDFCDYCQYSTGADFYEIRIGWSFANRWRNFGILTGFTIFNVIAFIFFVFLFRKQKR

>MlusPDR3

MIDPIARTYSQGGFGEANGNAVDIQSAFEEYEHVRRTLTERSNAAAATGQKVDIEKGEQVFDLTEYLANQHAQLTEAGLKAKNMGLVWKGLTVKGLGADARSILTNGSVILKMLQFWTWGKQAGSEVTILHDNDGFCKPGEMLIVLGRPNAGTSTLLRVLSNMRAAYTSVQGDVTYGGIDAQEFGKYFKGEVCYNEEEDLHYPTLTTEETLRFALKTKTPATRLPGESKTDFIENLLYMLGNMLGLTKQMKTMVGDAFIRGLSGGERKRLSIAEQMTTHSSINCWDGSTRGLDASSALDYVRSLRIMTDIMHKTTVATLYQASDSIFSLFDKVMVLDEGRCIYFGPIASAKAYFVEMGFYCPERKSTPDFLTGLCNLNEREVRPGFEDRVPLNAAQFEKVYKESSLYAQMMRERDAYEAEISKDEPQVSFREAFQQAHNTPFVVSYYQQVRALTVRQLQLIWGDKTSLIVRYVDIIAKGLITASIFYLMPLTVEGLFSRAGAYIFILFFNAVVAQAELPAFMNARGVLEKHKHFAMYRPSAFYVAQVIADFPLAVLQAILFELCCYFIMGFLLDAGRFFSFFINLVAITMCMNSFFRFWGAICPNFYTASQISSIIFTAFIIYVGYQLPYPYMHPWLMWIYWINPLAYSFKALIGTELTGARFSCDGVNGVPFGPTYTEQAYRSCVLPGAEPGASFVLGDSYLAQYYGYYTSQNWINFLAVVLLFVFFSILAALAMEFIDLKKGSTITKVYKKGFTPKVTTTTDHEKQLEQTESRAHQELEAVSDGTTFTWHALEYTVPIKKGKLKLLNNVGGYVKPGNLTALMGSSGAGKTTLLDVLSRRKTIGTIEGNIYMNGEHLANDFERLTGYCEQMDVHNPNATVREALQFSAYLRQPAHVPKQEKDAYVEQILDLLEMQNIGDALVGDLSEGTGISVEERKRLTIAVELVGKPKLLFLDEPTSGLDAQSSYNIIRFIRKLADAGWPVLCTIHQPSATLFEHFDHLLLLVRGGQTAYFGEIGKDSRTMIDYFESNGGPVCAPEANPAEYILECVGAGTAGKASKDWAQVWSDSPEAATLEKELEAIHSTIDHTVTRQVQTYALPFWQQLALVFGRMNTSWWRSPSYNLGRALTVCLIGFITGFTFWKVGSSPVDLQNRMFGLLTSLFMGNSMIILIQPRFIQERTWFRREYASKYYSTAPFALSCILVEIPYLIAVSALFMFFYYWTTGLQNESDRVGYFFIIFVVYLFHSVSFGYVIAAFCESPTLAAIINPFFLTVLLLFNGIFQPPSSMPAFWSSWMYWLNPYHYLVEGLVTNGLESVQVICEDKDYIKINAPPGSTCGEYMSTYFADGGLGYLGNPNSTDVCNYCQYSTGNEYYEHRIGWSFSNRWRNYGLLWLFTLFNVAVFFAFVYAFRKQKR

>MambPDR3

MNKANYPNRKRSDQSLSNTANTTEDDTDTVIDPISGARSQGGFGELNGNAVDIQGAFEEYEHVRRTLTEQSNAAAASKTDIERGEQAFDLTEYLTTQHAQLTEAGLKAKNMGLVWKHLTVKGLGADSRSILTNGSVILKILQFWKWGRQSGSEVTILHDNDGFCKPGDMMIVLGRPNAGTSTLLRVLSNMRGAYTSVQGDVTYGGIDAQEFGKYFKGEVCYNEEEDLHYPTLTTEQTLRFALKTKTPATRLPGESKSDFIENLLYMLGNMLGLTKQMKTMVGDAFVRGLSGGERKRLSIAEQMTTHSSINCWDGSTRGLDASSALDYVRSLRIMTDIMHKTTVATLYQISDSIFQLFDKVMVLDEGRCIYFGPIASAKTYFVEMGFHCPERKSTPDFLTGLCNLNEREVRPGFEERVPLSAAQFEKVYKESSLYAQMMRERDAYEAEISNDQPKVSFREAFQQAHNTHFVISYYQQVKALTVRQLQLIWGDKTSLLIRYIDVIAKGLITASIFYMMPLTVEGLFSRAGAYIFIVFFNAVVAQAELPAFMEGRGVLEKHKHFAMYHPSAYYVAQVIADIPLTILQAILFELCCYFIMGFLLDAGRFFSFFINLVAITMCMNSFFRFWGSVCPNFYTASQISSIIFTAFIIYVGYQLPYPYMHPWLMWIYWINPLAYSFKALIGTELTGARFSCAGVNAVPFGPTYTEQAYRSCILPGAEPGASFVLGDNYLAQYYSYYTSQNWINFLAVVLLFVFFSILAALAMEFIDFNKGSTTTKVYKKGSTPKVTTAQKQLEQNTSGMHQELDAVNDGTIFTWHNVDYSVPIKGGKLKLLNNVGGIVKPGNLIALMGSSGAGKTTLLDVLSRRKTMGTIEGNIYMNGEHLADDFERLTGYCEQMDVHNPNATVREALQFSAYLRQPADVPKQEKDAYVEQILSLLEMQTIGDALVGELEHGTGISVEERKRLTIAVELVGKPKLLFLDEPTSGLDAQSSYNIIRFIRKLADAGWPVLCTIHQPSATLFEHFDHLLLLVRGGQTAYFGEIGKDSRTMIDYFESNGGPACAPEANPAEYILECVGAGTASKTSKDWAQVWSDSPEAAALEKELDIIHSSTDHTVARQVQAYALPFWHQFSLVFGRMNLSWYRSPSYNLGRALTVCLIGLVTGFTFWKVGSSPVDLQNRMFGLLTSLFMGNSMIILIQPRYIQERAWFRREYASKYYGWSPFALSCILVEIPYLIAMSALFMFFYYWTTGLQNESDRVGYFFIIFVVYLFHSVSFGYVIAAFCESPTLAAIINPFFLTVLLLFNGIFQPPNSMPTFWSSWMYWLNPYHYLVEGLVTNGLDSVQVVCEEKDYIKINTPPGSTCGEYMSQYFADGGLGYLGNPNSTNVCDYCQYSTGNDYYEYRIGWSFSNRWRNFGLLWLFTLFNVAVFFVFVYAFRKQKR

>McicPDR3

MTDKLPISRTYSQGGFGESNGNAVDIQSAYEEYEHVRRTLTELSNAAASHKTDIEKGEQVFDLTEYLANQHAQLTEAGLKAKNMGLVWKHLTVKGLGADARSILTNGSVILKILQFWKWGKQSGSEVTILNDNDGFCKSGEMLLVLGRPNAGTSTLLRVLSNMRAAYTSVQGNVTYGGIDAQEFGKYFKGEVCYNEEEDLHYPTLTTEETLRFALKTKTPATRLPGESKSDFIENLLYMLGNMLGLTKQMKTMVGDAFVRGLSGGERKRLSIAEQMTTHSSINCWDGSTRGLDASSALDYVRSLRIMTDIMHKTTVATLYQASDSIFQLFDKVMVLDEGRCIYFGPIASAKDYFVEMGFYCPDRKSTPDFLTGLCNLNEREVRPGFEARVPLNAAQFEKVYKESGLYRQMINERDAYEAEINTDKPEVSFREAFQQAHNTPFVINYYQQVKALTVRQLQLIWGDKTSLLIRYIDVIAKGLITASIFYMMPLTGEGAFSRGGAYIFIVFFNSVVAQAELPAFMEGRRVLEKHKHFAMYRPSAYYVAQIIADIPLSILQAILFELCCYFMMGFILDAGRFFSFFVNLIAITMCMNSFFRFWGSICPNFYTASQISSIIFTAFIIYVGYQLPYPYMHPWLMWIYWINPLAYSFKAMIGTELTGAHFSCDSVNAVPFGPTYTEQAYRSCILPGAEPGASFVLGDNYLAQYYAYYTSQNWINFLAVVLLFVFFSILTALAMEFVDLKKGSTITKVYKKGFTPKAITAEKQLEQTTSHVNQELEAVNDGTSFTWHHVDYTVPIKGGKLKLLNNVGGYVRPGHLTALMGSSGAGKTTLLDVLSRRKTIGTIEGNIYMNGENLADDFERLTGYCEQMDVHNPNATVREALQFSAYLRQSADVSKEEKDAYVEQILDLLEMQSIGDALVGDLEQGTGISVEERKRLTIAVELVGKPKLLFLDEPTSGLDAQSSYNIVRFIRKLADAGWPVLCTIHQPSATLFEHFDHLLLLVRGGQTAYFGEIGKDSRTMIDYFESNGGPACSPEANPAEYILECVGAGTSGKVSKDWAQVWSESPEAAALEKELEAIHSTTDHTVTRQVQTYALPFWQQLSLVLRRMNQSWWRSPSYNLGRALTVCLIGFVTGFTFWKVGSSPVDLQNRMFGLLTSLFMGNSMIILVQPRYIQERAWFRREYASKYYGWSPFALSCILVEIPYLIAVSALFMFFYYWTTGLQNESERVGYFFIIFVVYLFHSVSLGYVIAAFCESPTLAAIINPFFLTVLLLFNGIFQPPSSMPAFWSSWMYWLNPYHYLVEGLVTNGLEGVQVVCEEKDYIKINTAPGSTCGEYMSQFFTDGGLGYLGNPNSTDVCDYCQYSSGNDYYSYRIGWSFSNRWRNFGLLWLFTLFNVAVFLAFVYAFRKQKR

>MlusPDR4

MAYQNRISNDSTSRSFSSSSNEAATVVDAAAYPQLSQSQSRGGYGESEGNAVDIEVALQEYRSVQRELTRQSKRTQADADVEKAQDNDFDLTEYLANQHEQLTSAGLKSKNMGVIWKNLTVQGLGADARSIATNWSVLAKAAQFWEWAKHKGTDFTILHDNNGFCKSGEMLLVLGRPGAGCSTLLRLLANMRGSYTSIEGDVSYGGIDAHDFGKHFKGEVCYNEEEDLHYPTLTTEQTLRFALKNKTPSTRVPGESKQDFINSVLYLLGNMLGLTKQMKTMVGNAFVRGLSGGERKRLSIAEQMTTHSSINCWDCSTRGLDASSALDYVRSLRIMTDIMQKTTISTLYQASDSIFELFNKVMVLDEGRCIYFGPTSEAKAYFTDMGFYCPPRKSAPDFLTGLCNLNERQVQPEYQDRQVPMNASQFEKAYKESAMYSKMMAERDQYEQEINQDKPYESFREAFKQAHDTPAVVSYYDQVKALTVRQFQLIWGDKYSLFVRYGDVIVKGLITASVFYMMPLNGTGAFSRSGAYLFALIFNAFIAQSELPAFMQGRRVLEKHKHFALYHPSAFYFAQVFVDIPLAIIQAIVFELCIYFLMGLASDAGKFFTLLVNLIAITLCMNGFFRFLGAICPNFFLASQLSSFAFVALLINSGYQLPYPDMHPWFMWIYWINPIAYSYKSILSSELRGAHFSCDGVNGVPNGPSYTDPAYRTCVLPGAKPGASFVLGDDYLAQQYEYYTSQIWINFVAVVLFFVLFTVLTALCMEYVDLKQEGTITKVYKKGASPPVVSAETQLKQQQTREERQLEAVSGGTVFSWHSIKYTVPVKSGTRQLLDNVAGIVKPGHLCALMGSSGAGKTTLLDVLAKRKTIGTIEGNIYMNGEHLASDFERLTGYCEQMDVHNPNTTVREALQFSAYLRQSADVPKEEKDAYVEQILDLLEMQKIGDALVGDLEEGTGISVEERKRLTIAVELVGKPKLLFLDEPTSGLDAQSSYNIIRFIRKLADAGWPVLCTIHQPSATLFEHFDHLLLLMRGGRTAYFGEIGKDSRTMIDYFESNGGPTCSPEANPAEYILECVGAGTAGKVTTDWAQVWSNSPEAAALENELQTIHNSIDHTVNRKVEAYAQSFWSQLFFVFKRMNMSWWRSPSYNVGRLFNVVFIGLITGFTFYKVGNTPKDMQNRMFGLLTSMFMGNTMIILIQPRFMQERTWFRREYAGKFYGWVPFALSCILVEIPYLVFLSAVFLVCYYWTTGLQNVSERVGYFFIQFVVYMFHSITFGYAIAAFCESPTMAAILNPFFTTILYLFSGLFQTPKAMPKFWSSWMYWLDPYHYFIEGLVTNGLDSVPVVCNDSNYIKIKAPPGRNCGDYMADFFADGGLGYIGNPNGTDYCDYCQYSIGNDYYETNIGWSYANRWRNCGLLWVFTVFNVVLFVVFVYLFRKQKR

>MambPDR4

MGYQNRISNDSTSRSFCSSSNEAATVVDAATAAYPDLSQTRSRGGFGEADGNAVDIEIALEEYRSVQRELTRQSKKTQPDADVEKAQDNDFDLTEYLANQHDQLTSAGLKSKNMGVIWKNLTVQGLGADARSIATNWSVLAKAAQFWEWAKHKGTDFTILHDNNGFCKSGEMTLVLGKPGSGCSTLLRVLANMRGSYTSIEGAVSYGGIDAHDFGKHFKGEVCYNEEEDLHYPTLTTEQTLRFALKNKTPSTRVPGESKQDFINSVLYLLGNMLGLTKQMKTMVGNAFVRGLSGGERKRLSIAEQMTTHSSINCWDCSTRGLDASSALDYVRSLRIMTDIMQKTTISTLYQASDSIFDLFDKVMVLDEGRCIYFGPTSEAKTYFTDMGFHCPPRKSTPDFLTGLCNLNERQIQPEFQDHQMPMNAAQFEKHYKESAMFDKMMTERDQYEKEINQDKPYESFREAFKQAHDTPAVVSYYDQVKSLTVRQFQLIWGDKYSVFVRYGDVIVKGLITASVFYMMPLNGTGAFSRAGAFLFAIIFNAFIAQSELPAFMQGRRVLEKHKHFALYHPSAFYFAQVIVDIPLAVLQAIVFEVCIYFMMGLASDAGKFFTLFVNLIAITLCMNGFFRFLGAICPNFFLASQLSSFAFVALLINSGYQLPYPDMHPWFMWIYWINPIAYSYKSILSSELRGALFSCDGVNGVPNGPSYTDSAYRTCILPGAKPGASFVLGDDYLAQQYEYYTSQIWINFVAVVLFFVLFTVLTALCMEYVDLKQEGTITKVYKKGSSPQVLSTEAQLKQQQTREEHELEAVSSGTTFSWHNVNYSVPVKGGTRQLLNGVAGIVKPGNLTALMGSSGAGKTTLLDVLAKRKTIGTIEGNIYMNGEHLASDFERLTGYCEQMDVHNPNTTVREALQFSAYLRQSADVPKEEKDAYVEQILDLLEMQKIGDALVGDLEEGTGISVEERKRLTIAVELVGKPKLLFLDEPTSGLDAQSSYNIIRFIRKLADAGWPVLCTIHQPSSTLFEHFDHLLLLMRGGRTAYFGEIGKDSRTMIDYFESNGGPTCSPEANPAEYILECVGAGTAGKVTTDWAQVWANSPEAAALEEELETIHKSIAHNVNRKVSAYAQSFWSQLYHVYKRASISWWRSPSYNVGRLFNVVFIGLITGFTFWKVGNTPKDMQNRMFGLLTTMFMGNTMIILIQPRFMQERTWFRREYASKYYGWSPFALSCILVEIPYLLSLSAVFLVCYYWTTGLQNVSERVGYFFIQFVVYMFHSVTLGYAIAAFCESPTMAAILNPFFTTILYLFSGLFQTPKAMPKFWSSWMYWLDPYHYFIEGLVTNGLDSVPVVCSDSNYIKIKAPAGKNCGEYMADFFAGGGLGYIGNPNGTDYCDYCQYSIGNDYYETNIGWSYSNRWRNFGLLWVFTAFNVVLFVLFVYLFRKQKR

>MracPDR4

MSNKYQNRISNESSRSFSSSNQAATVVDHYNDLTQTQSRGGYGEIDGNAVDIEEALEEYRSVQRELTHQSKRTNDNADVEKAEATENNFDLTEYLANQHDQLTSAGLKSKNMGVIWKDLNVEGLGADARSIATNWSVLAKATQFWEWAKHKGTDFTILHENNGFCKSGEMLLVLGRPGSGCSTLLRVLANMRASYTSITGDVTYGGIDAYDFGKHFKGEVCYNEEEDLHYPTLTTEQTLRFALKNKTPSTRVPGESKEEFINGVLYMLGNMLGLTKQMKTMVGNAFVRGLSGGERKRLSIAEQMTTHSSINCWDCSTRGLDASSALDYVRSLRIMTDIMQKTTISTLYQASDSIFDLFDKVMVLDEGRCIYFGPTSEAKSYFTDMGFYCPSRKSTPDFLTGLCNLNERQVQPNYQGQVPMNSAQFEKQYKESSMFDKMMFERDEYEKEINKDKPSETFREAFKQAHNTPAVVSYYSQVKALTIRQFQLIWGDKGSLFVRYGDVFVKGLITASVFYMMPLDGSGAFARGGAYLFSLVFNAFIAQSELPAFMQGRRVLEKHKHFAMYRPSAFYLAQVIVDFPLAILQAILFQLCVYFLMGLALDAGKFFILFLNLIAITMCMNGFFRFLGAISPNFFIASQLSSSAFVALLIYSGYQIPYPDMHPWFMWVYWINPIAYSYKAMLSSELRGAEFSCDGTNGIPYGAGYTDQAYRSCTLPGAVPGASFVLGDEYLVQQYEYYTWQIWINIVAVILFFVLFTALTALSMEYIDLKQEGTITKVYKKGSAPQVESTENQLKQEKTRQEQELEAVTDGTTFSWHHIDYTVPVKGGTRQLLNNVAGIVKPGHLTALMGSSGAGKTTLLDVLSRRKTIGTIGGNIYMNGENLADDFERLTGYCEQMDVHNPNATIREALQFSAYLRQSADVSKEEKDAYVEQILDLLEMKKIGDALVGDLEEGTGISVEERKRLTIAIELVGKPKLLFLDEPTSGLDAQSSYNIIRFIRKLADAGWPVLCTIHQPSATLFEHFDHLLLLMRGGNTAYFGEIGKDSRTMIDYFESNGGPTCSPEANPAEYILECVGAGTAGKVTKDWAQVWSSSPEAAALEDELETIHSSVDHIVNRKVETYAQSFWLQLYLVYKRMNISWWRSPSYNIGRIFNVIFIGLFTGFTFWKLGSDSTDMQNRMLGLLTTMFMGNTMIILIQPRFMQERTWFRREYASKYYGWSPFALSCILVEIPYLVALSALFMFCYYWTTGLQNESDRIGYFFIQFVVYMFHSVTFGYAIAAFCESPTMSAILNPFFTTILYLFSGLFQTPKAMPRFWSSWMYWLDPYHYFIEGLVTNGLDGVPVVCSESSYIKIKVPPNQTCGDYMANFFADGGLGYVGNPNSTDYCDYCQYSVGNEYFETNIGWSFSNRWRNFGVLWVFTAFNVVLFVAFVYFFRKQKR

>MendPDR4

MDKSNSNWISTDYPNNINSDDHSSADEETDTVIDEGKNYYLDNLSRTQSGGAFGETNGNAVDIEDALEEYQSVRRELTRQSRKESGIISSDLEKGANVQEFDLTEYLSQQHSQLTAAGLKTKNMGVIWKNLKVEGLGADSRSITTNWSIISDLLQFWKWGKHKGTDFTILNDNNGFCKPGEMLLVLGRPGAGCSTLLRVLANMRAAYVNIEGDVSYGGIDAREFSRYFKGEVCYNEEEDLHYPTLTTLQTLRFALKNKTPSTRIPGESKDEFIDKLLYMLGNMLGLTKQMNTMVGNAFVRGLSGGERKRLSIAEQMTTHSSINCWDCATRGLDASSALDYVRSLRIMTDIMKKTTISTLYQASDSIFELFDKVMVLDEGHCIYFGPTFAAKKYFTDMGFYCSPRKSTPDFLTGICNMNEREVQPEFAGQVPVNSVQFEKLYKESTTYSEMLQERDAYEREISIDKPYETFKEAFKEANNTPFVLNYYEQIKALTVRQFQLIWGDKGSLITRYADIIVKGLICASIFYMMPRDGNGAFSRAGALVFFLIFNAFVAQSELPAFMEGRRVLEKHKHFAMYHPSAFYLAQVIVDFPLAIIQALVFELCVYFIMGLVLDAGKFFSFFAILVMVIMCMNSFFRFWGAISPNFYTASQISSIFFIAFIIYIGYQLPHADMPPWFVWLYWINPLAYSYKMLLSIEMSGMKFSCDGINAVPFGLQYSEQAYRSCILPGAAPGASFVLGDDYLAQYYNFYTRQQWINFMAVVLLFLLFTALTCLAMEYVNLKKEGTITKIYKKGHAPNAVFNKNTLEQQQTENSQQQELDSLNDGTTFSWHNVDYTVPVKEGTRQLLNNVAGIVKPGHLTALMGSSGAGKTTLLDVLARRKTIGTVSGNIYMNGENLADDFERLTGYCEQMDVHNPNATVREALQFSAYLRQPPDVPKKEKDEYVEQILELLEMGKIGDALIGDLEENAGISVEERKRLTIAIELVGKPKLLFLDEPTSGLDAQSSFNIIRFIRKLADAGWPVLCTIHQPSATLFEHFDHLLLLMRGGNTAYFGEIGKDSRTMIDYFESNGGPICSPDANPAEYILECVGAGTASKSNKDWSEVWLKSPEAAALERELETIHNTVDHTVKREVSMFALSFWQQLVLVYKRMNVSWWRTPSYNLGRVCNVCFIGLITGFSFWKMGNTPTDMQNRMFGLLTILFNGNTLIILIQPRFMQERTWFRREYASRYYGWSPFALSVVLVEIPYLITLAAIYMFFYYWSTGLQNESDRIGFFFIHLVVYFFNAVSMGLAVAAFCESPTMAAVINPFLITILLLFCGIFQPPMSMPAFWTSWMYYLDPYRYLVEGFITNALDGVNVICGKKDLIKIKTPPGQNCGEYMADFFSNGGLGYIESPNSTDYCNYCQYTKGNNYFEERIGWSFSNRWRDFGILWIYTIFNVAMFLLFAYLYRKQRR

>MlanPDR4

MDEQKPPINYPDSFYSNDNSKNIIHEEEGIPAYRTLSRGNFGENDGNAVCIDEALAEYDHVRRTLTEQSRNNSRIQSNNDLEKGQTTQDFDLTEYLSDQHTQITAAGLKPKNMGVIWKNLSVQGLGPDARSILTNWSVIQKTLQFWKWGKKNGTDVTILHDNDGFVKSGEMLLVLGRPGAGTSTLLRVLANMRASFTGVFGEVSYGGIDAKEFSKYFKGEVCYNEEEDLHYPTLTTEQTLRFSLKNKTPSTRLPGESKADFIESLLYMLGNMLGLTKQMNTMVGDAFIRGLSGGERKRLSIAEQMTTHSSINCWDCATRGLDASSALDYVRSLRIMTDIMKKTTISTLYQASDNIYSLYDKVMVLDESRCIYFGPASEAKQYFTDLGFYCPSRKSTPDFLTGLCNLNEREVRSDFQGTVPMNAVEFEKAYKESALYTRMLRERDVYEKQIVEDQPSMDFREAFKQANQKPFVINYFEQIKALTVRQYQLILGDKKSLYLRYSDVIIKGLITASIFYMMPLDGAGAFSRGGAFIFIVFFNSFVAQSELPAFMEGRRILEKHKHFALFHPSAFYIAQVIIDFPLSVLQAVLFQLCSYFMMGFALDAGKFFFLFINLIMITMCMNSFFRFWGSVSPNYYTASQISSIFFVAFIIYVGYQVPYPDMHPWLMWIYWINPLAYSNKAMMSSELAGAHFNCDGINAIPYGPGYTDQAYRSCVLPGAAPGASYVLGDDYLSQYYAYFTDQRWINFVAVVLLFVLFTALAALAMEYVDLKKGGTITKVYKKGRSPEVVSTQDKTQEKRAGQDQELETVTDGTVFSWQHIDYTVPVKGGSRQLLNNVGGIVKPGHLTALMGSSGAGKTTLLDVLARRKTIGKIDGHIYMNGEDLADDFERLTGYCEQMDIHNPNATVREALQFSAYLRQSADVSKKEKDDYVEQILELLEMTKIGDALVGDLEEGTGISVEERKRLTIGVELVGKPKLLFLDEPTSGLDAQSSFNIIRFIRKLADAGWPVLCTIHQPSATLFEHFDHLLLLVRGGKVAYFGEIGKDSRTMIDYFEANGGPICSPEANPAEYILECVGAGTSGKVTQDWGDVWTNSNEAQALEDELQTIHTTVDHKVTRKVHTYAQPFLQQLKHVLYRMNISWWRSPSYNLGRLFNVCFIGLITGFSFWKVGNTPTDLQNRMFALLTCLFMGNTMILLIQPRYMQERTWFRREYASKYYGWSPFALSCILVEIPYLIFVSAMFLVIYYWTTGLQNESERVIYFFLQFLIYMFHSVSFGYVLAAACENTTMAAVINPFFMTILLLFNGIFQPPSSMPNFWRVWMYPLDPYHYLVEGLIVNGLDGVKVVCGESDFIKIKAPLGETCGSYMADFFADGGLGYLGNPNSTDMCDYCQYSVGNDYYESRIGWDYSNRWRNFGILWIFTLFNVAVFLLCVYLFRKQRR

>MlusPDR5*

MDMPALQGMPQERHNRDEEYQNRNHYSDESTIEGVHGKEEISSDNETSTANYPNGGAFGEAEGNAVNIEEAMSNYEEIRRELTQQSRISRRKSMTPDQAEKGDVKDFDLTDFLREQTESSESQGFHPKHMGVVWKDLVVQGLGADAKTIPTNWTWIRDSVQFWKWGKHEGHDFTILKGNDGFCKDGEMLLVLGRPGAGCTSLLRVLANMRASYTKIEGSVHYGGIEAREFSKHFRGEVCYNEEEDLHYPTLTCKQTLRFALKNKTPGKRLDGESKGEFINKVLYMLGNMLGLTKQMNTMVGNAFVRGLSGGERKRLSIAEQMTTRSSINCWDCATRGLDASSALDYVRSLRIMTDILHKTTISTLYQASDSIFHLFDKVMVLDEGRCIYFGPTSTAKNYFEEMGFFCPSRKSTPDFLTGLCNMNEREYREGYKNQVPVNAVQFEKAYKESALYSQMMQERDEYEQKINQDRPDEKFRQAFVDAHQKHAPKHSPFVATYFNQVKSLTVRQFELILGDKGALVSRYGGVVVKGLIMASVFYMMPQDASGAFSRGGSFLFSLLFNALIAQAELSAFMQGRRVLEKHKHFALYHPSAFYIATVIADIPLALIQVIVFELCVYFMMGLVLDAGKFFTFFIILVVTNLCMNGFFRFWGAVSPNFFTASQLSSILLIACLVYCGYQIPYNQMHPWLMWIYWINPLAYGYKALISNELHGMHFTCEGPNSVPYGPSYTNTDYQTCNLPGAVPGQTYVLGDDYLHTAYGYETWQRWINFVAVLLFFFFFTILTALAMEYVDLQKEGSVTKVYKAGRAPKEIDESQALEQTVTENDEKMEAVDNGTVFSWHKMNYTVPVKGGKLKLLNDIGGIVKPGHLTALMGSSGAGKTTLLDVLAKRKTIGTIEGRIYLNGEPLGADFERTTGYCEQMDVHNPNATVREALKFSAYLRQPAEVPKEEKDAYVEQIIRLMEMEKIADALVGDLEAGIGISVEERKRLTIATELVGKPKLLFLDEPTSGLDAQSSFNIVRFIRKLADAGWPVLCTIHQPSATLFEHFDHLVLLVRGGKTAYCGEIGPNSRTMIEYFESNGGPKCSPQANPAEYILECVGAGTAGKATKDWSEVWSGSPQAKALDEELEAIHQGITPGLKNHSTPYSLTFAQQFWLVYKRMNVSWWRCPTYNMGRLFNVCFIGLISGFSFWKLGATPSDLQNRMFSVFTTLLMSNALIILAQPRFMQERMWFRREYASKYYGWAPFALSCILVEIPYLIVFSAIFLFCFYWTAGLQNVSDRVGFFYIHFTVFLFYSVSLGFMIAAFSATPPMAAVINPFFTSILILFAGIMQPPASMPYFWRAWMYWLDPYHYVIEGLVVNVMDSVEVVCGDNDWTPINSPPGMNCGDYMADFFNAGGNGYLQSNSSTGVCNYCPYTKGNQFYEERIGWHFDNRWRDFGILCAYCVFNIFAFMFFVFLFRKAKR

>MambPDR5

MDMPALQGMPQERHNRDEEYQNRNHYSDESTIEGVHGKEEVNSDNETSTAHYPNGGAFGEAEGNAVNIEEAMSNYEEIRRELTQQSRISRRKSMTPDQAEKGDVKDFDLTDFLREQTESSESQGFHPKNMGVVWKDLVVQGLGADAKTIPTNWTWIRDSLRFWKWGKHEGHDFTILKGNDGFCKDGEMLLVLGRPGAGCTSLLRVLANMRASYTNIEGTVHYGGIEAQEFSKHFRGEVCYNEEEDLHYPTLTCKQTLKFALKNKTPGKRLDGESKGEFINKVLYMLGNMLGLTKQMNTMVGNAFVRGLSGGERKRLSIAEQMTTRSSINCWDCATRGLDASSALDYVRSLRIMTDILHKTTISTLYQASDSIFHLFDKVMVLDEGRCIYFGPTSTAKNYFEEMGFFCPNRKSTPDFLTGLCNMNEREYREGYKNQVPVNAVQFEKAYKESTLYSQMMQERDEYEQKINQDRPDEKFRQAFLDAHQKHAPKHSPFVATYFNQVKSLTVRQFELILGDKGALVSRYGGVVVKGLIMASVFYMMPQDASGAFSRGGSFLFSLLFNALIAQAELSAFMQGRRVLEKHKHFALYHPSAFYIATVIADIPLALIQVIVFELCVYFMMGLVLDAGKFFTFFIILVVTNLCMNGFFRFWGAVSPNFFTASQLSSILLIACLVYCGYQIPYNQMHPWLMWIYWINPLAYGYKALISNELHGMHFTCEGYNSVPYGPSYTNTDYQTCNLPGAVPGQTYVLGDDYLHTAYGYETWQRWINFVAVLLFFFFFTILTALAMEYVDLQKEGSITKVYKAGCAPKEVDESQALEQTVTENDEKMEAVTDGTTFSWHHIDYTVPIKGGKLKLLNDIGGYVKPGHLCALMGSSGAGKTTLLDVLAKRKTIGTIEGRIYLNGEPLGPDFERTTGYCEQMDVHNPNATVREALKFSAYLRQPAEVPKEEKDAYVEQIIRLMEMEKIADALVGDLEAGVGISVEERKRLTIATELVGKPKLLFLDEPTSGLDAQSSFNIVRFIRKLADAGWPVLCTIHQPSATLFEHFDHLVLLVRGGKTAYFGEIGPNSRTMIEYFESNGGPKCSPQANPAEYILECVGAGTAGKATKDWSEVWSASNEAKAVEAELEEIHQGITPDHKNTKSAYALSFAQQFWLVYKRMNVSWWRCPTYNMGRLFNVCFIGLISGFSFWKLGATPSDLQNRMFSVFTTLLMSNALIILAQPRFMQERMWFRREYASKYYGWAPFALSCILVEIPYLIVFSAIFLFCFYWTAGLQNVSDRVGFFYIHFTVFLFYSVSLGFMIAAFSATPPMAAVINPFFTSILILFAGIMQPPASMPYFWRAWMYWLDPYHYVIEGLVVNVMDSVEVVCGDNDWTRINSPPGMNCGDYMADFFNAGGNGYLQSNTSTGVCNYCPYTKGNQFYEERIGWHFDNRWRDFGILCAYCVFNIFAFMFFVFLLRKAKR

>McicPDR5

MDMPALQGMPQERQNRDEEYQNRAHYSDESTIEGVHGKEEINSDNETTTHYPNGGAFGESEGNAVNIEEAMSNYEEIRRELTQQSRISRRKSMTPDQAEKGDVKDFDLTDFLREQTESSESQGFHPKHMGVVWKNLVVQGLGADAKTIPTNYTWIRDSLQFWKWGKHEGHDFTILKGNDGFCKDGEMLLVLGRPGAGCTSLLRVLANMRASYTNIEGSVHYGGIEAQEFSKYFRGEVCYNEEEDLHYPTLTCKQTLKFALKNKTPGKRLDGESKGEFINKVLYMLGNMLGLTKQMNTMVGNAFVRGLSGGERKRLSIAEQMTTRSSINCWDCATRGLDASSALDYVRSLRIMTDILHKTTISTLYQASDSIFHLFDKVMVLDEGRCIYFGPTSTAKNYFEEMGFFCPSRKSTPDFLTGLCNMNEREYREGYKNQVPVNAVQFEKAYKESALYSQMMQERDEYEQKINQDRPDEKFRQAFVDAHQKHAPKHSPFVATYFNQVKSLTVRQFELILGDKGALVSRYGGVVVKGLIMASVFYMMPQDASGAFSRGGSFLFSLLFNALIAQAELSAFMQGRRVLEKHKHFALYHPSAFYIATVIADIPLALIQVIVFEICVYFMMGLVLDAGKFFTFFIILVVTNLCMNGFFRFWGAVSPNFFTASQLSSILLIACLVYCGYQIPYNQMHPWLMWIYWINPLAYGYKALISNELHGMHFTCEGYNSVPYGPSYTNTDYQTCNLPGAVPGQTYVLGDDYLHTAYGYETWQRWINFVAVLLFFFFFTILTALAMEYVDLQKEGSITKVYKAGRAPKEIDESQALEQTVTENDEKMEAVNDGTTFSWHHMNYTVNIKGGKLKLLNDIGGIVRPGHLTALMGSSGAGKTTLLDVLAKRKTIGKVEGRIYLNGEPLGSDFERTTGYCEQMDVHNPNATVREALKFSAYLRQPADVPKEEKDAYVEQIIRLMEMERIADALVGDLEAGIGISVEERKRLTIATELVGKPKLLFLDEPTSGLDAQSSFNIVRFIRKLADAGWPVLCTIHQPSATLFEHFDHLVLLVRGGKTAYCGEIGPNSRTMIEYFESNGGPKCSPQANPAEYILECVGAGTAGKATRDWSEVWAASDEAKALDAELEEIHQSIDPNKKNHSTPYSLSFAQQFWLVYKRMNVSWWRCPTYNMGRLFNVCFIGLISGFSFWKLGATPSDLQNRMFSVFTTLLMSNALIILAQPRFMQERMWFRREYASKYYGWAPFALSCILVEIPYLIVFSAIFLFCFYWTAGLQNISDRVGFFYIHFTVFLFYSVSLGFMIAAFSATPPMAAVINPFFTSILILFAGIMQPPASMPYFWRAWMYWLDPYHYVIEGLVVNVMDGVEVVCGDNDWTAINSPPGMNCGEYMADFFNAGGNGYLQSNSSTGVCNYCPYTKGNQFYEERIGWHFDNRWRDFGILCAYSVFNIFAFMFFVFLLRKAKR

>MracPDR5

MDMPALQGMPQERQNRDEEYQNRNHSSDESTIEGVHGKEEVISDNETTTHYPNGGAFGESEGNAVNIEEAMSNYEEIRRELTQQSRISRRKSMTPDQAEKGDVKDFDLTDFLREQNESSESQGFHPKHMGVVWKDLVVQGLGADAKTIPTNYTWFRDSLQFWKWGKHEGHDFTILKGNDGFCKDGEMLLVLGRPGAGCTSLLRVLANMRASYTNIEGSVHYGGIEAREFSKHFRGEVCYNEEEDLHYPTLTCKQTLSFALKNKTPGKRLDGETKGEFINKVLYMLGNMLGLTKQMNTMVGNAFVRGLSGGERKRLSIAEQMTTRSSINCWDCATRGLDASSALDYVRSLRIMTDILHKTTISTLYQASDSIFHLFDKVMVLDEGRCIYFGPTNTAKNYFEDMGFHCPSRKSIPDFLTGLCNMNEREYREGYKTQVPVNAVQFEKAYKESALHAQMMKERDEYEQKINQDRPDEKFRQAFLDAHQKHASKRSPFVATYFNQVKSLTVRQFQLILGDKGALISRYGGVIVKGLIMASVFFMMPTDASGAFSRGGAFLFSLLFNALIAQAELSAFMQGRRVLEKHKHFALYHPSAFYIATVIADVPLAIVQVLVFEICVYFMMGLVLEAGKFFTFFIILVVTNLCMNGFFRFWGAVSPNFFTASQLSSILLIAALVYCGYQIPYTQMHPWLMWIYWINPLAYGYKALISNELHGMHFSCEGANSVPYGPTYTNTDYQTCNLAGAQPGQTYVLGDDYLHTAYGYETWQRWINFVAVLLFFFFFTILTALAMEYIDLQKEGSITKVYKAGRAPKEIDESQALEQTVEDNDEKMDAVDDGTTFSWHHMDYTVPVKGGKLKLLNDIGGIVRPGHLTALMGSSGAGKTTLLDVLAKRKTIGKIEGRIYLNGEPLGPDFERSTGYCEQMDVHNPNATVREALKFSAYLRQPASVPKEEKDAYVEQIIRLMEMERIADALVGDLEAGIGISVEERKRLTIATELVGKPKLLFLDEPTSGLDAQSSFNIVRFIRKLADAGWPVLCTIHQPSATLFEHFDHLVLLVRGGKTAYCGEIGPNSRTMIEYFESNGGPKCSPQANPAEYILECVGAGTAGKATKDWSEVWTGSNEAKALDAELEEIHQSIDLNKKNHSSAYSLTFAQQFWLVYKRMNVSWWRCPTYNMGRLFNVCFIGLISGFSFWKLGSTPADMQNRMFSVFTTLLMSNALIILAQPRFMQERSWFRREYASKYYGWAPFALSCVLVEIPYLIVFSAIFLFCFYWTAGLQNASDRVGFMYIHFTVFLFYSVSLGFMIAAFSATPPMAAVINPFFTSILILFAGIMQPPSSMPYFWRAWMYWLDPYHYVIEGLVVNVMDSVEVVCGDSDFTKITPPPGTNCGDYMADFFASGGNGYIANNASTDICNYCPYTKGNQFYEQRIGWHFDNRWRDFGILCAYSVFNIFAFMFFVFLLRKAKR

>MendPDR5

MDMQALPNMQQNRHNRDEEYQNRNFHSDESTIEGVHDKEEVNRDSSEEYNNNTRNNGGAFGETEGNAVNIEDAMSNYEEIRRELTQQSRISRRKSLSPGEAEKGDVKDFDLTEFLRDQSDQGEAAGFHPKHMGVIWKNLVVQGLGADAKTISTNWTWIRDTVKFWKWGKHEGTDFTILKGNDGFCKDGEMLLVLGRPGAGCTTLLRVLANMRASYTNIDGEVSYGGIEAQEFSKHFRGEVCYNEEEDLHYPTLTTKQTLSFALKNKTPGKRLDNESKGEFINKILYMLGNMLGLTKQMNTMVGNAFVRGLSGGERKRLSIAEQMTTRSSINCWDCSTRGLDASSALDYVRSLRIMTDIMHKTTIATLYQASDSIFHLFDKVMVLDEGRTIYFGPTSSAKSYFEEMGFVCPDRKSTPDFLTGLCNANERQYREGFKDKVPVNSVQFEKAYLDSSISAAMIEERNQYERKIQSDRPDEKFRQAFADAHQKHAPKRSPFVATYYQQVKSLTVRQFQLIIGDKGALVSRYGGVVVKGLIMASVFYMMPVDATGAFSRGGAFLFSLLFNALIAQAELSAFMQGRRVLEKHKHFALYHPSAFYIATVIADIPLAIVQVLIFEICVYFMMGLVLDAGKFFTFFIILVVTNLCMNGFFRFWGAISPNFFTASQLSSIFLIACLVYCGYQIPYNQMHPWLMWIYWINPLAYGYKALISNELTGMQFSCEGYNSVPYGPSYTDDAYTTCNLPGAVPGANTVSGDAYLHTAYGYETWQRWINFVAVILFFFFFTILTALAMEYVDLQKEGSVTKVYKAGKAPKELDESEALEQTVTEQDEKMEAINEGTTFSWHHMDYTVPVKGGSLKLLNDIGGIVRPGHLTALMGSSGAGKTTLLDVLAQRKTIGKIEGRIYMNGEPLGPDFERTTGYCEQMDVHNPNATVREALKFSAYLRQNADVPKEEKDAYVEQIIRLMEMERIADALVGDLEAGVGISVEERKRLTIATELVGKPKLLFLDEPTSGLDAQSSYNIVRFIRKLADAGWPVLCTIHQPSATLFEHFDHLVLLVRGGKTAYCGEIGDNASTMIEYFETNGGPKCSPNANPAEYILECVGAGTAGKASQDWSEVWSKSPQAKALDEELEAIHSNIDTNHKNNKSAYSLTFWQQFWLVYKRMNVSWWRCPTYNMGRLFNVCFIGLISGFSFWKLGNSPADMQNRMFSVFTTLLMSNALIILAQPRFMQERSWFRREYASRYYGWAPFALSCILVEIPYLIFFSAIFLFCFYWTAGLQNESDRVGFFFIHFAVFLLYSVSLGFMIAAFSATPPMAAVINPFFTSILILFAGIMQPPASMPKFWSAWMYWLDPYHYVIEGLVVNVMDSVEVVCGEGQFTPIPIPAGSTCGSYMSDFFKSGGAGYIENENASDICNYCPYKVGNQFYEERIGWSFDNRWRDFGILCAYYVFNIFAFMFFVFLFRKAKR

>MlanPDR5

MDMPALQGMQQDRHNRDEEYQTRHEYSDESTIEGVHGKEEINSDNDESAVYPNELPNGGAFGEREGNAVNIEDAMSNYEEIRRELTQQSRMTRKSMTADQAEKGAVKDFDLTEFLREQHDQGETAGFHPKHMGVVWKNLVVQGLGADAKTIPTNYTWLRDFAQFWKWGRHTGTDFTILKGNDGFCKDGEMLLVLGRPGAGCSSLLRVIANMRASYTNIEGSVHYGGIEAQEFSKYYRGEVCYNEEEDLHYPTLTTKQTLSFALKNKTPGKRFEGESKGEFINKVLYMLGNMLGLTRQMNTMVGNAFVRGLSGGERKRLSIAEQMTTRSSINCWDCATRGLDASSALDYVRSLRIMTDILHKTTISTLYQASDSIFDLFDKTMVLDEGRCIYFGPTATAKSYFEDMGFYCPDRKSTPDFLTGLCNMNEREYRDGMKNKVPINSVQFEKAYKESAMYAAMMKERDEYEQKINQDRPDEKFRQAFVDAHQKYAPVRSPFVATYYQQVKSLTIRQFQLIWGDKGALVSRYGGVVVKGLIMASVFFMMPQDASGAFSRGGSFLFSLLFNALIAQAELSSFMQGRRVLEKHKHFALYHPSAYYIAQVIADVPLALVQVIVFEICVYFMMGLVLEAGKFFTFFVILVVTNLCMNGFFRFFGAVSPNFFTASQLSSILLIACLVYCGYQIPYNQMHPWLMWIYWINPLAYGYKALISNELTGMHFTCEGANSVPYGPTYTNTDYQTCNLAGAQPGANYVLGDDYLHYAYGYETWQRWIDFVAVLLFFFLFTILTALGMEYIELQKEGSVTKVYKAGRAPKEIDESQALEQTVTEQDEKMEAVSEGTTFSWHHMDYTVPIKGGKLKLLNDIGGIVRPGHLTALMGSSGAGKTTLLDVLAKRKTIGKIEGRIYMNGEPLGPDFERSTGYCEQMDVHNPNATVREALKFSAYLRQAADVPKEEKDNYVEQIIRLMEMERIADALVGDLEAGVGISVEERKRLTIATELVGKPKLLFLDEPTSGLDAQSSYNIVRFIRKLADAGWPVLCTIHQPSSTLFEQFDHLVLLVRGGKTAYCGEIGKGGATMIDYFETNGGPKCSPSANPAEYILECVGAGTAGKATKDWSEVWTASPQAKALDAELEEIHQSINKDHKNRRSPYSLSFFQQFWIVYKRMNVSWWRCPTYNMGRLFNVCFIGLLSGFSFWKLGNSPTDMQNRMFSVFTTLLMANALIILAQPRFMQERSWFRREYASRYYGWAPFALSCILVEIPYLIFFAAIFLFCFYWTAGLQTDSDRVGFFFVHFMMFLFYSVSLGFMIAAFSATPPMAAVINPFFTSILILFAGIMQPPNDMPYFWSAWMYWLDPYHYVIEGLVVNVMDSVEVHCSEGQFTKIPVPPGQTCGSYMNDFFTNGGYGYVANNASTELCDYCPYTIGNQFYEGRIGWHFDNRWRDFGILCAYNVFNVFAFMFFVFLLRKAKR

>MlusPDR6

MEEKPTEHIEVPHHHPNYNYASSTEDTIAHDDNFGRNGTYGETDVNQVNIQGAKNEYQTLKRELSHMSRKSTPSKVEEGQADSDDFNLDEFLHGIHREQEENGQKRKHLGVSWKNLHVEGLGADAYTIPTVLSNIMAVLKFWKLFKKKDASTKVIIDDLTGCCRDGEMLLVLGRPGAGCTSFLKVIANMRGAYTYVGGEVNYGGINPKEFASKYRGQVCYNEEEDQHYPTLTTKQTLQFALRTKTPGKRLNEESKKDFVDRVIYLLGNMLGLTKQMNTMVGNAFVRGLSGGERKRLSIAEQMTTRSTINCWDCSTRGLDAASALDYVRSLRIMTDVFNITTIATLYQASNSIFNLFDKVLLLDEGYCIYYGPTSGARDYFDTLGFHRPSRKSMPDFLTGLCNPVEREFKPGFEDSAPKHASEFQAAYLQSEVYQTMLRDFESYNEAVEKENKAANFADAIQEEHQKRAKKSRPYIASFYQQVKALTIRQHHLLIKDREALISRYGTILIQSLITASCFFQLPLTGTGAFSRGGALFFSVLFNSFISQSELVRFLTGRPILEKHKQYALYRPSAFYIAQVIMDIPYALVQVLLFEICAYFMMGLNLTAGRFFTFFVVLFFLNMCMNGFFRFFGAITSSFFLATQITGVLLIAITSYTGYTIPYKKMHPWLFWIYYINPITYAYKALLSNEMSGQVYSCDGIGNAVPYGPGYDDWNYMVCTMQGGVAGENFVRGDSYLLEALSYKPWQLWAPDFVVVVAFFLFFTFITALAMELVGMSKSSTLTKLYLPGKAPKPRTIEEEDQRRLDQQKVTDNMDKMSTGTTFSWQHVNYWVPFSGGPLHLLNDISGIVKPGHLCALMGSSGAGKTTLLDVLARRKTIGKVEGNVFLNGEALMNDFERITGYCEQMDIHQPAVTVREALRFSAYLRQPADVPKEEKDAYVEQIILLLEMDDIGDAQIGDVGSGFGISVEERKRLTIGMELVGKPKLLFLDEPTSGLDAQSSFNIIRFIRKLADAGWPVLCTIHQPSAILFEYFDHLLLLVRGGRTAYYGEIGKDSRTMIGYFESNGGPKCSPEANPAEYILEVVGAGTAGKATKDWAEVWQSSEEAKALTAELDEIERTADKNPTREAKMYATPLSTQFRLVMGRMTLAYWRAPDYNIGRFLNIMFTSLVTGFTFWKLGSSSSDMMNKVFALFSTFIMAMTMIILAQPKFMTERLYFRREYASRYYSWLAFGISAVLVEIPYIIFFAAAFMFGFYWTAGMNNTPESCGYFYITFVVLVCWAVTLGFCIAAVAELPTMAAVINPLFISILILFCGLMQAPSAMPRFWSSWMYWLDPFHYYIEGLAVNELADLQVECSDEDLLKFPPPPGQTCGQYMANFFSNGATGYVANPDAVQPEQCGYCTYKSGPEFYETGMQWSASHKWRNFGILIAFFIFNVFVFILVVYLRRKGRR

>MambPDR6

MEEAPKDDIEVSQHHPNYNYESSTGDTIAHDDSFGRNGAYGETDVNQVNVQGAKNEYQTLKRELSQMSRKSTPSKAEEGQAISEDFNLDEFLHGIHREQEENGQKRKHLGVSWKNLHVEGLGADAYTIPTVFSNIMTVLKFWKLFKKKNASTKVIIDDLTGCCRDGEMLLVLGRPGAGCTSFLKVIANMRGAYTHVGGEVNYGGIDPKEFASKYRGQVCYNEEEDQHYPTLTTKQTLQFALRTKTPGKRLNEESKKDFVDRVIYLLGNMLGLTKQMNTMVGNAFVRGLSGGERKRLSIAEQMTTRSTINCWDCSTRGLDAASALDYVRSLRIMTDVFNITTIATLYQASNSIFNLFDKVLLLDEGYCIYYGPISGAKDYFDALGFHRPSRKSLPDFLTGLCNPVEREFKPGFEDSAPKHASEFQTAYLQSEIYQTMLKDFEAYSEAVEKENKAANFADAIHEEHQKRAKKTTPYIASFYQQVKALTIRQHHLLIKDREALISRYGTILIQSLITASCFFQLPLTGTGAFSRGGALFFCVLFNSFISQSELVRFLTGRPILEKHKQYALYRPSAFYIAQVIMDIPYALVQVLLFQICAYFMMGLNLTAGRFFTFFVVLFFLNMCMNGFFRFFGAITSSFFLATQITGVLLIAITSYTGYTIPYKKMHPWLFWIYYINPITYAYKALISNEMSGQVYSCNGVGNAVPYGPGYNDWNYMVCTMQGGVAGEDFVRGDNYLLEALSYKPWQLWAPDFVVVVAFFLFFTFITAMAMELVGMSKSSTLTKLYLPGKAPKPRTIEEEDERRLAQQKVTDSMEKMSSGTTFSWQHVHYHVPFKGGPLHLLNDISGIVKPGHLCALMGSSGAGKTTLLDVLARRKTIGKVEGNVLLNGEALMTDFERITGYCEQMDIHQPAVTVREALRFSAYLRQPFEVPKEEKDAYVEQIILLLEMDDIGDAQIGDVGSGFGISVEERKRLTIGMELVGKPQLLFLDEPTSGLDAQSSFNIIRFIRKLADAGWPVLCTIHQPSAILFEYFDHLLLLVRGGRTAYFGEIGKDSHTMIDYFESNGGPKCSPEANPAEYILEVVGAGTAGKATKDWADVWQNSDEAKSLAAELDEIDRTADKNPTRKANTYATPLSTQFRLVMGRMTLAYWRAPDYNIGRFLNIMFTSLVTGFTFWKLGSSSSDMMNKVFALFSTFIMAMTMIILAQPKFMTERLYFRREYASRYYSWLPFGISAVLVEIPYIIFFAAAFMFGFYWTAGMNNTPESCGYFYITFVVLVCWAVTLGFCIAAVAELPTMAAVINPLFISILILFCGLMQAPSAMPKFWSSWMYWLDPFHYYIEGLAVNELADLLVECSDEDLLKFPPPPGQTCGQYMANFFSNGATGYVANPDAVQPEQCGYCTYKSGPQFYETGMQWSASHKWRNFGILIAFFIFNVFVFIAVVYWRRKGRR

>McicPDR6

MEEKPTDHIEVSHHHPNYNYASSTEDTIAHEDNFGRNGTYGEADVNQVNVQGAKNEYQTLKRELSQMSRKSTPSKAEEGQADVDDFNLDEFLHGIHREQEENGQKRKHLGVSWKNLHIEGLGADAFTIPTVFSNIMTVLQFWKLFKKKNASTKVIIDDLTGCCRDGEMLLVLGRPGAGCTSFLKVIANMRGAYTYVGGDVNYGGIDPKEFASKYRGQVCYNEEEDQHYPTLTTKQTLQFALRTKTPGKRLNEESKKDFVDRVIYLLGNMLGLTKQMNTMVGNAFVRGLSGGERKRLSIAEQMTTRSTINCWDCSTRGLDAASALDYVRSLRIMTDVFNITTIATLYQASNSIFSLFDKVLLLDEGYCIYYGPTSGAKDYFDALGFHRPSRKSLPDFLTGLCNPVEREFKPGFEDSAPKHASEFQAAYLKSEVYQTMMSDFESYNEAVEKENKAANFADAVNEEHQKRAKKSRPYIASFYQQVKALTIRQHHLLIKDREALISRYGTILIQSLITASCFFQLPLTGTGAFSRGGALFFSVLFNSFISQSELVRFLTGRPILEKHKQYALYRPSAFYVAQVIMDIPYALVQVLLFEICAYFMMGLNLTAGRFFTFFVVLFFLNLCMNGFFRFFGAITSSFFLATQITGVLLIAITSYTGYTIPYKKMHPWLFWIYYINPITYAYKALISNEMSGQVYSCDGIGNAVPYGPGYDDWNYMVCTMQGGVAGENFVRGDNYLLEALSYKPWQLWAPDFVVVVAFFLFFTFITALAMELVGMSKSSTLTKLYLPGKAPKPRTIEEEDARRVAQQKVTDSMDKMSTGTTFSWQHVHYHVPFKGGPLHLLNDISGIVKPGHLTALMGSSGAGKTTLLDVLARRKTIGKVEGNVFLNGEALMTDFERITGYCEQMDIHQPAVTVREALRFSAYLRQPSDVPREEKDAYVEQIILLLEMDDIGDAQIGDVGSGYGISVEERKRLTIGMELVGKPQLLFLDEPTSGLDAQSSFNIIRFIRKLADAGWPVLCTIHQPSAILFEYFDHLLLLVRGGRTAYYGEIGKDSRTMIDYFESNGGPKCSPEANPAEYILEVVGAGTAGKATKDWADVWENSEEAKVLAAELDEIEKTADKNPTRKADTYATSLSTQFRLVMGRMTLAYWRAPDYNIGRFLNIMFTSLVTGFTFWKLGSSSSDMMNKVFALFSTFIMAMTMIILAQPKFMTERLYFRREYASRYYSWLPFAISAVLVEIPYIIFFAAAFMFGFYWTAGMNNTPESCGYFYITFVVLVCWAVTLGFCIAAVAELPTMAAVINPLFISILILFCGLMQAPSAMPRFWSSWMYWLDPFHYYIEGLAVNELADLQVECSDEDLLKFPPPPGQTCGQYMANFFSYGATGYVANPDAVQPEQCGYCTYKSGAEFYETGMQWSASHKWRNFGILIAFFIFNVFVFILVVFWRRKGRR

>MracPDR6

MEENPTDRIEVSNHPNYNYASSTEDTIAHDYSCGRNGTYGEPDVNQVNIQGAKDEYQSLKRELSQMSRHSDPSKAEEGQVETDDFNLDDFLHGIHKEQEENGHKRKHLGVSWKNLHIEGLGADAFTIPTVFSNVMKVLKFWKLFKKSNASTKVIVDDLTGCCKDGEMLLVLGRPGAGCTSFLKVIANIRGAYTHIGGDVNYGGIDPKEFAKKYRGQVCYNEEEDQHYPTLTTKQTLQFALRTKTPGKRLNEESKKEFVDRVIYVLGNMLGLTKQMDTMVGNAFVRGLSGGERKRLSIAEQMTTRSTINCWDCSTRGLDAASALDYVRSLRIMTDVFNITTIATLYQASNSIFNLFDKVLLLDEGYCVYYGPTSGAREYFDALGFHRPSRKSLPDFLTGLCNPVEREFKPGFENTAPKHASEFQEAYLKSEIYQTMMQDFESYNETVEKESKARNFADAIHEEHQKRAQKNTPYIASFYQQVKALTIRQHHLLIKDQQALISRYGTILIQSLITASCFFQLPLTGTGAFSRGGALFFCVLFNSFISQSELVRFLTGRPILEKHKQYALYRPSAFYIAQVIMDIPYALVQVLLFEICAYFMMGLNLTAGRFFTFFIVLFFLNMCMNGFFRFFGAITSSFFLATQITGVLLIAITSYTGYTIPYKKMHPWLFWIYYINPITYAYKALISNEMSGQVYSCEGIGNAVPYGPGYDDWNYMVCTMAGGVAGENFVRGDAYLLEALSYKPWQLWAPDFVVVVAFFIFFTFLTAMAMEFIGMSKSSTLTKLYLPGKSPKPRTIEEEDERRLAQQKVTDSMDKMSTGTTFSWQHVHYHVPFKGGPLHLLNDISGIVKPGHLTALMGSSGAGKTTLLDVLARRKTIGKVEGNVFLNGESLMTDFERITGYCEQMDIHQPAVTVREALRFSAYLRQPSDVPKEEKDAYVEQIILLLEMDDIGDAQIGDVGSGFGISVEERKRLTIGMELVGKPQLLFLDEPTSGLDAQSSFNIIRFIRKLADAGWPVLCTIHQPSAILFEYFDHLLLLVRGGRTAYYGEIGQDSRTMIDYFESNGGPKCSPEANPAEYILEVVGAGTAGKATKDWADVWENSNEAKTLAAELDEIEKTADKNPTRKADTYATPLSTQFRLVMTRMTIAYWRAPDYNIGRFLNIMFTSLVTGFTFWKLGNSSSDMMNKVFALFSTFIMAMTMIILAQPKFMTERLYFRREYASRYYSWLPFGVSAVLVEIPYIVFFAASFMVGFYWTAGMNNTPESCGYFYIIFVVLVCWAVTLGFVIAAVAELPTMAAVINPLFISILILFCGLMQSPEAMPHFWSSWMYWIDPFHYYIEGLAVNELASLIVVCSDEDLLKFPPPPGQSCGQYMANFFSNGATGYVANPDAVQPERCGYCTYSSGPEFYETGMQWSASHKWRNFGILIAFFIFNVCVFILLVFLRRKGRR

>MlanPDR6*

MKGEASNLNHASSAGTNNDQCGHCDSYGEPDANQVHIRGAEDEYQSLKRELTQRSRHADLAKAEEGEADQEEFNLDEFLHGISRQQEENGSKRKHLGVSWKNVHVEGLGADAFTIPTVFSYIMTVLKFWKLFKPKKASTKIILSDISGCCRDGEMLLVLGRPGAGCTSFLKVIANIRSGFTKVDGDVNYGGIDPKTFAKHYRGQTCYNEEEDQHYPTLTTKQTLQCALRVKTPGKRLNNETRKDFVDRVLYVLGNMLGLTKQMDTMVGNAVVRGLSGGERKRLSIAEQMCTRSTINCWDCSTRGLDAASALDYVRSLRIITDVFNITTIATLYQASNSIFNLFDKVLLLDEGYCIYYGPTSGAKQYFEDLGFYCPPRKSTPDFLTGLCNPIEREFLPGFEDSAPKYATDFQERYRDSQICKSMNDDIKDYEQVVLNEGHANKFAEAIREEHQKGASKKTPYIASFYQQVKALTIRQHHLLIKDRQALISRYGTILIQALITASCFFQIPLTGTGAFSRGGALFFSVLFNSFISQSELVRFLTGRPILEKHKQYALYRPSAFYMAQVIMDIPYAVIQVLLFQICAYFMMGLNLTAGRFFTFFVVLFFLNICMTGFFRFFGAITSSFFLATQITGVLLIAMNSYTGYTIPYKKMHPWLFWIYYIDPITYAYKALLSNEMSGQIYSCGGIGNAVPYGPGYDNFNYQVCTMQGGEPGEPFVRGDSYLLAALDYKPWQLWAPDFVVVVAFFIFFTFLIGLTMEYGSMNKSSTLTKLYKPGKAPKSRTKKEEKERLQAQNKITENIDTISTGTTFSWQHVNYTVPIKGGQLQLLNDIGGIVKPGHLTALMGSSGAGKTTLLDVLARRKTIGKVEGDVFLNGEVLMNDFERITGYCEQMDIHQPAVTVREALRFSAFLRQPAEVPKEEKEAYVEEIILLLEMGDIGDAQIGNVGSGYGISVEERKRLTIGMELVGKPQLLFLDEPTSGLDAQSSFNIIRFIRKLSNAGWPVLCTIHQPSSILFGYFDHLLLLVRGGRTAYYGEIGQDSRTMINYFESNGGPKCSPDANPAEYILEVVGAGTVGKAAKDWAKVWENSNEAQALANELDEIEKTAERTPKRQAKTYSTPLSTQFRLVYGRMRLAYWRAPEYNIGRFLTIMFTSLITGFTFWKLGNSSSDLLYKVFALFSTFIMAMTIIILSQPRLITERMYFRREYSSRYYSWIPFGLSAVLVEIPYVLVFAFCFMFGFYWTAGMKNTAEACGYFYITFVALICWAFTLGFVIAAFAEQPTMAAMINPLFISTLILFCGLMQSPLAMPRFWSSWMYWLDPFHYYIEGLAVNELGDLVVECTDEDLLRFSPPPGQTCGQYMANFFTRGGAGYIANPDATQPEQCGYCTYTSGAQFYELGMEWSAAHKWRNFGILVGFFIFNVFIFNLLVYWKRKGRR

>MlusPDR7*

MEEKKNALYTEQVEDARLRDQPNYAHSNNSTIDADVSAHNKENCGQNDTFGESEANQVNIEDAKNEYQELRRELSTKSQRLSLSKAEEGASNSEDFDLDQFLHGISKAQDENGHKRKHLGVAWKNLHVEGLGADAFTIPTVFSNILSVVQFWKLFKKNKASTKVIIDDLSGCCKDGEMLLVLGRPGAGCTSFLKVIANMRDSYTYIGGDVSYGGIDPKTFAERFRGQVCYNEEEDQHYPTLTTKQTLQFALRTKTPGKRMPGETKADFVDRVLYMLGNMLGLTKQMNTMVGNAFVRGLSGGERKRLSIAEQMTTSSTINCWDCSTRGLDAASALDYVRSLRIMTDVFDITTIATLYQASNSIFNLFDKVLLLDEGRCIYFGPTSGAKQYFDGLGFHCPPRKSLPDFLTGLCNPLEREFKPGFEDSAPKHAVEFQEKYYQSEVHKTMMRDLEDYEKTIAAENKANAFEDAVHEEHQKRAPKKTPYIASFFQQVKALTIREHHLLIKDREALISRYGTMLIQGLITASCFFMLPFTGSGAFSRCGALFFSAMFNVFISQSELVRFLMGRPILEKHKQYALYRPSAYYIAQVTMDIPYSLAQVLLYNICSYFMMGLNLTAGRFFTSFLILFFLTMCMTGFFRFFGSITSSFFLATQITGVLLIATTTYTGYTIPYDKMHPWLFWIYYINPITYAYKALVSNEMEGQLYSCEGAGNAVPFGPTYQDWNYKVCTMAGGNPGENFVRGDDYLRTALSYNPEDLWAPDFVVVVAFFLFFTVLTALAMEYVKLNKAGSLTKLYLPGKAPKPRTTEEEDERRRQQDKITDNMDKMSTGTTFSWQHVNYRVPIKGGSLQLLNDINGIVKPGHLCALMGSSGAGKTTLLDVLARRKTIGTVEGDIFLNNEALMNDFERITGYCEQMDVHQPAVTVREALRFSAYLRQPAEVSKEEKDEYVEQIIQLLEMTDIGDAQIGQVETGFGISVEERKRLTIGMELVGKPQLLFLDEPTSGLDAQSSFNIIRFIRKLADAGWPVLCTIHQPSAILFEHFDHLLLLVRGGRTAYYGEIGQDSRTMIDYFESNGGPKCSPDANPAEYILEVVGAGTAGKASKDWAEVWQASEECKALGAELDEIMATANKNPTREAHTYATSLTTQFRLVYGRMALAYWRSSDYNIGRFLNLMFTSLITGFTFWKLGESSSDMMYKIFALFGTFIMCFTMIILAQPKFMTERMYFRREYSSKYYSWLPWGVSAILVEIPYVFFFSATFMFGFYWTTGMRNTPAACGYFYITFVILVCWAVTLGFVIAAVAELPTMAALINPLFISLLILFCGLMQSPKAMPKFWSSWMYWLDPFHYYIEGLTVNELEDLKIVCTDDDLLRFSVPEGQTCGQYTADFFANGAPGYIANPDAVQPEQCGYCTYKSGPEFYEGNMGWSAANKWRNFGILFAFFIFNIIVFLGLVYWKRKGRR

>MambPDR7

MEEKKSALYAEQVEDAHLRDQPNYAHSSNSTIDADVSVNKENFGHNDTFGESEANQVNIEDAKNEYQELRRELSTKSQRLSLSKAEEGASNSEDFDLDQFLHGISKAQDENGHKRKHLGVAWRNLHVEGLGADAFTIPTVISNILSVVQFWKFFQKNKASTKVIIDELSGCCKDGEMLLVLGRPGAGCTSFLKVIANMRDSYTYIGGDVSYGGIDPKTFAERFRGQVCYNEEEDQHYPTLTTKQTLQFALRTKTPGKRVPGETKEDFVDRVLYMLGNMLGLTKQMNTMVGNAFVRGLSGGERKRLSIAEQMTTSSTINCWDCSTRGLDAASALDYVRSLRIMTDVFDITTIATLYQASNSIFNLFDKVLLLDEGRCIYFGPTSGARQYFDDLGFHCPPRKSLPDFLTGLCNPLEREFKPGFEDSAPKHAIEFQEKYYQSDVHKAMMRDLEDYEEKIAAENKANAFEDAVREEHQKRAPKKTPYIASFFQQVKALTIREHHLLIKDREALISRYGTMLIQGLITASCFFMLPLTGSGAFSRAGAIFFSAMFNTFISQSELVRFLTGRPILEKHKQYALYRPSAYYIAQVTMDIPYSLAQVLLYNICSYFMMGLNLTAGRFFTSFIILFFLTMCMTGFFRFFGAITSSFFMATQITGVLLIATTTYTGYTIPYDKMHPWLFWIYYINPITYAYKALVSNEMEGQLYSCEGAGNAVPFGPTYQDWNYKVCTMTGGNPGENFVRGDDYLRTALSYNPEDLWAPDFIVVVAFFIFFTILTALAMEYVKLNKAGSLTKLYLPGKAPKPRTTEEEDERRRQQDKITENMDKMSTGTTFSWQNVNYTVPIKGGSIQLLNNINGIVKPGHLCALMGSSGAGKTTLLDVLARRKTLGTVEGEIFLNNEALMTDFERITGYCEQMDVHQPAVTVREALRFSAYLRQPAEVPKEEKEEYVEQIIQLLEMTDIGDAQIGQVETGFGISVEERKRLTIGMELVGKPQLLFLDEPTSGLDAQSSFNIIRFIRKLADAGWPVLCTIHQPSAILFEHFDHLLLLVRGGRTAYYGEIGQDSRTMIDYFESNGGPKCSPDANPAEYILEVVGAGTAGKVTKDWAEVWQGSKECKALDTELDEIKATANKNPTREAHTYATSMTTQFRLVYGRMALAYWRSSDYNIGRFLNLMFTSLITGFTFWKLGASSSDMMYKIFALFGTFIMCFTMIILAQPKFMTERMYFRREYASSYYGWLPWGVSAILVEIPYVFFFAATFMFGFYWTTGMQNTASACGYFYITFVILVCWAVTLGFVIAAVAELPTMAAIINPLILSLLILFCGLMQSPKAMPKFWSSWMYWLDPFHYYIEGLTVNELENLKIVCTDGDLLRFSVPEGQTCGQYTADFFANGAPGYIANPDAVQPEQCGYCTYKSGPEFYEGNMGWSAANKWRNFGVLFAFFIFNIIVFLGLVYWKRKGRR

>McicPDR7

MEEKKNALYTEQVEDAHLRDQPNYAHSSNSTVDVDVNANKDNYGHNDTFGESEANQVNIEDAKNEYQELRRELSTKSQRLSLSKAEEGASNSEDFDLDQFLHGISKAQDENGHKRKHLGVAWRNLHVEGLGADAFTIPTLFSNILSVVQFWKFFQKSNASTKVIIDDLSGCCKDGEMLLVLGRPGAGCTSFLKVIANMRDSYTHIGGDVSYGGIDPKTFAERFRGQVCYNEEEDQHYPTLTTKQTLQFALRTKTPGKRIPGETKSDFVDRVLYMLGNMLGLTKQMNTMVGNAFVRGLSGGERKRLSIAEQMTTSSTINCWDCSTRGLDAASALDYVRSLRIMTDVFDITTIATLYQASNSIFNLFDKVLLLDEGRCIYFGPTSGAKQYFDDLGFHCPPRKSLPDFLTGLCNPLEREFKPGFEDSAPKHAIEFQEKYYQSDVHKAMMRDLENYEEKIAAENKANAFEDAVREEHQKRAPKKTPYIASFFQQVKALTIREHHLLIKDREALISRYGTMLIQGLITASCFYMLPLTGTGAFSRAGAIFFSAMFNTFISQSELVRFLMGRPILEKHKQYALYRPSAYYIAQVTMDIPYSLAQVLLYNICSYFMMGLNLTAGRFFTSFIILFFLTMCMTGFFRFFGAITSSFFMATQITGVLLIATTTYTGYTIPYQKMHPWLFWIYYINPITYAYKALVSNEMEGQQYSCEGAGNAVPFGPSYQDWNYKVCTMTGGNPGENFVRGDDYLRAALSYNPKDLWAPDFVVVVAFFIFFTILTALAMEYVKLNKAGSLTKLYLPGKAPKPRTTEEEDERRRQQDKITDNMDKMSTGTTFSWQHVNYTVPIKGGSIQLLNDINGIVKPGHLTALMGSSGAGKTTLLDVLARRKTLGTVEGDIFLNNEALMTDFERITGYCEQMDVHQPAVTVREALRFSAYLRQPAEVSKEEKDEYVEQIIQLLEMTDIGDAQIGQVETGFGISVEERKRLTIGMELVGKPQLLFLDEPTSGLDAQSSFNIIRFIRKLADAGWPVLCTIHQPSAILFEYFDHLLLLVRGGKTAYYGEIGQDSRTMIDYFESHGGPKCSPDANPAEYILEVVGAGTAGKVTKDWAEVWQNSEESKALNAELDEINATANKNPTREARTYSTSMTTQFRLVFGRMSLAYWRSSDYNIGRFLNLMFTSLITGFTFWKLGDSSSDMMYKIFALFGTFIMCFTMIILAQPKFMTERMYFRREYASSYYGWLPWGISAILVEIPYVFFFAATFMFGFYWTTGMQNTASACGYFYITFVILVCWAVTLGFVIAAVAELPTMAAIINPLILSLLILFCGLMQSPKAMPKFWSSWMYWLDPFHYYIEGLTVNELENLKIVCTEGDLLRFSVPEGQTCGQYTAEFFANGAPGYIANPDAVQPEQCGYCTYKSGPEFYEGNMGWSAANKWRNFGILFAFFIFNIIVFLGLVYWKRKGRR

>MracPDR7

MEEKKEVQYTERVEDAHLRDQPNYANSNNSTIDGDVSSSKDNAGHNDTFGESEANQVNIEDAKNEYQELRRELSTKSQRLSLSKAEEGASNSEDFDLDQFLHGISKEQDENGHKRKHLGVAWRNLHVEGLGADAFTIPTVISNILSVAQFWKFFQKKNSSTKVIIDDLTGCCKDGEMLLVLGRPGAGCTSFLKVISNMRDSYTHIGGDVSYGGIDPKTFAERYRGQVCYNEEEDQHYPTLTTKQTLQFALRTKTPGKRIPGETKSDFVDRVLYMLGNMLGLTKQMNTMVGNAFVRGLSGGERKRLSIAEQMTTSSTINCWDCSTRGLDAASALDYVRSLRIMTDVFDITSIATLYQASNSIFNLFDKVLLLDEGRCIYFGPTSGAKQYFDDLGFHCPPRKSLPDFLTGLCNPLEREFKPGFEDSAPKHASEFQAKFYQSDVHKAMLRDLEEYEEKIAAENKATAFEDAIHEEHQKRAPKKTPYIASFFQQVKALTIREHHLLIKDREALISRYGTMIIQGLITASCFFMLPLDGTGAFSRGGAIFFSAMFNTFISQSELVRFLMGRPILEKHKQYALYRPSAYYIAQVTMDIPYSLAQVFVYNICSYFMMGLNLTAGRFFTSFIILFFLTMCMTGFFRFFGAITSSFFMATQITGVLLIATTTYTGYTIPYQKMHPWLFWIYYINPITYAYKALISNEMEGQVYSCEGTGNAVPSGPGYDDWNYKVCTMAGGTAGENFVRGDDYLRSAFTYNTGDLWAPDFIVVVAFFIFFTIITALAMEYVKLNKSGSLTKLYLPGKAPKPRTTEEEDERRRQQDKVTENMDKISTGTTFSWQHVNYTVPIKGGSLQLLNDINGIVKPGHLTALMGSSGAGKTTLLDVLARRKTLGTVEGDIFLNNEALMTDFERITGYCEQMDVHQPAVTVREALRFSAYLRQPADVSKEEKEEYVEQIIQLLEMVDIGDAQIGQVESGFGISVEERKRLTIGMELVGKPQLLFLDEPTSGLDAQSSFNIIRFIRKLADAGWPVLCTIHQPSAILFEYFDHLLLLVRGGRTAYYGEIGQDSRTMIDYFESNGGPKCSPDANPAEYILEVVGAGTAGKASKDWAEVWQDSAESKALDAELDEINATANKNPTREAHTYSTSMGTQFRLVYGRMALAYWRSSDYNIGRFLNLMFTSLITGFTFWKLGDTSSDMMYKIFALFGTFIMCFTMIILAQPKFMTERMYFRREYASSYYGWLPWGVSAILVEIPYVFFFAATFMFGFYWTTGMQNTASACGYFYITFVILVCWAVTLGFVIAAVAELPTMAAIINPLILSLLILFCGLMQSPKAMPKFWSSWMYWLDPFHYYVEGLTVNELEHLEIVCTDADLLRFSPPPGQTCGEYTANFFSYGAPGYIANPNAVQPEQCGYCTYKSGAEFYETNMDWNAANKWRNFGILFAFFIFNIIIFLGLVYWKRKGRR

>MendPDR7

MDEKKVLDNQVEDTLPNHINNYPPSSASTINSGNRPNYGHQDTYGEAEANQVSIADAKSEYQDLRRELSRKSQRTSHSKAEEGQADSEDFNLDEFLHGISRQQDEEGRKRKHIGVSWKNLRVEGLGADAYTIPTVTSSLFRFLKVWKLFGKKNQSQKVILDNLTGCCKDGEMLLVLGRPGAGCTSFLKVISNMRDGYTHIGGDISYGGIDPKTFADRYQGQVCYNEEEDQHYPTLTTKQTLQFALRTKTPGKRPEGETKEDFVDRIIYMLGNMLGLTKQMNTLVGNAFVRGLSGGERKRLSIAEQMTTNSAVNCWDCSTRGLDAASALDYVRSLRIMTDVFEITTIATLYQASNSIFNLFDKVLVLDEGHCIYFGPTSGAKQYFDELGFHCPARKSTPDFLTGLCNPLEREFKPGFENSAPKHASEFQARFMESAVHKAMLQDLAEYEESIAKENKANEFENAVREEHQKRAGKSAPYIASFFQQVKALTIREHHLLIKDREALISRYGTMLIQGLITASCFFLIPLTGMGAFSRGGAIFFSAMFNTFISQSELVRFLMGRPILEKHKQYALYRPSAYYIAQVVMDIPYCVAQVLLYQICSYFMMGLNLTAGRFFTSSLILFFLTMCMTGFFRFFGAITSSFFLATQITGVLLIATTTYTGYTIPYNKMHPWLFWIYYINPITYAYKALISNEMEGQIYSCEGFGNSVPSGPEYTNWDYKVCTMAGGTSGEPFVRGSDYLRAAFSYNVEDLWAPDFVVVVAFFIFFTILTAMAMEWVKLKKSASLTKLYLPGKAPKPRTVEEEDERRRQQEKITQNMDKISTGTTFSWQHVNYTVPIKGGSLQLLNDINGIVKPGHLTALMGSSGAGKTTLLDVLARRKTIGTVEGDVFLNNEALMTDFERITGYCEQMDVHQPAVTVREALRFSAQLRQPADVSVEEKNEYVEQIIQLLEMDDIGDAQIGEVESGFGISIEERKRLTIGMELVGKPQLLFLDEPTSGLDAQSSYNIIRFIRKLANAGWPVLCTIHQPSAILFEHFDHLLLLVRGGRTAYYGEIGEDSRTMINYFESNGGPKCSPDANPAEYILEVVGAGTAGKATRDWADVWQNSEESKVLDAELNEINQTAVKNPTRNAQTYSTSYWTQFRLVFGRMSLAYWRSPDYNIGRFLNLMFTSLITGFTYWKLGTSSSDMMNKIFALFGTFIMCFTMIILAQPKFMTERMYFRREYASRYYSWFPWAVSAILVELPYIVFFSATFMFGFYWTAGMQNTAEACGYFYITFVALVCWAVTLGFVIAAVAELPTMAALINPLVLSILILFCGLMQSPFAMPKFWSSWMYWIDPFHYYIEGLTVNELGNLIVTCTQDDLLKFSPPPGQTCGEYTKAFFDLGATGYIANPDAVQPEQCGYCTYKTGAQFYETQMGWSLDNKWRNFGILVAFFIFNILLFIVLVFWKRKGRR

>MlanPDR7

MDEKRIEVLDEKPEDLSLQPQPKYINDDSSNSSTVDGNNNKNGYGHNGHFGESEADQVSIEGAKNEYESLRRELTSKSERISLSKAEDGQADSEDFNLDEFLHGISQEQNENGHKRKHLGVSWRNLRVEGLGADAFTIPTVISNVVKVLAFWRFFQKKRASTKIILDNLTGCCKDGEMLLVLGRPGAGCTSFLKVISNMRHAYTYIGGDVSYGGIDPETFSNRYQGQVCYNEEEDQHYPTLTTKQTLQFALRTKTPGKRIPGETKGDFVDRVLYMLGNMLGLTKQMNTMVGNAFVRGLSGGERKRLSIAEQMTTSSTINCWDCSTRGLDAASALDYVRSLRIMTDVFDITTIATLYQASNNIFNLFDKVLLLDDGHCIYYGPTSGAKPYFEGLGFHCPSRKSIPDFLTGLCNPLEREYKPGYENSVPKHASEFQDRYNQSDVCKTMLRDLEEYEEVLAKENKANVFEDAVREEHQKRAPKKTPYIASFFQQVKALTIREHHLLIKDRQALISRYGTMLIQGLITASCFYMLPMTGTGAFSRGGAIFFSAMFNTFIAQSELVRFLMGRPILEKHKQFALYRPSAFYCAQVIMDVPYCLAQVLLYNICSYFMMGLNLTAGKFFTSFLVLFFLTMCMTGFFRFFGAITSSFFLATQITGVLLIATTTYTGYTIPFQKMHPWLSWLYYINPITYSYKALISNEMEGQIYSCEGAGNAVPFGPGYDDWNYKVCTMEGGTPGEPFVRGNDYLRTALSYNAEDLWAPDFVVVVAFFIFFTFITAMAMEYVKLNKSATLTKLYLPGRAPKPRTTEEEDELRRQQDAITEKMDNMSTGTTFSWQHVNYTVPIKGGSIQLLNDISGIVKPGHLTALMGSSGAGKTTLLDVLARRKTLGTVSGDIFLNGEVLLNDFERITGYCEQMDVHQPAITVREALRFSAYLRQPAEVSKEEKDEYVEQTIQLLEMVDIGDAQIGEVESGYGISIEERKRLTIGMELVGKPQLLFLDEPTSGLDAQSSYNIIRFIRKLADAGWPVLCTIHQPSAILFEHFDHLLLLVRGGRTAYYGEIGQDSRTMIDYFESNGGPKCSPNANPAEYILEVVGAGTAGKAKRDWADVWEASPEAKALNTELDEIMQAADKNPTRDARTYAASLTTQFRLVYRRMSVAYWRSSEYNIGRFLNLMFTSLITGFTYWKLGVTSSDMMSKIFAMFGTFIMCFVLIILAQPKFMMERIYFRREYASRYYSWLPFGISAILVELPYIIFFSATFMFGFYWTAGMENTASACGYFYITFVVIVCWAVTLGFVIAAIAELPTMAALINPLVLSLLILFCGLMQSAKAMPKFWSSWMYWADPFHYYIEGLAVNELENLTVVCTEEDLLRFTPPPGQTCGEYTANFFAYGAPGYIANPDAAQPEQCGYCTYKSGAEFYETNLGWSASNKWRNFGILILFFMFNVILFLSLVYLRRKGRR

>MlusPDR8

MSNQKLELSDATPTAPPTRDVHQDAAKASSVSESQEEGTITDEGRYPNSGDMFVGNGDFGEIERDDINVEAAIDEFHELQKELSHISRRSSGMAPEKLEQGEASENDFNLSNFLHGMSDDRREAGHQLKHLGVIWKDLSVEGLGAEAFTIPTVISGLLKNIQFWKRFGVGATQSKKVILKNLSGFCKDGEMLLLLGRPGSGCTTLLRIISNMRGSFTAIKGTVSYGGFDHDTFAKRFHGQTCYNSEEDQHYPTLTTKQTLQFALRTKTPGTRLPNESKKEFINKILYLLGNMLGLTKQMQTMVGNPFFRGLSGGERKRLSIAEQMTTESTINCWDCSTRGLDAASALDYVRSLRIMTDVFKKTTVATLYQASNSIYNLFDKVILLDDGYCLYFGPVASAKQYFEELGFYCPPRKSTPDFLTGICNPLEREIREGYEDKVPVSGEQFQSVFYASPMYQSMMQELQAYEELIERERPSETFKEAMNQEHQKRASNRSPFIASYYQQVKALTIRQYHLLIKDMPALISRYGTILIQSLITASCFYNLPQDGSSSFSRGGALFFAVLFNALVSQTELVNFLMGRPILEKHKQYALYRPSAFYLAQVVMDIPYAIAQVLLFEICAYFLMGLKLTAGAFFTFFITLFFVNMCMNGFFRMFGAVTSSFFFATQFAGVIFISTITYCGYVIPYPDMHPWLYWIYWISPIAYGYKTLLINEMKGQEYSCEGAGNSVPYGPGYDLWDYKVCTMTGGHPGQNFVLGDDYLITKLQWDTRHLWAPDFVAVVGFFLLFTVLTALAMEFRAVNKIGSLTKLYIPGKAPKARSQEEIADQRRKQANKVETMEQISTGTNFSWQNVNYSVPIKGGEIQLLNSISGVVRPGHLCALMGSSGAGKTTLLDVLAKRKTIGKVDGRLYLNGEALINDFERITGYCEQMDIHQPAVTVREALQFSANLRQPYDTPQHEKDSYVEQIIHLLEMEDIADAQIGQVETGFGISVEERKRLTIGMELVGKPQLLFLDEPTSGLDAQSSFNIIRFIRKLADAGWPVLCTIHQPSAILFEHFDHLLLLVRGGRTAYYGEIGQDARTMIDYFEKNGGPKCSPAANPAEYILEVVGAGTAGKAKQDWAEVWEHSQEAKQLEQELEAVHQNADQNPQRHALTYATPMWNQFWLVHKRMALAYWRSPDYNIGRFTTIMFTSLLTGFTYWKLGSSVSDLQSRLFALFSSFLMANILIILAQPKFMTERLYFRREYASRYYGWIPFTISAILVEIPYILFLAAFFMFGFYWTAGLTNTSEAVGYFYLVLIFFVFWAVTLGFVIASVAENPTMAAVINPLVISMLILFAGFMQPEKSMPRFWSSWMYWLNPFHYFIEGLATNELSHINVRCTDKDLLKFLPPPNQTCRDYTNNFFSYGAPGYIDNPDAVQPELCGYCSFTSGEEFYSTTFGWSASHKWRNLGIIIAFFVFNVFCFGALVYWKRKGRR

>MambPDR8

MSNQKVELSSRDIHQEVVKASSISESQEEGTITDEGRYPNSGDGRMFVGNGDFGEIERDDINVEAAIDEFHELQKELSHISRRSSGMAPEKLEQGEASENDFNLSNFLHGMSDDRKEAGHQLKHLGVIWKNLSVEGLGAEAFTIPTVISGLLKNIQFWKRFGVGAKQSKKVILKNLSGFCKDGEMLLLLGRPGSGCTTLLRIISNMRSSFTDIKGTVSYGGFDHDTFAKRFHGQTCYNSEEDQHYPTLTTKQTLQFALRTKTPGTRLPNESKKEFINKILYLLGNMLGLTKQMDTMVGNPFFRGLSGGERKRLSIAEQMTTESTINCWDCSTRGLDAASALDYVRSLRIMTDVFKKTTVATLYQASNSIFNLFDKVILLDDGYCLYFGPVASAKQYFEELGFYCPPRKSIPDFLTGICNPLEREIREGYEDKVPVCGEQFQSVFYASPMYQSMMQELQAYEEMIEKEQPSETFKEAMNQEHQKRAPKRSPFIASFYQQVKALTIRQYHLLIKDMPALISRYGTILIQSLITASCFYNLPHDASSSFSRGGAIFFAVLFNALVSQTELVNFLMGRPILEKHKQYALYRPSAFYVAQVVMDIPYAIVQVLLFELCAYFLMGLNLTAGRFFTFFITLFFVNMCMNGFFRMFGAVTSSFFFATQFAGVIFISTITYCGYVIPYPDMHPWLYWIYWISPIAYGYKTLLINEMKGQEYSCEGAGNSVPYGPGYDLWDYKVCTMTGGHPGQNFVLGDDYLITKLQWDARHLWAPDFVAVVGFFLLFTVLTALAMEFRAVNKIGSLTKLYIPGKAPKARSQEEIADQRRKQANRVETMEQISTGTTFSWQNVNYSVPIKGGELQLLNSISGVVRPGNLTALMGSSGAGKTTLLDVLAKRKTIGKVDGRLYLNGEALINDFERITGYCEQMDIHQPAVTVREALQFSANLRQPYDTPQHEKDNYVEQIIHLLEMEDIADAQVGDVDHGFGISVEERKRLTIGMELVGKPQLLFLDEPTSGLDAQSSFNIIRFIRKLADAGWPVLCTIHQPSAILFDHFDHLLLLVRGGRTAYHGEIGEDARTMIDYFEKNGGPKCSPAANPAEYILEVVGAGTAGKAKQDWAEVWEHSQEAKQLEEELEAVHQNADQNPQRHALTYATPMWTQFWLVHKRMALAYWRSPDYNIGRFTTIMFTSLLTGFTYWKLGSSVSDLQSRLFALFSSFLMANILIILAQPKFMTERLYFRREYASRYYGWIPFTISVILVEIPYILFLAAFFMFGFYWTAGLTNTSEAVGYFYLVLIFFVFWAVTLGFVIASVAENPTMAAVVNPLVISMLILFAGFMQPEKAMPRFWSSWMYWLNPFHYFIEGLATNELSHINVHCTDKDLLKFLPPPNQTCRDYTNNFFSYGAPGYIDNPDAVQPELCGYCSFNSGEEFYSTTFGWSAAHKWRNLGIIIAFFVFNVFCFGALVYWKRKGRR

>McicPDR8

MSNQKLELSDNPTTTKHHHKASSVSESQEEGTITEGRYPNGGDGTLFVGNGDFGEIERDDINVEAAIDEFHELQKELSHISRRSSGMSPEKLEEGEASENDFNLSNFLHGMSDDRREAGHQLKHLGVIWKDLSVEGLGAEAFTIPTVISGLIKNIQFWKRFNVGVKPPKKVILKHLSGFCKDGEMLLLLGRPGSGCTTLLRIISNMRSSFTDVKGTVSYGGFDHETFAQRFHGQTCYNSEEDQHYPTLTTKQTLQFALRTKTPGTRLPNESKKEFINKILYLLGNMLGLTKQMDTMVGNPFFRGLSGGERKRLSIAEQMTTESTINCWDCSTRGLDAASALDYVRSLRIMTDVFKKTTVATLYQASNSIFNLFDKVILLDDGYCLYFGPVSAAKQYFEDLGFYCPPRKSTPDFLTGICNPLEREIREGYEDKVPVSGEQFQSVFYASPMYQSMMQELQAYEELIEKERPSETFKEAMNQEHQKRASNRSPFIASFYQQVKALTIRQYHLLIKDMPALISRYGTILIQSLITASCFYNLPTDGSSAFSRGGAIFFSLLFNALVSQTELVNYLMGRPVLEKHKQYALYRPSAYYVAQVVMDVPYAVVQVLLFELCAYFLMGLNLTAGRFFTFFIILFFVNMCMNGFFRMFGAFTSSFFFATQIAGVVFISTITYCGYVIPYPDMHPWLYWIYWISPIAYGYKALLINEMKGQEYSCEGAGNSVPYGPGYDLWDYKVCTMTGGHPGQNFVLGDDYLITKLQWDTKHLWAPDFIAVVGFFLLFTALTALAMEFRTVSKIGSLTKLYVPGKAPKARSQEEIADQRRKQANKVETMEQISTGTTFSWQNVNYSVPVKGGELQLLNSISGVVRPGNLTALMGSSGAGKTTLLDVLARRKTIGKVDGRLYLNGEALINDFERITGYCEQMDIHQPAVTVREALQFSANLRQPYDTPQHEKDSYVEQIIHLLEMEDIADAQVGEVDHGFGISVEERKRLTIGMELVGKPQLLFLDEPTSGLDAQSSFNIIRFIRKLADAGWPVLCTIHQPSAILFDHFDHLLLLVRGGRTAYHGEIGEDARIMIDYFEKNGGPKCSPAANPAEYILEVVGAGTAGKAKQDWAEVWEHSEEAKQLEQELESVHQNADQNPQRHALTYATPMWTQFWLVHKRMALAYWRSPDYNIGRFTTIMFTSLLTGFTYWKLGSSISDLQSRLFALFSSFLMANILIILAQPKFMTERLYFRREYASRFYGWIPFTISVILVEIPYILFLAAFFMFGFYWTAGLTNTSEAVGYFYLVLIFFVFWAVTLGFVIASVAENPTMAAVVNPLVISMLILFAGFMQPEKAMPRFWSSWMYWLNPFHYFIEGLATNELSHINVRCTDKDLLKFLPPPNQTCRDYTNNFFSYGAPGYIDNPDAVQPELCGYCSFNSGEEFYTTTFGWSAAHKWRNLGIIIAFFVFNVFCFGALVYWKRKGRR

>MracPDR8

MSNQKLQLSDALEDQKEAARASSISESQEEGTIIDTGHHGGAAAGEVFVGNGEFGETERDGIDVAAAIDEFHELQKELSHISRRSSGIVPEKLEEGDANENDFNLSSFLHGMSDDRKEAGHKFKHLGVIWKDLTVEGLGADAHTIPTVISGLIKNIQIWKRFGVGANTTKKVILKDLSGFCKDGEMLLLLGRPGAGCTSLLKIISNMRSSFTDIKGTVSYGGFDHIKFAKKFHGQTCYNSEEDQHYPTLTTKQTLQFALRTKTPGTRLPNESKKEFINKILYLLGNMLGLTKQMQTMVGNPFVRGLSGGERKRLSIAEQMTTESTINCWDCSTRGLDAASALDYVRSLRIMTDVFKKTTVATLYQASNNIYNLFDKVMLLDDGYCLYFGPVTSAKKYFEDLGFYCPPRKSIPDFLTGICNPLEREIREGFEGNVPESGEQFQSAFYASSIYQTMMQELQAYEELIEKERPSETFKEAMNQEHQKRAPKGSPFIASFYQQVKALTIRQFHLLIKDKPALISRYGTILIQSLITASCFYNIPNDGSGAFSRGGAIFFSVLFNALVSQTELVNFLMGRPILEKHKQYALYRPSAFYVAQVVMDVPYALVQVLLFEICVYFIMDLNLTAGRFFTFFITLFFVNMCMNGFFRMFGAITSSFFFATQFAGVIFISTITYCGYVIPYPDMHPWLYWIYWISPIAYGYKTLLINEMKGQEYSCEGAGNSVPYGPGYDIWDYKVCTMAGGHAGQNFVLGDDYLITKLQWDTKHLWAPDFVAVVGFFLLFTLLTALAMEFGAVNKIGSLTKLYLPGKAPKARSEEEIAAQRRKQVNKVETMEAISSGTTFSWQNVNYTVPIKGGEIQLLNSISGVVRPGHLTALMGSSGAGKTTLLDVLARRKTLGKVDGRLYLNGEVLINDFERITGYCEQMDIHQPAVTVREALQFSANLRQPYETPQSEKDSYVEQIIHLLEMEDIADAQVGDVDRGFGISVEERKRLTIGMELVGKPQLLFLDEPTSGLDAQSSFNIIRFIRKLADAGWPVLCTIHQPSAILFDHFDHLLLLVRGGRTAYHGEIGEDARTMINYFEKNGGPVCSPAANPAEYILEVVGAGTAGKAKQDWAEIWEHSEEAKQLEQELETVHQNADQNPQRHALTYATPIWTQFLLVHKRMALAYWRSPDYNIGRFTTIMFTSLLTGFTYWKLSNSVSDLQNRLFALFSSFLMANILIILAQPKFMTERLYFRREYASRFYGWIPFTISVILVEIPYILFLAAFFTFGFYWTAGLTNTSDAVGYFYLVLIFFVFWAVTLGFVIASVAENPTLAAVVNPLVISMLILFAGFLQPVKAMPHFWSSWMYWLNPFHYFIEGLATNELSHINVHCSDRDLLKFLPPPGQTCENYTSNFFSSGATGYIDNPQAVQPELCGYCSFSSGEEYYTTTFGWSVGNRWRNLGIIIAFFVFNIFCFGAIVYWKRKGRR

>MendPDR8

MTGSSESISYQKEIKTTTTKGSSISESQEEGTIIETITLPNNEVFVSGDGNFGEGSADEVNVGAAEQEYSELKKVLSHLSRISTRKSEKELEEGAVEENDFNLSDFLHGMSNDQKEAGHEPKHLGVVWKNLTVEGLGAEAHTIPTVISGVIKNLQFWKFFGIGVKKSTKVILKDLSGFCKDGEMLLLLGRPGAGCTTLLKIISNMRGSFTAVKGEVSYGGYDHDHFAKKFRGQTCYNSEEDQHYPTLTLKQTLQFTLRTKTPGTRLPNETKKDFINKILYMLGNMLGLTKQMDTMVGNAFVRGLSGGERKRLSIAEQMTTQSTINCWDCSTRGLDAASALDFVRSLRIMTDVFKKTTVASLYQASNNIFNLFDKILLLDDGYCLYFGPASTAKQYFENLGFLCPPRKSTPDFLTGLCNPIEREIKPGFENSVPVFAIDFQQRYYESSIYREMMEEMSAYEQVIEKETPAENFKVAMDQEHQKRAPNGSPFIASFYQQVIALTIRQYHLLIKDMPALISRYGTILIQALIMASCFYNIPNDGSGAFSRGGAIFFSVLFNAFISQSELINFLMGRPILEKHKQYALYRPSAFYVAQVIMDIPYALVQVLLFQICSYFMMGLSLDAGRFFTFFVALFFVNMCMNGFFRFFGAITSSFFFATQISGVLLIATITYCGYVIPYGDMHPWLSWIYWISPIAYSYKTLLVNEMKGQKYSCEGAANSVPSGPGYDNWDYKVCTMAGGNPGEDFVLGDNYLKAALQWDTKDLWAPNFTVIVAFFILFTVLTALAMEFGAISKVGSLTKLYIPGKAPKPRTPEEEEARRRKQASQDETVEAISAGTTFSWQDVDYSVPIKGGDLQLLNKISGIVRPGHLTALMGSSGAGKTTLLDVLARRKTLGKVSGRVYLNGEALINDFERITGYCEQMDIHQPAVTVREALQFSANLRQPAETPQAEKDAYVEQIIQLLEMEDIADAQVGEVDHGFGISVEERKRLTIGMELVGKPQLLFLDEPTSGLDAQSSFNIIRFIRKLADAGWPVLCTIHQPSAILFDHFDHLLLLVRGGRTAYHGPIGEDARTMIDYFEKNGGPICSPAANPAEYILEVVGAGTAGKAKKDWADVWANSKEAKELANELEEVHENADHNPQRKALTYATSFTTQFYLVHKRMALAYWRSPDYNIGRFITIMFTSLITGFTYWKMSNSVSDLKNRLFALFASFIMANILIILAQPKFMTERLYFRREYASRYYGWVPFTISVILVEIPYILVLSAFFMCGFYWTAGLTNVSEAVGYFYLMLIFFVFWAVTLGFVIASVAENPTMAGVVNPLVISLFILFAGLMQPVKAMPRFWSSWMYWLNPFHYFIEGLAVNELHYITTTNCIDKDLVKFLPPPGQNCGDYTKNYFSYGAPGYIVNPQAVQPELCGYCSFKSGAEFYETSFSWDVANKWRNLGIVIAFFVFNVFCFGALVYWNRKGRR

>MlanPDR8

MAESNYSNKNEAMKGSSISESQEEGTVVMTSRSEEIFVGHGDFGEHDRNEINVEAAIDEFHELKKELSHISHRSFVDPAKAEEGTADEEDFNLNEFLHGMSHKQKEAGHQLKHLGVVWKNLSVEGLGADAYYIPTVVSGLIRTLQFWKLFKWGSKASTKVILKDLSGFCKDGEMLLLLGRPGAGCTTLLKIISNMRGSFTNVKGTVSYGGFDQHTFSRRFRGQTCYNSEEDQHYPTLTAKQTLKFALRTKAPGTRLPNETRKEFINKILFMLGNMLGLTNQMNTMVGNHFVRGLSGGERKRLSIAEQMTTQSTINCWDCSTRGLDAASALDYVRSLRIMTDVFQKTTVATLYQASNDIYNLFDKLLLLDDGYCLYFGPISDAKQYFEDLGFYCPPRKSTPDFLTGICNPLEREIKPGFENSVPTCGNQFQERFYMSSIYTAMMTELDAYEHATQNEKTVDTFKEAMDQEHQKRASNQSPFISSYFQQVIALTIRQYHLLIKDKPALISRYGTILIQSLITASCFYNIPHDGSGAFSRGGALFFAVMFNALVSQSELVNFLMGRPILEKHKQYALYRPSAFYLARVVMDVPYAFVQVLLFEICAYFMMGLNLSAGRFFTFFITLFFVNMCMNGFFRMFGAVTSSFFFATQFAGIIMVSTVMYCGYVIPYGDMHPWLSWIYWISPIAYGYKTLLANEMEGLKYTCEGIGNSVPSGPGYDDWNYKVCTMAGSLPGENFVLGDNYLKAKFQWDVKDLWVPDFVAVVGLFLLFTVVTAAVMEFGGASKTGSLTKLYIPGKAPKQRTQEEDAEHRRNQASRVDTVEAISSGTTFSWQKVNYSVPIKGGDLKLLDNISGIVRPGHLTALMGSSGAGKTTLLDVLSRRKTLGKIDGRLYLNGEALINDFERITGYCEQMDVHQPAVTVRESLQFSARLRQPHEVPQAEKDDYVEQIIQLLEMEDIADAQVGSVESGFGISVEERKRLTIGMELVAKPQLLFLDEPTSGLDAQSSYNIIRFIRKLADAGWPVLCTIHQPSSILFSHFDHLLLLVRGGRTAYHGPIGEDARTMINYFQDNGGPICSPAANPAEYILEVVGAGTAGKAKQDWAEIWTNSSESMQLEEELEQVHQSADQNPQRHALTYAAPLPTQLYYVYKRMALAYWRSPDYNIGRFITIMFNSLLTGFTYWKMTNTVSDLQNRLFCLFATFLMANVLIILAQPKFMTERLYFRREYAGRFYSWIPFTISVILVEIPYILFLSAFFMCGFYWTAGLSNISEAVGYFYLMTIFFVMWAVTLGFLIASVAEIPTMAAVVNPLVISMLILFAGLMQPEKSMPHFWSAWMYWLNPFHYFIEGLAVNELSHIQVHCSDKDLIKFFPPPGLTCGDYTRNFFSNGAPGYIDNPQATQPEICGYCSFSNGQEFYSTSFGWDVAHKYRNLGIIIAFFIFNVFCFGALVYWKRKGRR

**Following is the FASTA file of select representative fungal PDR transporters that were used to analyse the phylogenetic relationship of *Mucor* PDR transporters with the rest of representative full-size fungal PDR transporters. These sequences were used to create Figure 3 and Figure S4.**

>ScPDR5

MPEAKLNNNVNDVTSYSSASSSTENAADLHNYNGFDEHTEARIQKLARTLTAQSMQNSTQSAPNKSDAQSIFSSGVEGVNPIFSDPEAPGYDPKLDPNSENFSSAAWVKNMAHLSAADPDFYKPYSLGCAWKNLSASGASADVAYQSTVVNIPYKILKSGLRKFQRSKETNTFQILKPMDGCLNPGELLVVLGRPGSGCTTLLKSISSNTHGFDLGADTKISYSGYSGDDIKKHFRGEVVYNAEADVHLPHLTVFETLVTVARLKTPQNRIKGVDRESYANHLAEVAMATYGLSHTRNTKVGNDIVRGVSGGERKRVSIAEVSICGSKFQCWDNATRGLDSATALEFIRALKTQADISNTSATVAIYQCSQDAYDLFNKVCVLDDGYQIYYGPADKAKKYFEDMGYVCPSRQTTADFLTSVTSPSERTLNKDMLKKGIHIPQTPKEMNDYWVKSPNYKELMKEVDQRLLNDDEASREAIKEAHIAKQSKRARPSSPYTVSYMMQVKYLLIRNMWRLRNNIGFTLFMILGNCSMALILGSMFFKIMKKGDTSTFYFRGSAMFFAILFNAFSSLLEIFSLYEARPITEKHRTYSLYHPSADAFASVLSEIPSKLIIAVCFNIIFYFLVDFRRNGGVFFFYLLINIVAVFSMSHLFRCVGSLTKTLSEAMVPASMLLLALSMYTGFAIPKKKILRWSKWIWYINPLAYLFESLLINEFHGIKFPCAEYVPRGPAYANISSTESVCTVVGAVPGQDYVLGDDFIRGTYQYYHKDKWRGFGIGMAYVVFFFFVYLFLCEYNEGAKQKGEILVFPRSIVKRMKKRGVLTEKNANDPENVGERSDLSSDRKMLQESSEEESDTYGEIGLSKSEAIFHWRNLCYEVQIKAETRRILNNVDGWVKPGTLTALMGASGAGKTTLLDCLAERVTMGVITGDILVNGIPRDKSFPRSIGYCQQQDLHLKTATVRESLRFSAYLRQPAEVSIEEKNRYVEEVIKILEMEKYADAVVGVAGEGLNVEQRKRLTIGVELTAKPKLLVFLDEPTSGLDSQTAWSICQLMKKLANHGQAILCTIHQPSAILMQEFDRLLFMQRGGKTVYFGDLGEGCKTMIDYFESHGAHKCPADANPAEWMLEVVGAAPGSHANQDYYEVWRNSEEYRAVQSELDWMERELPKKGSITAAEDKHEFSQSIIYQTKLVSIRLFQQYWRSPDYLWSKFILTIFNQLFIGFTFFKAGTSLQGLQNQMLAVFMFTVIFNPILQQYLPSFVQQRDLYEARERPSRTFSWISFIFAQIFVEVPWNILAGTIAYFIYYYPIGFYSNASAAGQLHERGALFWLFSCAFYVYVGSMGLLVISFNQVAESAANLASLLFTMSLSFCGVMTTPSAMPRFWIFMYRVSPLTYFIQALLAVGVANVDVKCADYELLEFTPPSGMTCGQYMEPYLQLAKTGYLTDENATDTCSFCQISTTNDYLANVNSFYSERWRNYGIFICYIAFNYIAGVFFYWLARVPKKNGKLSKK

>CgCDR1

MSLASDKKDADVASTTTTAQDDDNLSTYHGFDHHVQDQVRQLARTLTQQSSLHQKKEHTLPEEGINPIFTNTEADDYNPRLDPTSDEFSSAEWVQNMSNISNSDPDYYKPYSLGCYWKDLVATGESADIEYQANFLNGPYKGLKTVYNTVVPSTASSKDKNFKILKSMEGAVNPGELLVVLGRPGSGCTTLLKSISSNTHGFNIAKESTISYSGMTPNDIRKHFRGEVVYNAEADIHLPHLTVYQTLLTVARLKTPQNRLKGIDRETYARHLTEVAMATFGLSHTRNTKVGNDLVRGVSGGERKRVSIAEVSICGSKFQCWDNATRGLDSATALEFIRALKVQASISNAAATVAIYQCSQDAYDLFDKVCVLYDGYQIYFGPAGKAKEYFQKMGYVSPERQTTADFLTAVTSPSERIINQDYINRGIFVPQTPKEMWEYWRASEDHADLIKEIDSKLSDNYDANLAEIKDAHVARQSKRARPSSPYTVSYGMQIKYLLIRNFWRIKQSSGVTLFMVIGNSSMAFILGSMFYKVMKHNTTSTFYFRGAAMFFAVLFNAFSSLLEIFSLFEARPITEKHRTYSLYHPSADAFASILSEVPAKLITAVCFNIIYYFLVNFRRNGGVFFFYFLINIVAVFAMSHLFRCVGSVSKTLSAAMVPASMLLLGLSMYSGFAIPRTKILGWSKWIWYINPLAYLFESLMINEFHDRKFPCSQYIPSGSVYNNVPADSRICSSVGAIRGNDYVLGDDFLRESYSYLHKHKWRGFGIGLAYVIFFLVLYLILCEYNEGAKQKGEILVFPQNIVRRMKKERKLKNVSSDNDVEIGDVSDISDKKILADSSDESEESGANIGLSQSEAIFHWRNLCYDVQIKKETRRILNNVDGWVKPGTLTALMGASGAGKTTLLDCLAERVTMGVITGEVSVDGKQRDDSFARSIGYCQQQDLHLKTSTVRESLRFSAYLRQPADVSIEEKNQYVEDVIKILEMEQYADAVVGVPGEGLNVEQRKRLTIGVELAAKPKLLVFLDEPTSGLDSQTAWSICQLMKKLANHGQAILCTIHQPSAILMQEFDRLLFLQRGGKTVYFGDLGDGCKTMIDYFESHGSHKCPPDANPAEWMLEVVGAAPGSHANQDYHEVWRNSDEYQKVQEELEWMSNELPKKNTNNSETVHKEFATGVLYQCKLVSLRLFQQYWRSPDYLWSKFFLTIFNNIFIGFTFFKADRSLQGLQNQMLAVFMFTVIFNPLLQQYLPSFVQQRDLYEARERPSRTFSWKAFIVSQILVEIPWNILAGTVAFVIYYYAIGFYSNASVAHQLHERGALFWLFSCAFYVYIGSLALFCISFNQVAEAAANMASLMFTLSLSFCGVLVTPNGMPRFWIFMYRVSPLTYLIDGMLSTGVANVAIKCSNYELLRFSPAANLTCGEYLGPYLQTVKTGYIVDPSATDTCELCPYSHTNDFLSSVSSKYSRRWRNWGIFICYIAFNYIAGIFLYWLARVPKKSGKLAKK

>CkABC1

METISVGKESDVSSDGNVAYGGFNEGTNKQIRDLAREFTRQTSVGDGVQDSDDATDSNEVEKFGVDHASPNSPPKSNQYDLLRTLTSMSQVPGVNPVDQTIDPRLDPNSDEFESKFWVKNMRKLLDSDPDYYRPTSLGFAAKNLIAKGISSDADYQANFLNYPFKVVRDTYMDLFRGNDESRYFEILKSMDVLIKPGTLTVVLGRPGAGCSTFLKTVAAQTYGFKVDDSSIISYDGLTPKEINKNYRGEVIFSAEMDNHFPHLSVGQTLEFAAKMRTPQNRFPGVSRNEYAKHMSEVYMATYGLSHTVNTKVGDNFIRGVSGGERKRVSIAEASLCGANLQCWDNATRGLDAATALEFVRALKTSAHILDTTPLIAIYQCSQDAYDLFDNVVLLYEGYQIYFGPGDRAKDFFERMGYECPDRQTTADFLTSITSPAERVAKKGWENKVPQTPKEFSDYWRASAEYKELVADIDEYLSHCHNNNTREEFAEAHAIKQANHARPSSSFRVSYWMQIKLIAQRNIWRTKGDPSIMMFSVIANIIMGLIISSLFYNLSATTGTFYYRSAAMFFAVLFNAFSSLLEVMSLFESRPIVEKHKMFALYHPSADAFASIFTELPAKIATSLGFNLMFYFMVNFRRNPGRFFFYLLMNFMATLVMSHIFRSIGSCFKTLSESMPPATVFLTAMVIYTGFALPTPSMHGWSRWINYLDPVAYVFEALMANEFDGRRFECSQFIPSYPNADLANQVCSVVASVPGFSYVNGTDYIYESYRYKITHKWRNFGIVVGFIIFFLFVYVTLVELNKGAMQKGEIILFQQSKLREMRKEKKSKQISDIEGGSEKPAGVYDHGNEDSEDGVNNLTVGSDIFHWRDVCYEVQIKDETRRILNHVDGWVKPGTLTALMGASGAGKTTLLDVLANRVTMGVVSGSMFVNGRLRDQSFQRSTGYVQQQDLHLQTSTVREALRFSAYLRQSRTISKKEKDEYVESIIDILEMRSYADAVVGVAGEGLNVEQRKRLTIGVELAAKPKLLLFLDEPTSGLDSQTAWSVCQLMRKLADHGQAILCTIHQPSALLLKEFDRLLFLAKGGRTVYFGDLGENCQTLINYFESHGAHPCPAEANPAEWMLEVIGAAPGSHANQDYHEVWMSSDERRAVQEELHRMETELLQIPVDDSAEAKRSFASSYLIQYICVTKRVLQQYYRTPQYIWSKLFLAGANSIFNGFSFYRAGTSLQGLQNQMLSIFMLSVMLNTLVQQMLPLYITQRSIYEVRERPSKTFSWWVFLAAQVTAEFPWNLICGTISYFCWYYPIGLQNNASVTHTTAERGALTWLLIVGFFNYASSLGLMCIAGVEQEQNGANISNLLFTMCLNFCGILKYPTGFWKFMYRANPFTFWIASVLGAGVGDTPLVCSSKEIVYFAPPKGETCTTYIQPYIDEAGGYLVDSEREGYCGFCTASNTNAYLKSVHVEYSKRWQNWGIFICFIAINNIFMFLFYWLARVPKKDNRVKDASALKSDEKATLGPQTTSEA

>CaurCDR1

MSEKPFVDAPPPEDGVAHQVSPHDNGSLSEEANSINEYTGFGAHQEGEIRELARTFTNMSHDSGHDLSKTNTSQDLLKYLSHMSEVPGVEPFDPEQISEQLNPDSPNFNAKFWVKNMRKLFDSNPDYYKPSKLGLAYRNLRAYGVAADSDYQPTVSNGLWKMAVDYWHDMRKIDESRCFDILKTMDGYFKPGEVTVVLGRPGSGCSTLLKTIACNTYGFHIGEESQISYDGMTPDEIHKHHRGDVVYSAETDVHFPHLSVGDTLEFAAKLRTPQNRGEVSRLEHAKHMASVTMATYGLSHTRNTPVGNDFVRGVSGGERKRVSIAEVSLSGANIQCWDNATRGLDAATALEFIRALKTSAAILDATPLIAIYQCSQDAYDLFDNVIVLYEGYQIFFGKASEAKQFFLDMGYECPQRQTTADFLTSLTNPEERVVKPGFENKVPRTAKEFSDYWRNSSNYKVLTAGIDKYLAEVADGSQREAYRASHVAKQSDHTRPSSPYTVSFFMQTRYIIGRNFLRMKGDPSIVIFSIFGQGVMGLILSSVFYNLQPTTGSFYYRGAAMFFAVLFNAFASLLEIMSLFEARPIVEKHKKYALYRPSADALASIISELPVKLCMSTCFNFSFYFMVHFRRDPGRFFFYWLFCGLCTLCMSHMFRSLGAVSTSLAAAMTPATSVLLAMVIFTGFVIPIPSMLGWCRWIQYINPVSYVFDSLMVNEFHGRKFECAQFVPSGGPYDQVAAVNRVCSTAGARPGEDFVDGTAYLQTSFEYVNAHKWRNLGIVVAYIVVFLGVYIALTEFNKGAMQKGEIALFLRGSLKKVRKQREQNEAKVNDVENNLPNEKISYSDAMEKDSGESSTSDDKLPNQRQIFHWKDLTYQVKIKAENRVILNHVDGWVKPGQITALMGASGAGKTTLLNCLSERLTTGTVTDGVRMVNGHGLDSSFQRSIGYVQQQDIHLATSTVREALTFSAYLRQPSHVSKKEKDEYVDYVIDLLEMGAYSDALVGVAGEGLNVEQRKRLTIGVELVAKPKLLLFLDEPTSGLDSQTAWSICKLMRKLANHGQAILCTIHQPSAILLQEFDRLLFLQKGGKTVYFGDLGKNCQGLIDYFEKHGAHPCPPDANPAEWMLEVVGAAPGSKAAQDYFEVWRNSEEYQEVQRELAYMENELGKLPVDEDPESRKKYATSLIKQYFIVTWRTFQQYWRSPGYIYSKFFLVITASLFNGFAFFHSGTSQQGLQNQMFSMFMFYMPLQTLIQQMLPYYVMQREIYEVREAPSRTFSWFAFIASQITTEIPFQVVLGTVAFFCWYYPVGLYQNATPTDTVHERGALMWLLVTAFYVYTISLGQMVVAFMEIADNAANMVNLMFIMCLNFCGVLATPEALPGFWIFMYRCNPFTYLIQAMLSTGLANTKIVCSSREILHFQPPSGQTCGQYMQQFISAAGGYLLDESATDQCDFCAMSQTNTFLDSVHAVYSERWRNFGIFIAFIAINMIGTIFFYWLARVPKSSKSKNH

>CaCDR1

MSDSKMSSQDESKLEKAISQDSSSENHSINEYHGFDAHTSENIQNLARTFTHDSFKDDSSAGLLKYLTHMSEVPGVNPYEHEEINNDQLNPDSENFNAKFWVKNLRKLFESDPEYYKPSKLGIGYRNLRAYGVANDSDYQPTVTNALWKLATEGFRHFQKDDDSRYFDILKSMDAIMRPGELTVVLGRPGAGCSTLLKTIAVNTYGFHIGKESQITYDGLSPHDIERHYRGDVIYSAETDVHFPHLSVGDTLEFAARLRTPQNRGEGIDRETYAKHMASVYMATYGLSHTRNTNVGNDFVRGVSGGERKRVSIAEASLSGANIQCWDNATRGLDSATALEFIRALKTSAVILDTTPLIAIYQCSQDAYDLFDKVVVLYEGYQIFFGKATKAKEYFEKMGWKCPQRQTTADFLTSLTNPAEREPLPGYEDKVPRTAQEFETYWKNSPEYAELTKEIDEYFVECERSNTRETYRESHVAKQSNNTRPASPYTVSFFMQVRYGVARNFLRMKGDPSIPIFSVFGQLVMGLILSSVFYNLSQTTGSFYYRGAAMFFAVLFNAFSSLLEIMSLFEARPIVEKHKKYALYRPSADALASIISELPVKLAMSMSFNFVFYFMVNFRRNPGRFFFYWLMCIWCTFVMSHLFRSIGAVSTSISGAMTPATVLLLAMVIYTGFVIPTPSMLGWSRWINYINPVGYVFESLMVNEFHGREFQCAQYVPSGPGYENISRSNQVCTAVGSVPGNEMVSGTNYLAGAYQYYNSHKWRNLGITIGFAVFFLAIYIALTEFNKGAMQKGEIVLFLKGSLKKHKRKTAASNKGDIEAGPVAGKLDYQDEAEAVNNEKFTEKGSTGSVDFPENREIFFWRDLTYQVKIKKEDRVILDHVDGWVKPGQITALMGASGAGKTTLLNCLSERVTTGIITDGERLVNGHALDSSFQRSIGYVQQQDVHLPTSTVREALQFSAYLRQSNKISKKEKDDYVDYVIDLLEMTDYADALVGVAGEGLNVEQRKRLTIGVELVAKPKLLLFLDEPTSGLDSQTAWSICKLMRKLADHGQAILCTIHQPSALIMAEFDRLLFLQKGGRTAYFGELGENCQTMINYFEKYGADPCPKEANPAEWMLQVVGAAPGSHAKQDYFEVWRNSSEYQAVREEINRMEAELSKLPRDNDPEALLKYAAPLWKQYLLVSWRTIVQDWRSPGYIYSKIFLVVSAALFNGFSFFKAKNNMQGLQNQMFSVFMFFIPFNTLVQQMLPYFVKQRDVYEVREAPSRTFSWFAFIAGQITSEIPYQVAVGTIAFFCWYYPLGLYNNATPTDSVNPRGVLMWMLVTAFYVYTATMGQLCMSFSELADNAANLATLLFTMCLNFCGVLAGPDVLPGFWIFMYRCNPFTYLVQAMLSTGLANTFVKCAEREYVSVKPPNGESCSTYLDPYIKFAGGYFETRNDGSCAFCQMSSTNTFLKSVNSLYSERWRNFGIFIAFIAINIILTVIFYWLARVPKGNREKKNKK

>CneAFR2

MAFAGVGPMGPYDRTEHSAGAALNRTTSRSHYYDSHPNDNLPSPTDEYRNRELGQLARSWSRRSQTGAVGRGLSQAPQDESISEELSGDVFAYEQGSDLDPFSNNFDAKKWTKLMFHAHEATTPSRKAGLSFKNLGVFGYGSDADYQKTVGNLFLAGLGALRDLIGNRKRKVQILNGIDGVLEAGEMLVVLGPPGSGCSTMLKTIAGEMNGIYIDESSELNYRGITPKQMYGQFRGEAIYTAEVDVHFPNLTVGQTLSFAAEARAPRHTPNGISKKDYANHLRDVVMSIFGISHTLNTIVGNDFVRGVSGGERKRVTIAEAALAGAPLQCWDNSTRGLDSANAIEFCKNLRINADYMDISSVVAIYQAPQSAYDLFDKVSVLYEGEQIFFGKCNDAKQFFMDMGFHCPPQQTVPDFLTSLTSASERTPREGFEGKVPTTPQEFAAAWKKSDKYAELQEQIAQFEQKYPVNGENYNKFLESRRAQQSKHLRPKSPYTLSYGGQIKLCLRRGFQRLKADPSLTLTQLFGNFIMALIVGSVFYNMPENTSSFYSRGALLFFAILMSAFGSALEILILYAQRGIVEKHSRYAFYHPSAEAVASALTDIPYKVLNCICFNLALYFMANLRREPGPFFFFMLISFTLTMVMSMFFRSIASLSRSLTQALAPAAIMILALVIYTGFAINVQNMRGWARWINYLDPIAYGFESLMINEFHGREYACSAFVPTGPGYEGATGEERVCSTVGSVAGSPVVNGDAYINGSYEYYHAHKWRNFGILIGFFIFLTAVYLLATELITAKKSKGEILVFPRGKIPRALLAQSTVSHNSDDPEAGKYAGGGNVQKKVTGADRADAGIIQRQTAIFSWKDVVYDIKIKKEQRRILDHVDGWVKPGTLTALMGVSGAGKTTLLDVLATRVTMGVVTGEMLVDGQQRDISFQRKTGYVQQQDLHLETSTVREALRFSVLLRQPNYVSKKEKYEYVEEVLKLLEMDAYADAVVGVPGTGLNVEQRKRLTIGVELVAKPALLLFLDEPTSGLDSQTSWNILLLLRKLTEHGQAILCTIHQPSAMLFEQFDRLLFLAKGGKTVYFGEVGKESRTLINYFERNGAEKCPPGENPAEWMLSAIGASPGSHSTVDWHQTWLNSPDREEVRREIARIKETNGGKGKAAEQDKSREKSKAEIKAEYAEFAAPLWKQFIIVVWRVWQQHWRTPSYIWAKAALCIGSGLFIGFSFFKSGTSQQGLQNQLFSVFMLFTIFGQLVQQILPNFVTQRSLYEVRERPSKTYSWKVFIMSNVIAEIPWSILMGVIIYFTWYYPIGYYRNAIPTDSVHLRGALMFLYIEMFMLFTSTFAIMIVAGIDTAETAGNIANLLFLMCLIFCGVLATKDSFPHFWIFMYRVSPFTYLVEGMLGVAIANTNVVCADNELLSFNPPSGQTCGQYMSNYIAAAGGYLINEDATIGCSFCSVTIMIGAVLIFVSIFRWRDFGLLWIFVIFNAAAAVAIYYVARVPKNTGKEQASEPEEPEKNAAPGNTEKRSRRSESS

>AfuCdr1B

MSLLGTINPNINPERTVAGRGTQEEEGEIARVEHNHHNNAASVSTDETVLERSKEIGDEDVAVEEVTRLARQLTRQSTRFSTSGNVENPFLETKEDSTLNPLSPNFKAKNWMKNLLALSSRDPERYPKRVAGVAFKNLSVHGYGSPTDYQKDVFNSVLEVGTLVRRIMGTGKQKIQILRDFDGLVKSGEMLVVLGRPGSGCSTFLKTISGEMNGIYMDEKSYLNYQGISSKQMRKQFRGEAIYTAETDVHFPQLTVGDTLKFAALARAPRNRLPGVSREQYAVHMRDVVMAMLGLTHTMNTRVGNDFVRGVSGGERKRVSIAEATLSGSPLQCWDNSTRGLDSANALEFCKTLNLMTKYAGATVAVAIYQASQSAYDVFDKVTVLYEGRQIYFGRTDEAKEFFTNMGFECPERQTTADFLTSLTSPAERVVKPGFEGKVPQTPDEFVRAWKSSEAYAKLMREIEEYDREFPIGGESLNQFIESRRAMQAKNQRVKSPYTISVWQQIELCMIRGFQRLKGDSSLTMSQLIGNFIMALVIGSVFYNLPDDTSSFYARGALLFFAVLLNAFSSALEILTLYAQRPIVEKQARYAMYHPFAEAIASMLCDMPYKITNAIIFNLTLYFMTNLRREPGAFFVFLLFSFVTTLTMSMLFRTMAASSRTLSQALVPAAILILGLVIYTGFTIPTRNMLGWSRWMNYIDPIAYGFESLMVNEFHNRQFLCPDSAFVPSSGAYDSQPLAYRVCSTVGSVSGSRYVQGDDYLNQSFQYYKSHQWRNLGIMFGFMFFFMFTYLTATEYISESKSKGEVLLFRRGHAQPTGSHDVEKSPEVSSAAKTDEASSKEATGAIQRQEAIFQWKDVCYDIKIKGEPRRILDHVDGWVKPGTCTALMGVSGAGKTTLLDVLATRVTMGVVSGEMLVDGRPRDQSFQRKTGYVQQQDLHLHTTTVREALRFSALLRQPAHVPRQEKIDYVEEVIKLLGMESYADAVVGVPGEGLNVEQRKRLTIGVELAAKPQLLLFLDEPTSGLDSQTSWSILDLIDTLTKHGQAILCTIHQPSAMLFQRFDRLLFLAKGGKTVYFGEIGEKSSTLASYFERNGAPKLPPDANPAEWMLEVIGAAPGSHSDIDWPAVWRDSPERRAVHEHLDELKRTLSQKPIDPSKADPGSYDEFAAPFTIQLWECLLRVFSQYWRTPVYIYSKTALCVLTALYIGFSFFNAQNSAQGLQNQMFSIFMLMTIFGNLVQQIMPNFCTQRSLYEVRERPSKTYSWKAFMAANIIVELPWNTLMAFLIFVCWYYPIGLYRNAEPTDSVHERGALMFLLIWSFLLFTSTFAHMMIAGIELAETGGNLANLLFSLCLIFCGVLAPPQSLPGFWIFMYRVSPFTYLVSAMLSTGVSGTNAVCEPVEFLHFDPPSNMTCKDYMADYISTRGGYLENPSATSDCTFCTISSTDTFLSAVSSHYSDAWRNFGIMWAYIIFNIFAAVFIYWLARVPKGKRTKGST

>AfuabcF

MALNSTDNRWSTGEDTPSEAQLPDGEERLDAAPDEKVTAEDIDRRLTNLVRKISAQSRRSHHSFLFGAGENSSLNPQSPSFDARKWARAFYNARYRQDDGHPPRVVGVAFKNLNVFGYGSPVDYQMSVGNALLKVPTMVRQALGGGKQRVDILHDVEGLVLPGEQLCVLGPPGSGCSTFLRTIAGETHGLNVDAASYINYHGISPKQMSTAFRGEAIYTAEVDAHFPMLSVGDTLYFAALARAPQVIPGGLSRQEYAKHLRDVIMAMFGIGHTINTRVGNDFVRGVSGGERKRVTIAEAALGYSPLQCWDNSTRGLDSANAVEFCRTLRTQSDVFGITSCVAIYQAPQAAYDLFDKVLVLYEGWQIYFGAAHEAQAYFEQLGFQCPESQTTADFLTSMCSPAERIVKPGFEHMAPRTPEEFAQRWKESPQRQSLLHAIEKYSTEHPLDGPDLHQFALSRRAEKSHRQREKSPYTLSYRGQVKLCLWREWQRLKNDPSVTLAMLIGNFFEALIIASIFYNLTGDTSSFYYRGALLFMMVLLNAFASVLEILTLYEKRTIVEKQSRYAYYHPSAEALSSFIMSLPYKFVNSSLVNLTLYFMSNLRREPGPFFFFLLISTSMMLAMSMFFRWFASLTKTIDQALAPSSIILLALVLYTGFTIPVSYMRGWASWIRWLNPVSYGFEAVMINEFHGREFPCSSFVPSGPGYEDVSRTQRVCSTVGATSGSDVVSGDVFVRSSYGYVNSHRWRNFGIIIAMTVFLAVCHFVTTELVASKRSKGEVLVFRRGSAHIARAKQGQRDEEQPSASAVPSEKYSEAPTPVEGVETQTSIFHWEDVCYDVKIKNETRRILDHVDGWIKPGTLTALMGVSGAGKTTLLDVLASRTTVGVVTGETLVDGRQRDSSFQRKTGYVQQQDLHLATTTVREALEFSALLRQPPQYSREEKLEYVEKVIDLLHMRDYADAIVGVPGEGLNVERRKRLTIGVELAARPKLLLFLDEPTSGLDSQTSWSICNLMETLTRNGQAILCTIHQPSAMLFQRFDRLLLLAKGGKTVYFGEIGSGARTLMDYFVRNGGPPCPKGANPAEHMLEVIGAAPGAHTDIDWPAVWRNSPEYQQVRQELSRLRQLASQPSSVHSDDPSSYSEFAAPFPAQLGQVGRRVFQQYWRTPSYLYSKAILTVGSSIFIGFSFFKGDNTAQGLQNQVFGVFVFLFVVIQLIFQIIPTFVTQRTLYESRERQSKTYSWQAFVLSNIAVEFAWNTIAAVLCFLAWFYPVGLYRNAEYTDSVHSRSTLVFLIIWATFLFASSFAHLLIAGVESAELASALANIMGIMMYAFCGILAGPHALPGFWIFMYRVNPFTYLVSGLLSASLGDAPMHCAANEFLAFSPPANRTCGEYMEDYMALAGGYLLDSAARGDEQCQYCRVDNTSQYLRNFSIDFATRWRDFGLLWVYVAVNTFGAVFLYWLCRVPKGKKRL

>FkABC1

MPASPNPSPAPGEAPMGAPGSIHGSHETVVDMEGIASTAHFTEKPKNASPNEKSSSSSSDDDEDEEGMNEMERRHSIVRDLARQYTQTSMQNFQGDGAALFAADDKDSPLNPQSEKFNARAWAKAVAKTMSEHGSGFRQSGLCFQDMNVFGYGAETDYQKDVGNVWLGIPSAITNLTSPNRGKRRIDILRGFDGVVKAGEMVVVLGPPGSGCSTFLKTISGETNGIYVDDKTYFNYQGVPADEMHNHHSGEAIYTAEVDVHFPMLSVGDTLTFASRARCPQNLPPGVAHNQYSDHFRDVVMAMYGISHTINTRVGDNYIRGVSGGERKRVTIAEATLSNAPFQCWDNSTRGLDSANAIEFCKTLRLQSELFGQTCAVSIYQAPQSAYDLFDKALVLYEGRQIFFGRAEDAKAYFINLGFECPDRQTTPDFLTSMTAPSERIVRPGWENRAPRTPDEFAARWKESREYQLLQNEIDTYKSLYPLNGSSADAFRENKQQTQAKGQRLKSPFTLSYVQQIQLCLWRGFKRLKGSPEVTIFSLIANTSTALIASSLLYNLPETTGSFQNRGIVLFIGVLANAFASALEILTQYAQRPIVEKHTRYAFYHASAEAFSSILVDMPYKILNSIFYNLTLYFMTNLNRTPGAFFFFLFVSFLMVLAMSGIFRTIASVSRTLSQAMVPASILILALVIFAGFVIPVDYMLGWCRWINYLDPVAYAYEALMINEFHNRNFTCNQFVPSAAIPTYADITGTMRACSAVGALPGQDYVNGDAFMNSKYKYYHSHKWRNVGIIIAFVLFFHLTYIAATELIAAKKSKGEVLVFRRGNMPAVSQGKGDAEAAYSGPIKAADKVQNENDNANIQGSTSVFHWNNVCYDIKIKGEPRRILDHVDGWVKPGTLTALMGVSGAGKTTLLDCLADRISMGVITGEMLVDGKIRDSSFQRKTGYVQQQDLHLETSTVREALTFSALLRQPATTPRAEKIAYVDEVIKLLDMEEYADAVVGILGEGLNVEQRKRLTIGVELAAKPPLLLFVDEPTSGLDSQTSWAILDLLEKLSKAGQSILCTIHQPSAMLFQRFDRLLFLAKGGRTIYFGDIGENSETLTSYFEKNGSSACPKGENPAEWMLEVIGAAPGSHSDIDWHQTWKDSPEYQAVQTELQRLKAEGPANSAAHDNDHGAYNEFAAPFWEQLKIATQRVFEQYWRTPSYIYSKAALCISVALFIGLVFLNAPLTMQGLQNQMFAVFNILTIFGQLVQQQMPHFVTQRSLYEVRERPSKAYSWKVFMLSQIIAEIPWNTLMSVLMFVCVYYPVGFDKNAAAAGQTAERGALMWLLFWQFLIFTCTFAHACIAITDTAEAGGNLANVLFMLCLFFCGVLASPDQMPGFWIFMYRVSPFTYLVSAIMATGLANTEVTCAANEYVIFDAPKGQDCATYLADYISAAGGYVLNESATSDCQYCPMKDTNVFLKALSSSYDNRWRDWGIGMVYIVVNIAASLALYWLVRMPKGKKQKQS

>AfuatrI

MRRSNVVPVHSLTSSTNTGRDSRGEKYDELTPVATRRASISPDEARYLTQLASRDNAVSRVSTVADISLDDPALNPENKDFDLYKWLRKVVHVLNEEGVPRKEASIFFQHLRVSGTGAALQLQKTVADIITAPFRRETWNFRNKTSKTILHDFNGMLHSGELLIVLGRPGSGCSTFLKTLSGELHGLNVDEKTVLHYSGIPQSTMIKEFKGEVVYNQEVDKHFPHLTVGQTLEFAAAVRTPSKRLGGMSRNEYAQMMTKVVMAVFGLSHTYNTKVGNDTVRGVPGGERKRVSIAEMALAGAPLAAWDNSTRGLDSATALKFVESLRLAADLNSSAHAVAIYQASQAIYDLFDKAVVLYEGRQIYFGPASKAKAFFERQGWFCPPRQTTGDFLTSVTNPIERQARPGMESQVPRTAAEFEAYWLESEEYKELQREMAAFQGETSSQGNEKLLEFQQRKRLAQASHTRPKSPYLLSIPMQIKLNTKRAYQRVWNERTSTMTTFIGNTILALIVGSVFYGTPTATAGFYAKGATLFYAVLLNALTAMTEINSLYSQRPIVEKHASFAFYHPATEAIAGVVSDIPVKFLMAIAFNIILYFLSGLRREPSQFFIYFLITFIIMFVMSAVFRTMAAITRTVSQAMTLAGVLILMLVIYTGFVVPVNYMHPWFKWIHYLNPIFYAFEILIANEFHGREFTCSQFIPVYPNLPGDSFVCSSRGAVAGRRTVSGDAYIEASYSYSYSHVWRNFGILIAFLIGFMVIYFVATELNSATTSSAEVLVFRRGHEPAHLKNGHEPGADEEAGAGKTVVSSSAEENKQDQGITSIPPQQDIFTWRDVVYDIEIKGEPRRLLDHVSGWVKPGTLTALMGVSGAGKTTLLDVLAHRTTMGVITGDMFVNGKPLDSSFQRKTGYVQQQDLHLETATVRESLRFSAMLRQPASVSKEEKYAYVEEVIKMLNMEDFAEAVVGVPGEGLNVEQRKLLTIGVELAAKPKLLLFLDEPTSGLDSQSSWAICNFLRKLADAGQAILCTIHQPSAILFEQFDQLLFLARGGKTVYFGPIGENSQTLLKYFESHGPRRCGDQENPAEYMLEVVNAGTNPRGENWFDLWKASKEAAGVQAEIDRIHESKRGEAESKDSTNPKDREHEEFAMPFFKQLPIVTVRVFQQYWRLPMYIAAKMMLGICAGLFIGFSFFKADTSLQGMQNVIFSVFMLCAIFSSLVQQIIPLFITQRALYEVRERPSKTYSWKAFMIANIIVEIPYQILMGILVFGCYYYAVNGVQSSDRQGLVLLFCIQFFIYASTFADFVIAALPDAETAGAIVTLQFSMALTFNGVMQTPEALPGFWIFMYRVSPFTYWVGGMAATQLHGRAVKCSAAETAIFNPPSGLTCQEYMADYMAVAPGHLSNPNATSSCEFCSLSVADQYLASVNIYWSERWRNFGIFWAYVVFDIAVAVMLYYCFRVKKWNFSFGKRKKSKAA

>PechB6HED3

MTSETSSFELREKDEQSPPHTGDASTLGDSPMELIDQVDVETLTKIATARSRRQSTLGTTDNLAVLAQQDPALDPQSGKFDLRKWLKAAFNDINREGHSGHTSDVVFKQLNVYGSGAALQFQDTVTSTLTAPFRLPQIIRESKSPQRRILKDFNGLLKSGELLLVLGRPGAGCSTLLKSMTGELHGLNLDKDSVIHYNGIPQSRMIKEFKGELVYNQEVDRHFPHLTVGQTLEFAAATRTPSHRFQGMSRAEFAKYVAQITMAVFGLSHTYNTRVGDDFIRGVSGGERKRVSIAEMAVAHAPIAAWDNSTRGLDSATALKFVEALRLSSDITGSCHAVAAYQASQSIYDIFDKVIVLYEGHQIFFGPAAAAKSYFERQGWACPARQTTGDFLTSITNPQERQTKPGMENRVPRTPEDFETAWLKSPEYKQLLNETAEYEGKNPIGKDVQALADFQQWKRGVQAKHTRPKSPYIISVPMQIKLNTIRAYQRLWNDAASTISVVVTNIIMALIIGSVFYGTPDATAGFTSKGATLFFAVLLNALTAMSEINSLYSQRPIVEKHASFAFYHPATEAIAGVISDIPVKFALSVVFNIILYFLAGLKREASNFFLYFLITFIITFVMSAIFRTLAAVTKTISQAMGLAGVMILVLVVYTGFVLPVPSMHPWFEWIHYLNPIYYAFEILIANEFHGREFPCSSYVPSYADLSGHAFSCTAAGSEAGSRTVSGDRYIQLNYDYSYSHVWRNFGILIAFLIGFMIIYFVASELNSATTSTAEALVFRRGHEPASFRQDHKSGSDVESTKLSQAQPAAGTEDKGMGAIQPQTDTFTWRDVSYDIEIKGEPRRLLDNVSGWVKPGTLTALMGVSGAGKTTLLDVLAHRTSMGVITGDMFVNGHGLDQSFQRKTGYVQQQDLHLDTATVRESLRFSAMLRQPASVSVKEKYDYVEDVIKMLKMEEFAEAIVGVPGEGLNVEQRKLLTIGVELAAKPKLLLFLDEPTRQSSWAICSFLRKLAEHGQAVLCTIHQPSAMLFQQFDQLLFLARGGKTVYFGPVGENSSTMLEYFESNGARKCADDENPAEYMLGIVNAGQNNKGQDWYDVWKQSDESKQVQTEIDRIHKEKEHQPPSADDSAQSHSEFAMPFMFQLSQVTYRVFQQYWRMPSYILAKWGLGIVSGLFIGFSFYSAKTSLQGMQTVIYSLFMICTIFSSLAQQIMPVFVSQRSLYEGRERPSKSYSWKAFLIANIIVEIPFMVVMGVLTYASYFYAVVGVPSSLTQGTVLLFCIIFFIYASTFTHMVIAGLPDEQTASAVVVLLFAMSLTFCGVMQPPSALPGFWIFMYRVSPFTYWVGGMASTQLHNRQVVCSAAELAVFDPPSGQTCGQYLMQYAAAAGGKLLNPEATSDCSYCSLEVADQYLSTANIFYSERWRNFGIMWAFIGFNIFVATIMYYLVRVKRWSSADLKESVMKLIPGKKSKGGN

>BociBMR3

MERLEHMSWRNKTPCMGLSWGTQHWTPTPQILTYINGLKCKFWHVQVFIWTQTNCTCRMMRLVDENGVIQRRAGIVFKNLKVCGSGSAINVQKNVGSLLMAPLRFKEFIGKGPEKTILNDFNGVLKSGEMLIVLGRPGSGCSTFLKSLMGELYGLDMKAQSEIHYNGITQKQMLKQFRGEIVYNQEVDKHFPHLTVGETLEFAASVRTPQQRLVEGTTRSAWAKHMTKVVMAIYGLSHTYNTKVGNDFVRGVSGGERKRVSIAEMALAGSPIASWDNATRGLDAATALEFTKSLRMTANLSGSCHLVAIYQASQQIYDQFDKAIVLYEGRQIYYGPCDQAKQYFEDMGWECPSRQTTGDFLTSITNPSERKARPGYENKVPRTPEEFEKYFKDSKIFQRMMSEMKSHEEEFPMGRKTLEQFKASRKGMQADHLRPESPYTVSIVMQTKLCARRAVQRLWNDKTSTITTIVGQIAMALIIGSIFYNTPSNTASFFQKGGVLFFAVLLNALIAISEINTLYSQRPIVEKQASYAFYHPFTEALAGVVVDIPVKFAIATCFNIILYFLSGLKREAGAFFVFFLFNFVAILTMSQIYRSIAAATKTISQALAIAGVATLAIVIYTGFVIPRPLMHPWFKWISWINPVAYAFEALFVNELHGKEFVCSTLVPTGPGYVQAGNNFVCAVAGSVVGATTVSGDDYLQAQFQYSYSHIWRNLGFLFAFMIFFLAFYLLATEFNASTDSKAEVLVFRRGHVPTNLLAAEKAAKNDEEAHAGNGSAVKEGNSDKQGDEVQALAPQTDIFTWKDVCYDIKIKNEPRRLLDNVSGWVKPGTLTALMGVSGAGKTTLLDVLAQRVSMGVITGDMLVSGKPLDASFQRKTGYVQQQDLHLETTTVREALRFSAMLRQPKTVSKKEKYDFVEEVIKMLNMEEFSEAVVGVPGEGLNVEQRKLLTIGVELAAKPALLLFLDEPTSGLDSQSSWAIVSFLRKLADNGQAVLATIHQPSAILFQEFDRLLFLAKGGRTVYFGDIGHNSETLLNYFESHGAAKCGEDENPAEYMLTMVGAGAQGKSTQDWHEVWKASDEAKGIQTEISRIQQEMGHQPSQDDSNSHGEFAMPFTVQLLEVMKRVFQQYWRTPGYVYSKLVLGVASALFIGFSFFHADASQQGLQDVIFSIFMITTIFTTLVQQIMPRFILQRDLYEVRERPSKAYSWKAFIIANIAVEIPYQIILGIMVFASYFYPIYTKNGIPPSGRQGLILLLLIQFFVFASTFAHMLISALPDAETAGNIATLMFSLTLTFNGVFQPPQALPGFWIFMYRVSPLTYLVSAIASTGLSGRQVICSDNELAVMQPPAGDTCGSYLQSYATAAGGSIYNPEAMADCQYCSSSNADQFLSSVAISYTTRWRDYGIVFVYIFFNIFMAVLLYYLIRVRKSSGKSLKEKFGALGALFKKN

>ScSNQ2

MSNIKSTQDSSHNAVARSSSASFAASEESFTGITHDKDEQSDTPADKLTKMLTGPARDTASQISATVSEMAPDVVSKVESFADALSRHTTRSGAFNMDSDSDDGFDAHAIFESFVRDADEQGIHIRKAGVTIEDVSAKGVDASALEGATFGNILCLPLTIFKGIKAKRHQKMRQIISNVNALAEAGEMILVLGRPGAGCSSFLKVTAGEIDQFAGGVSGEVAYDGIPQEEMMKRYKADVIYNGELDVHFPYLTVKQTLDFAIACKTPALRVNNVSKKEYIASRRDLYATIFGLRHTYNTKVGNDFVRGVSGGERKRVSIAEALAAKGSIYCWDNATRGLDASTALEYAKAIRIMTNLLKSTAFVTIYQASENIYETFDKVTVLYSGKQIYFGLIHEAKPYFAKMGYLCPPRQATAEFLTALTDPNGFHLIKPGYENKVPRTAEEFETYWLNSPEFAQMKKDIAAYKEKVNTEKTKEVYDESMAQEKSKYTRKKSYYTVSYWEQVKLCTQRGFQRIYGNKSYTVINVCSAIIQSFITGSLFYNTPSSTSGAFSRGGVLYFALLYYSLMGLANISFEHRPILQKHKGYSLYHPSAEAIGSTLASFPFRMIGLTCFFIILFFLSGLHRTAGSFFTIYLFLTMCSEAINGLFEMVSSVCDTLSQANSISGILMMSISMYSTYMIQLPSMHPWFKWISYVLPIRYAFESMLNAEFHGRHMDCANTLVPSGGDYDNLSDDYKVCAFVGSKPGQSYVLGDDYLKNQFQYVYKHTWRNFGILWCFLLGYVVLKVIFTEYKRPVKGGGDALIFKKGSKRFIAHADEESPDNVNDIDAKEQFSSESSGANDEVFDDLEAKGVFIWKDVCFTIPYEGGKRMLLDNVSGYCIPGTMTALMGESGAGKTTLLNTLAQRNVGIITGDMLVNGRPIDASFERRTGYVQQQDIHIAELTVRESLQFSARMRRPQHLPDSEKMDYVEKIIRVLGMEEYAEALVGEVGCGLNVEQRKKLSIGVELVAKPDLLLFLDEPTSGLDSQSSWAIIQLLRKLSKAGQSILCTIHQPSATLFEEFDRLLLLRKGGQTVYFGDIGKNSATILNYFERNGARKCDSSENPAEYILEAIGAGATASVKEDWHEKWLNSVEFEQTKEKVQDLINDLSKQETKSEVGDKPSKYATSYAYQFRYVLIRTSTSFWRSLNYIMSKMMLMLVGGLYIGFTFFNVGKSYVGLQNAMFAAFISIILSAPAMNQIQGRAIASRELFEVRESQSNMFHWSLVLITQYLSELPYHLFFSTIFFVSSYFPLRIFFEASRSAVYFLNYCIMFQLYYVGLGLMILYMSPNLPSANVILGLCLSFMLSFCGVTQPVSLMPGFWTFMWKASPYTYFVQNLVGIMLHKKPVVCKKKELNYFNPPNGSTCGEYMKPFLEKATGYIENPDATSDCAYCIYEVGDNYLTHISSKYSYLWRNFGIFWIYIFFNIIAMVCVYYLFHVRQSSFLSPVSILNKIKNIRKKKQ

>CaSNQ2

MSSEDIGSSSSSLQEYVGQQQHNKIQPSTDDDYNEDDYESRRLHLVRTVSSINHHNFDEKFDTISREISRQVTNKEGEFQLRLDEFNLAKILANFVYFAKKQGIVLRKSGITFQDLCVYGVDESFAIAPTVTDLLKGPVGAVQAILSQMKTPPRKILKNLNGFAKPGESVLVLGRPGAGCTTFLKALSGTDFDLYKGVTGDIRYDGLPQKEMLKLFKNDLVYNPELDVHFPHLTVDQTLTFAIACKTPEMRINGVTRDEFINAKKEILATVFGLRHTYHTKVGNDFVRGVSGGERKRVSIAEALACNGSIYCWDNATRGLDASTALEFAQAIRTSTKLLKTTAFVTIYQAGEGIYETFDRVTVLYDGHQVYYGPANKAKKYFEDMGWECPPRQSTAEFLTAITDPIGRFPRAGWENKVPRTAQDFEHYWLNSPQYQELMQEIKDYNDEIDEDETRSKYYQSIQQEKMKGSRTKSPFTISYLEQLKLCFIRSYQRILGDSAYTITLMFASVAQAFVAGSLYYNTPDDVSGAFSRGGVIFFAVLFMSLMGLAEISASFSSRPILMKQKNYTMYHPSADSLSNFVMSIPISIFINTFFVIILYFLSNLARDAGKFFICYLFVIMLHLTMKSMFQAIAAINKSIAGANAMGGILMLASLMYSSYMIQRPSMHPWFKWISYINPVLYAFEAVIASEFHGRKMQCTSQYLTPSGPGYENLGAGEQVCTFIGSVPGQSWVLGDDYLRIAYTYRFSHVWRNLGILFGFLAFFLAIATLGTEYVKPITGGGDKLLFLKGKVPEHITLPSEKKEEDIESGGNSDTTATSNGTLSQGKSEEKAAIADDGLKAKGVFVWKDVDYVIPYEGKKRQLLQNVSGYCVPGTLTALMGESGAGKTTLLNVLAQRVDFGVITGDMLVNGRPLDTSFSRRTGYVQQQDIHFSEVTVRESLQFAARLRRSNDVSDAEKLEYVEKIIDVLDMRGYADAVVGRLGNGLNVEQRKKLSIGVELVAKPSLLLFLDEPTSGLDSQSAWAIVKLLRDLANAGQSILCTIHQPSATLFEEFDRLLLLKKGGIVTYFGDIGPRSRTILDYFERNGARHCDDKENPAEYILEAIGAGATASTDFDWGEIWAQSPEKVQTDAKRDELINESAKNATDTSATDSPSEKNLTSKYATPYWYQFRHVTHRTSLIFYRDPDYIAAKVFLMTIAGLFIGFTFFGLKHTKTGAQNGMFCAFLSCVIAAPLINQMLEKAGSRDIYEVREKLSNTYHWSLLILPQIIFEVIYMIIGGTIMFVCLYFPTQVSTVASHSGMFYFSQAIFLQTFAVSFGLMVSYVSPDIESASVIVSFLYTFIVSFSGVVQPVNLMPGFWTFMNKVSPYTYFIQNLVSSFLHDRTIRCNAKELSYFNPPSGQTCKEFASAFISRNGGYLVDEGATSNCGYCNFSNADQYLLTIGAKFSYRWRNIGFFCVYIIFNISVCLVLYYFLRYRKVSFNVTGLVNKFKKSKK

>PipPDR12

MSSTSSSINDKDKESSNIDSPKDTVPYEPTRFVGDLENQNEDDIYSEQLSRILTQSEAVQKIQSLARTMSRMTKKELAAFEVNQDDFDLKILLHYLRAKSEEQGIESCSAGVAFKNLTATGIDVSAAYGPTVDEMLRNFFMWPIRFAKREHVKTRQIIRNFTGSIEAGELCLVLGRPGAGCSTLLKCCTGNTSELLSVEGEFSYDGLDQAEMMKDYKGYVIYNPELDTHFPHITVKQTIDFALKMKTPAKRVDGIPRKKYIDTMRDLWCTVFGLRHTYGTKVGNDFIRGVSGGERKRVSIVEALATGASVYAWDNATRGLDASTALEFTQAIRTSTNLLNASGMVAIYQAGENIYELFDKVCVLYNGKQVYFGPAEKARKYFEDMGWYKPPRMTTPEFLTAVTDPSGRFIREGFKNKVPENSEDFEQYWLNSPEYQECLRSHDQYIQDHNPEETRQRLATAKSQTRQKAVRSKSRFVASYPNQIAYCVTRGFQRTKGEIAYTLVYLSSFLTKGFIVGSMYWNIPKDTSGLFSRSGILFYCLLFCAVTSLSEISHTYTNRPIILKQKSYSLYHQSAESLQEIITELPTKLVAVIILALTTYFMPGLRLSDGGSAFWMYLLFLLLIQQCMSFMFKLIATLTRDAGTAHACGGLLALMMCVYTGFIIPLPYMHHWIKWFNWINPMRYCYESLLATELHSREMKCSEYIPNGPDYEGISMENSACTTTAYNHTTGLVSGNAFLTATYNYRYSHVWRNFGINIAWTAGFIIINTILSEFVKNVEGGGDMLLYKRGHMPKEGIEAVDGKVASKQEMMEALNGPDVDLKKVIAERDVFTWQHLDYVIPYGGATRQLLNDVQGYVKPGTMTALMGESGAGKTTLLNTLSQRINFGTITGDIFVNGRPLDSSFKRRTGFVQQSDLHLAEYSVRESLRFAVNLRQSEKVPQAEKYEYVEKIINLLGMQNYAEAIIGKIGRGLNVEQRKKLSIAVELVAKPSLLLFLDEPTSGLDSQSAWSIIQFLRALSDSGQAILCTIHQPSATLFEVFDRLLLLKKGGRTVYFGDIGPNSSTMLSYFERESGIKCGVSENPAEYILNCIGAGATAHASADWGDLWVSSPEHAAVTEEISRLNTELQKRPLPENIEDLQSKFATSYPHQIKILFLRTMVQFWRSPVYIRAKFLEAVVCAIFVGFSFVKVGHGLQEAQFGLTSIFMMLIISLAMINQMHVFAFDSRELFEARESASNTFHWSTLLLAQTWWETIWCMACQFLCFVCYYFPAGFSGTAHHAGYFFLQFVIIFPIYYCSYGLWVLYFSPDVPSAGMINSNFFAAMLLFCGVLQPPQFMPGFWTFMYKLSPYTYFVQSFVAPLVHNRKLVCRTNEYTLITPPEGQTCSEFLDPFIESDGGYLGNPDATESCEYCPYTYQSQVMEQFNIKWSYRWRNFGFFFAYIIFNYVALLSCYYLMRVKVWNMKSILNFKKWFNGPRKERHDPETNIFAAQPADAKLAVLKKKE

>ScPDR12

MSSTDEHIEKDISSRSNHDDDYANSVQSYAASEGQVDNEDLAATSQLSRHLSNILSNEEGIERLESMARVISHKTKKEMDSFEINDLDFDLRSLLHYLRSRQLEQGIEPGDSGIAFKNLTAVGVDASAAYGPSVEEMFRNIASIPAHLISKFTKKSDVPLRNIIQNCTGVVESGEMLFVVGRPGAGCSTFLKCLSGETSELVDVQGEFSYDGLDQSEMMSKYKGYVIYCPELDFHFPKITVKETIDFALKCKTPRVRIDKMTRKQYVDNIRDMWCTVFGLRHTYATKVGNDFVRGVSGGERKRVSLVEAQAMNASIYSWDNATRGLDASTALEFAQAIRTATNMVNNSAIVAIYQAGENIYELFDKTTVLYNGRQIYFGPADKAVGYFQRMGWVKPNRMTSAEFLTSVTVDFENRTLDIKPGYEDKVPKSSSEFEEYWLNSEDYQELLRTYDDYQSRHPVNETRDRLDVAKKQRLQQGQRENSQYVVNYWTQVYYCMIRGFQRVKGDSTYTKVYLSSFLIKALIIGSMFHKIDDKSQSTTAGAYSRGGMLFYVLLFASVTSLAEIGNSFSSRPVIVKHKSYSMYHLSAESLQEIITEFPTKFVAIVILCLITYWIPFMKYEAGAFFQYILYLLTVQQCTSFIFKFVATMSKSGVDAHAVGGLWVLMLCVYAGFVLPIGEMHHWIRWLHFINPLTYAFESLVSTEFHHREMLCSALVPSGPGYEGISIANQVCDAAGAVKGNLYVSGDSYILHQYHFAYKHAWRNWGVNIVWTFGYIVFNVILSEYLKPVEGGGDLLLYKRGHMPELGTENADARTASREEMMEALNGPNVDLEKVIAEKDVFTWNHLDYTIPYDGATRKLLSDVFGYVKPGKMTALMGESGAGKTTLLNVLAQRINMGVITGDMLVNAKPLPASFNRSCGYVAQADNHMAELSVRESLRFAAELRQQSSVPLEEKYEYVEKIITLLGMQNYAEALVGKTGRGLNVEQRKKLSIGVELVAKPSLLLFLDEPTSGLDSQSAWSIVQFMRALADSGQSILCTIHQPSATLFEQFDRLLLLKKGGKMVYFGDIGPNSETLLKYFERQSGMKCGVSENPAEYILNCIGAGATASVNSDWHDLWLASPECAAARAEVEELHRTLPGRAVNDDPELATRFAASYMTQIKCVLRRTALQFWRSPVYIRAKFFECVACALFVGLSYVGVNHSVGGAIEAFSSIFMLLLIALAMINQLHVFAYDSRELYEVREAASNTFHWSVLLLCHAAVENFWSTLCQFMCFICYYWPAQFSGRASHAGFFFFFYVLIFPLYFVTYGLWILYMSPDVPSASMINSNLFAAMLLFCGILQPREKMPAFWRRLMYNVSPFTYVVQALVTPLVHNKKVVCNPHEYNIMDPPSGKTCGEFLSTYMDNNTGYLVNPTATENCQYCPYTVQDQVVAKYNVKWDHRWRNFGFMWAYICFNIAAMLICYYVVRVKVWSLKSVLNFKKWFNGPRKERHEKDTNIFQTVPGDENKITKK

>UsmaQ4P757

MEANTSAAHPAPPIANTSVNEYSDAVDVGGTAAATTFAGSPVINSDGQHSSTDHNHASSAPEMPPVERIKSGRGSVPLSSIDPQGMAELTRRLTEHSMRARSRTTDGDGGEEALGFDPFDKNGKFDLERFLRLVMQQAEGAGNEVREMGLVWQNLTVTGLGSGYALGDTVGSLPLKPFEALKNIKSLLHPPVKVIIDNFEGCIKPGEMLLVLGRPGAGCTSFLKTLASYRDGFQDITGTLLYQGMDHTVIDKRLRGDVVYCPEDDIHFPSLTVWQTLAFAVATRAPQARRRLNLLQSEDTQTRDGYIKTLVEVVATILGLRHTYNTKVGNDFVRGVSGGERKRVSVAETFASRAKVALFDNSSRGLDSSTALEFVKSLRVSTDIANTTTAASIYQAGEGLTQLFDKVLVINEGRQVYFGPTSEAPDYFKEMGYIPQERQTTADYLVACTDAHGRRLREGYEKRAPRTAEEMAKYWQASPQGHKNRQEVEAYLEELTSKVDDAAVKRYKEVAREEKAKNTRKGSAYIISLPMQIRLAVKRRAQITWGDIATQVIIACASMFQAIIMGSVFLLMPKNTSGFFSRGGVLFFALLYNSFTAMSEITAGYAQRPIVIRHRRFAMIHPFSDALANTLLDMPIRLMTLTLFDVILYFMVGLQYTAGQFFVFYSTTALITFTMVAFFRMLSAATKSESLATMLGGLAIIDFALYTGYVIPRPSMVVWWKWLSYCNPVAFAFEILLTNEFRTLNVPCANFIPAGQAYADVSDQYKTCAVASAQPGQDIVIGSEYLAQSYGYTWSNAGRNAGIIFGFWFFFLIVYSLASEFQKDPSASGGVMVFKRGAAPKEVVQAAKASGDVEAGDAAGHTERVDREQDEQADKAVGKLESSTSVFAWKNVNYDVLIKGTPRRLLNDVSGFVAPGKMTALMGESGAGKTTLLNVLAQRTDTGVVRGLFSVNGAPLPKSFQSNTGYCQQQDVHLGTQTVREALQFSALLRQPRETPKEEKLAYVENVISMLEMESWAEALVGEVGMGLNVEQRKRLTIGVELAAKPKLLLFLDEPTSGLDAMAAWSVVRFLRKLADAGQAILCTIHQPSGELFNQFDRLLLLQKGGKTVYFGDIGPNSTKLVEYFGERADKRCGENDNPAEYILDVIGAGATATTDKDWHELFRNSYLFTDMMKEVERIDSLGADHPATAEEEAMGMREYAEPFSVQMTQVMRRAFMHYWRDTTYIMSKLMLNIIAGLFIGSSFWGQGRTQTSASLQNKIFAIFMALVLSTSLSQQLQPVFIQFRALYEVRERPSKMYSWPVAVTAALVVEIPWNLLGGTLFWASWYFMVGFPYGKTAALVWGMYMLFQIYYQTFAAAVAAMSPNPMIASILFSTFFSFVIVFCGVVQPPPLLPYFWRSWMFVASPFTYLLESMLGAVLNNQPVRCSAQEYNRITPPPGQSCAAYLANFVTSLDGPNLGTGYYIDGPNGSCDYCQFRIGNDYLRSIELNASNRFRDIGIICIYIVFNVLLCFTLFYFFRVFKLSSFKKKDGEKQPTIADQDEKPTVAAETVSRADAGAEKAVHALTSTSAAAKGPGHEGASQPGLVANPFGGGAGQHGVQDVQAKLRDNT

>CociA8NBC6

MADQQPHHLTDIVEEPASTGVSRIGSQMTLQPKPSSVDDKLEKDIEDDDDRGRHPPPSSPKVPHRTRRTSSASRVSMDFFDPSGMQSLRRTLTHERVGLPKGQSEPSESSDTSSDHTLIVDGDNFDLEKTIKTIIRRRDQSEIKPRQLGVVFRNLRVVGLKAAASFQPTVGSLFNPADVVQNIQNARHPPVRNILDGFEGVVRPGEMLLVLGSPGAGCSTFLKTLANHREEYHSVEGEVHYDSITPEELKKWFRGDVQYSPEDDVHFPTLTVEQTIKFAARTRTPRNRIEYTRAQFIDTLSNILTTVFGLRHARKTPVGDAMIRGVSGGEKKRVSIAESLATRACIACWDNSTRGLDASTALEFVRALRIGTDTMRLSTIVSIYQAGESLFSHFDKVCVIYEGQMAYYGPADQAKDYFYEMGYVPANRQTTPDFLVAVTDPNARIAREGVTNQPRTAEEFAAYFKASAHGQRNKAEIEEYIAEHVGKPDAAQRYIDSARAEFAKRSGKKNPYMLTIPQQVAAVMRRKVQILRGDMLATGLNLFSYVFQALIMGSIFLKMPEQTSAYFSRGGVLFFALLFSALATMAEIPALYAQRRIVLRHEKAALYHPFVEALAHTLVDIPITFCILSLFCIILYFMTGLQRSVDQFFVFFLFVFAMAVTMKSWFRGIAAAFKSEATAQAVAGISVLALSIYTGYTIPKPTMIGALRWITYINPLRYGFEAILTNEFRTLNGLCTSLVPSGPGYENVSLANQVCAVVGALPGEAFVNGARFAELSYSFKWSNTWMNLGIVIAFAIGFLIVLLIFAEFNTTSSADTATTLFKRGSKKAVAAASSGSDEEKKGSPSGTVVVDEKGKDTSALKDEPALRMTDTFTWQHVHYTVPIPGEADRTLLSDVSGYVAPGKLTALMGESGAGKTTLLNVLAKRVYSGVVTGDMFVNGQSLPADFQSQTGYCQQMDTHMPNATVREALLFSAKLRQPPSVPLEEKEAYVEKCLKMCGLEEYADAIVGTLNVEYRKRTTIAVELAAKPKLLLFLDEPTSGLDSQSAWAIVSFLRSLADQGQAILCTIHQPSAELFQVFDRMLLLKKGGQTVYFGDLGHNATTLIQYFERNGARHCDPSENPAEYMLDVIGAGATATTEFDWHGIWKSSPEATAVQEELEAIHTEGRNRPAVEAELHTEFATSWLFQVKELFIRNVQSYWRDPTYIMAKMVLNVSSGLFIGFTFFKAKDTIQGTQNKLFAIFMVTILSVPLASQLQVPFIKLRTIYEIRERPSRMYSWTALITSQILVELPWNIIGSTLLFMCWYWPVGFLASRGGYTYLMLGIIFPLYYTTIGQAVAAMAPSVEIAALLFSLLFSFVIAFNGVLQPFRELGWWKWMYRLSPYTYLIEGLLGQAIGRQDVECAPYEYVQITPPEGLSCGGYMDPFISFAGGYLTNPNATDACNYCSVKTTDQFLLASFNISYSHRWRNVGLMLAFTVFNNTYGEFAA

>LabiB0DZC0

MGSYIHGEQLNDIHEEPEASTSYAHSSPSDLSPPAALRRSISERPATSSNLLSRLHPPHARQRSCSHVAVDFFDPMGVRRLSHSLAKTPSRAATEVEEAPSLDSEETVIVGEGFDFEKGLRQYLKKRDEAGVKSRRLGVMFQDLRVVGLGATASYQPTLGSLLNPINIIGAIRSARHPALRDIISGFHGVVRPGEMLLVLGRPGSGCSTFLKTLANQREEYHSVEGDVFYDALTPQQILKHYRGDVQYSPEDDIHFPTLTVDQTIHFAAKTRAPHPRIHDQTRSQFTRRITDVYCTIFGLNHVKDTPVGDSSIRGVSGGEKKRVSISETLATRSLITSWDNSTRGLDSSTALEFVRALRIATDLVGNSTIVSIYQAGEQLYEVFDKVCLIYEGRMVYYGPANQARQYFLDMGYVPKNRQTTADFLVSVTDPLGRHTRDEIKMEEGDIEGAKEKKKGVALDHESGSPARTLQPIPHTADEFEAYYMDSEVRKWNLEDMAAYKRDFVDSKEVAAAFEESAKEEHARHTRRQVKIVLLRRVQIMKGNWTAQALQTMTFVLQAVIIGTTFLKIPDTTAAYFSRGGVLFFAVFLPALFTMSEIPALFSQRRIIHRHQNAAMYHPMVEAIAMALVDVPFTFVTVVLFTIILYFVVRLQTSPGQYFTFFIFIFTVSMSMKAFFRGLAAMFRKEAPAQAVAGVLLLALSLYTGYQIPMPSMIGALRWISYINPVRYAFEGIMVNEFHTLDGVCSTLVPSGPGYETVSLANQVCTVLGSQQGQDRVNGNAYVNLSFEYSFSHLWRNYGILVAFGIFFLVCLVFFTEINTAVASESSVILFKRGSKAELVKDAEAAVTSGDDEEKEKPRRPDSQEVMEADEEKAKEAMIDQPKMTNVFSWQHLQYTVNVGGEQRVLLDGVSGYVAPRKLTALMGESGAGKTTLLNVLAERVSTGVIGGNRFFNGQALPIDFQAQTGYVQQMDTHLPTTTIREALVFSARLRQPPSVSVAEKDAYAEQCLKMCGLDSVADAMVGSLGVEQRKRTTIGVELAAKPQLLLFLDEPTSGLDSQSAWAIMAFLRSLADNGQAILCTIHQPSAELFQVFDRLLLLKKGGQTVYFGDVGKNATSVIEYFEAGGARECRPGENPAEFMLDVIGAGATAVSDRDWHDVWLQSKAFTIAEADIERLHEEGRKHPPVGATLKSEFAEPWAYQAKTLIQRSFTCYWRDPTYLMSKLTLNIIGGLFIGFTFFKAKDSIQGTQNKLFSIFMGTILSAPLGGQLHVPYIKMRNIYEIRERSSRIYHWSALVTAQILVELPWNILGSSLFFLCWFWTVGFETSRAGFTYFLFGVLFPVYYTTLALAVASMSPTAEIAGLLYSFVFSFVLIFDGVVQPFSQLNWWKWMYHVSPFTYLIEALVGQAFGHEQINCAEKELVTLQPVSGQSCGSYMARFISQKGGFVTNPDALSDCRFCTFRTTDQWLGPTFNIYYSRHWRDFGIFWAYIIINIFSVYLLTYLVRVRSYNGSSFFRKRIGVLLGKMKK

>AgoPDRE

MKNRSLRANGASIGGKNNISDIEEYNSNATIIRDLTFKVGAGEIVLVVGDKSSQFLRNLADGNSAISPENKLVFKNYEYLKFAERCTGEIIYSNAEDNHMKQLTVQETIDFAVNCNVDVAKEDKILLRDTLLSAFKLSEVRHTLVADENSSKLSVGERRRLSIIEAFLGNASLYLWDNCTNGLDSMTAMDIIQGLKLMARVTKTVNLFGCTQANGILLGQVDKVLVLIGDYQVFYGSYQDCILFFESLYFPKDPNLLDSEYFSAVIYGSIQSPLIKTDSDFHRQWIHSSYYRKVNDESQIKYSKKTIDLKDLKSLQPVSALRQVIYCVLRSWNMFRSDKFATGLQVTTAFLRSLTLGALFLDLPTTAVGSQARERLIFYILLMCMFSGITTVPLIFDRRPVIMKQIQMQFYRPWTDSLANTTLSLSVNLILVTISSSVLYTMVDFQRSLLRFSVFILIAITSDCIIHLMFLTIANISPNSALANGLSGILLISASVTGSLGIPLKQMNRWFKVLSLINPIRYAGETFLTNELFNVSLECADMLVPGGAAYQNVAGSFRTCVWPGAEPESSVINGTKYLSAQFAFSHAHFWKNYGILQAFSIFFFIASLLSAEYIMPLFCTKYAPRICWPREKYENCHPARIKLRKEVVIDNDSHSTFCSDRFIEKAYAVAPSSSCGTREFSYGNELYHSIISWSNLSYQVNGKMLLQNISGYIGPGLCLVLGESSSGKSALLNVLARREASGITGNLMLAGRPIKDYPNYHNEVGYVPQVDAHFGSLKVIEALEFSANIRGQRDKEHITEVLSMLNLSGQRYVDELCLQEKRILSIGAELASKPAVALFVDEPSMGMDSEAAIAMIERLNRLADEGQTILCSISQPSKSIFHYFDNLIVLDKNGQCVYFGSTEGAISYFTKHSAIQYDERKHNPSDLILSIIRSNLKDRTWAENWQCTKEYAAAEATRIELERKSFGAWASDQRHLDSFLTWPTCFCATLKRQFILIARDKSYLTSRIILSLTTGLYLAGSLWNTGNSGSALDTTIFSVFVFLAMSLPLAQQIQTKAATVMKAYITREMKSHTVGWSTLMAAQFLAEIPLLLSCCVLFYFCIYCTMGTSVNPAVMDAFFVNCLVFGLYCLSIGLATLFVGLKVYHSTALLSLVSSLLLAVCGILQPNLKVTPIWRTIYQMSPLTYFADTFTSLLLYDKHILCSQDDLISVSRPDGQSCSEYFGTFIQEFGGKIVDHADKNMCAYCSSDEISNFLSLRDMSYNHIWRNVAICSAFVALNFAAMICRFHLFNAPLSLANIPGRLQAVFKHK

>SklPDRE

MAKPVPVLLDQLADYFTPIKESSLSFLNATVTSKRVDQDLEKFQNGGCIKDLTAHIKGGELVLTMNYKDSSILEPLSRSSLFKVSQGSVLFKDYDYEDFAKHCPHQVIYNNEKDVHIPHLTVEQTVDFAISCKFNYKPFVNHSIRDVLLKTFGLERARHTIVGDDHVRGISGGERKRLSIIESFIANGSLYLWDNSTKGLDSSTALEFIQCLRSMAKLTRTINIVKISQASDKLVEQFDKILIILENYQVFYGTVSDCIQFFKDLGFRKDPHLCDIEFLTAIINKNLKSPKVSSEEDIYTHWIKSKYYAGVRAQLSVNQNNNHVTNPISKIDPQDLSPLYGVSTYKQLKSCVIRAFQRALGDRTYLTAQLISIIIQSLVIGSLFYSVPKTTIGSFSRGSLTFFALLFFTFNCLADVPISFVRRPVISKQRQMYFYQPWIENLASTIYEFPYKFILVLVFTIILYFLAHFQYSAARFFVFLLFLVTANFVMSVLFQTIAYLSPTVAVANAAGGVLLLAISMYASYVIYLKSMRPWFKWIAYINPVMYAMESMLSNELFNMDLDCTDSIIPRGPTYDNVSFSHKVCGWQGAKLGHSSVKGREYLNDALGYSYQHVWRNFGILIGFSVFFTVFSLLASQYITPRYDGKTFKLFKKKNYPKRSSKAHDDVPLTDVEFFRFESVMDKAEKNTETDNSLCTEQAVLKSCDTNVVSWKNVNYTVNGKHLLKDVSGYISSGLTALMGESGAGKTTLLNVISQRIEKGVVNGELLVNGCAVKDKNAFKRSVGYVQQQDVHINLLTVWESLEISCRLRGDGDLLYAEKVLDMLKLPREKLFKDLNPAEKKLLSIGVELVTKPSLLLFLDEPTSGLDSEAAVTIIKFLKKLTAQGQAVFCTIHQPSKNIFSYFDNVLLLRKGGECIYFGPRDQIYDYVKRHYSISYDKETENPAELVLDAIGAGANSDSNIGWLSIWNSSKEKKDITDIISQLEDEALADGTNYIELLHETPSYLTQLFIVTKRQYLSITRDRSYMTSKILLNVVAGLFIGFTFWKTKHNIIGLQNLIFTVFMALCISDPLINQLQAKAFEPKEVFITRESKSNTYHWSVLLLSQLLVEIPLVLIGSTFLYLCFYFCLGVDNSPHIAGVFYLNYMLFAIYYLTFGLWLMYMCTDLQTSAVFVAFLFSFTVSFCGVMQPYALFPNFWKFMYRVSPYTYFVDTFVSLLLHERKVICDTSEFAPSQPLAGQTCGQFMKDFIDEHGGYLLNPNAPFVCAYCSYTSGDEFLSIQNMGYHNRWRNFGICCAFVAFNLFAMFSGFYITSVKKLWSVLFEKFSMVLLKKKRFGQKHG

>ScAUS1

MSISKYFTPVADGSLTFNGANIQFGADAQGESKKSYDAEDSMPNPANQLNDITFQAEAGEMVLVLGYPTSTLFKTLFHGKTSLSYSPPGSIKFKNNEFKSFSEKCPHQIIYNNEQDVHFPFLTVEQTIDFALSCKFDIPKGERDQIRNELLREFGLSHVLKTIVGNDFFRGVSGGERKRISIIETFIANGSVYLWDNSTKGLDSATALDFLEILRKMAKATRSVNLVRISQASDKIVDKFDKILMLSDSYQLFYGTVDECLTYFRDTLGIEKDPNDCIIEYLTSILNFQFKNKNLGNLSNSSSASVLKTATGEVTKYTYNSDFDLYDQWKHSSYYRNIKQQIQGSSIDDSIKEVDPSDVSPIFNIPLKKQLLFCTKRAFQRSLGDKAYMTAQFISVVIQSLVIGSLFYEIPLTTIGSYSRGSLTFFSILFFTFLSLADMPIAFQRQPVVKKQSQLHFYTNWVETLSTTVFDYCFKLCLVIVFSIILYFLAHLQYKAARFFIFLLFLSFYNFCMVSLFALTTLVAPTISVANLFAGILLLAIAMYASYVIYLKNMHPWFVWIAYLNPAMYAMEAILSNELYNLKLDCSETIVPRGPTYNDVPFSHKACAWQGATLGNDYVRGRDYLKQGLSYTYHHVWRNFGIIIGFLVFFIACTLFASQYIKPYFNKDEIERNNSRLTRWLPFLNKKRGTRSSARNDSKYVGIPKSHSVSSSSSSLSAVPYQISPSNKEMALNDYNEQPITETVETQKHIISWKNINYTVGTKKLINNASGFISSGLTALMGESGAGKTTLLNVLSQRVETGVVSGEILIDGHPLTDEDAFKRSIGFVQQQDLHLDLLSVKESLEISCLLRGDGDRAYLDTVSNLLKLPSDILVADLNPTQRKLLSIGVELVTKPSLLLFLDEPTSGLDAEAALTIVKFLKQLSLQGQAIFCTIHQPSKSVISHFDNIFLLKRGGECVFFGPMDDACGYFMSHDNTLVYDKEHDNPADFVIDAVGNSNSSAGKDTAEEALTLNKEAIDWSALWESSVEKKLVKKETARLEDDARASGVDYTTSLWKQPSYLQQLALITRRQYICTKRDMTYVMAKYCLNGGAGLFIGFSFWHIKHNIIGLQDSIFFCFMALCVSSPLINQIQDKALKTKEVYVAREARSNTYHWTVLLLSQSIIELPLALTSSTLFFVCAFFSCGFNNAGWSAGVFFLNYMLFAAYYSTLGLWLIYTAPNLQTAAVFVAFIYSFTASFCGVMQPYSLFPTFWKFMYRVSPYTYFVETFVSILLHNWEIKCDMSEMVPGQPLTGQSCGQFMEAFIEEYGGYLHNKNTFTVCAYCTYTVGDDFLKNENMSYDHVWRNFGIEWAFVGFNFFAMFAGYYLTYVARIWPKVFKIITKVIPHRGKKPVQN

>Cne5470

MDDQKLKDAGVGSSRLTTDAASPKQDLSSTEECLGDQLRRSSQSLPSRGSMSSSNRNVVEDLELVPIQHTDIPPVTPSQITNPPVLVESEEQGRLHRENGDWRKSALLNLPPPLPFDLAVEMLTIGVPDRKALRWIPNLSKVFAKRKEKDEEETERPKKKWILQDVSCECQNGEVLAILGGSGSGKTTLLNAIANRISGLPTTNGEVAYYSAGRHAEITRGMKLEKGQVKKRIGFVRQQDFLVECLTAKLRLPTHLSGDAISFIVEQTIDELGLRDAADTVVGGPLRKGISGGEKRRLSIGCVLVTLPSVLVLDEPTSGLDAFTSYLLLLTLSQLARRGRTIILSIHAPRSDAFEIFDRVALLSKGRIVYSGLRKDCLAWFYSLGHEVERGVNPLDFLIDVSSVDNRTPENEEASIARVRSLIQAWNNRSPTHWADNLEKASMQSSTSSISIDHLSPQGNNFDEVSSNGVGGDDTSPDIDARDEQRPGLWKQTRVLTARAHRNVYRNVPQLVGFAMQAVLLGVIIGVTYYQLPETPTGIQSLKNLSFQLIPGVFYLQQVFWIYKFCTDLVIFDREREDRLYDVIPYVFSDFISYLIPSILSPTIYVVLVYFISKLRTDDLAANLFITIASTILVQFTTQGLALLSASLLRSFSAASMLGNSLNLFQMTNVPVYVAWIRWISPYFYSFRIVATTQFKDRIFDCPAESNANLNQCLGNNVLNGLNFDYTINIGAWFGGLIGLAVVEYALACVVLWGLPAGGVKHASEIDSHHRGKETDVRESHMTRDKIDVSVRNLSLIWKRRGRGSLKDREKVILNDLSLDFPAGEVSAILGPSGAGKSTLLQLIAGRQLNPGPFSHYAISGTLLFANQPISRTTQSNIAFVEQDDDWHLPSLTVRETLTYAAILRLPDKMPKKQKVARAETVLRMLGLKDCADLPVGGALLKGISGGEKRRLSLAVQMINDPAILVVDEPTSGLDASIALGVMHVLRDIAATGRTVIATIHQPRSDIWRLTDNVTLLAKGGTIAYTGKRSDAVQYFASINYAMPSEFFNPADHLLDLVSIDPRVSYHDQSLTRVNSLTSQWRIRSAGQENGDEQGKNGAGSAIIKHGEGTTPMRIALPVVLERHWKNLWRRKDVFFNRLGQTPLLGALFILFFQRLTHGPAGAQDRIGVTIESTSAIAFVGLLNAMSIFPADRNLYLHEAKSSARYSPATFVITYSLVEVGFELVGGFGYGAIMNVAIGMQTSIRIYFEYSISIWAMVNMGESFAMIFGSWIQTEGLTVTVVSTILSLIGQVSGVISLSVPAWLAAIAWGTCVKAATRVQIINEAVGLEFHCTNEEISSGACVAQSGEQLLALFNWHDLDTAKFMGIMVAVCIAWRIMGWASLAARVGGIR

>AfuYOL075C

MEKDDDLIAELKAPEDLVFADGLQDQTATFNLSLRAVDPVDVCVENLSLQVDTTRPIWKTSPSQLWNRLCGKTMDTHTHKTVLDSVNAFMPSGSLTAIIGSSGSGKTSLLNIMAGRMSLTKAKVSGATTFNGVAGIEGIRSAYVMQEDVLIPTLTVRETLRYAADLRLPSPATQEERHQVVEQVVLELGLKECADTRIGTNTHKGCSGGEKRRTSIGVQMLANPSVLFCDEPTTGLDATSAFQIIRTLKRLAEDGRTVIVSIHAPRSEIWSLFDNVILLARGSVLYSGSRQDSLSHFETCGHVLPPFVNPAEFLIDLAAIDNRTESLEAASMARVELLKAAWKSRSSERKQIEQSRHKGKMSTPSGAFTTSPNKTASFRQQFRVLTSRTFTTTIRDPLGMAGSLLEAVGMAVINGWIFLQLDESQAGIRSRQGSLYTASSLNGYLILLYETYRLTIDIRLFDRERNEGVVGVPAFLLSRRAARLPLEDLPVPIIFAIIYYFMVGYRLSVAQFFVFLLLTILTHYIAVTFAAVSIGVARSFPGASLVGNLSFTLQSFACGYFVQSNQIPVYVRWLKWVAYTFYIFGALCANEFIGPDGPPEGQFYDCPYSTDPSNPACTQYTGRYIMENLGFPSNWIWRPIVILVAFVIGHYLLAGLLLQYNHFAIDIAQARKTDVDLSAGKEKFAERRSEEARPVAISLDKYALEIRKRQVSRRGSRTLFILKPITAEFQPGKLNVIMGPSGSGKTSLLNSIARRLRGSLGTQYRLQGNMLYNGAVPSESVIRSVTSFVTQDDDALMPSLTVRESLRFAAGLRLPQWMSREEKNQRAEEILLKMGLKECADNLIGSELIKGISGGEKRRVTIAIQILTDPKVLLLDEPTSGLDAFTATSIIEVLEALAAEGRTLIMTIHQSRSDLFQHFSRVLLLARGGYTVYAGEGEKMLPYFRSLGYECPKTTNPADFVLDLITVDLQQEDREALTRERVQKLITSWDGQQQDEGRRPSQIATPAELGSLKRRMLPFRITYPLVLHRAAINFWRQPPLVMARSLQVVGIAIIMALFFAPLKNDYAAVQSRMGFIQEFAALYFVGMLQNIAIYPNERDVFYREEADHCYSAETFILQYTTLEVPFEAISSIIFGVLAAYADNLERSPKMFLISAFNCFCIISCGESVGIMFCTLFSHVGFAVNVTSILLSISTILGGVMSLNVNDVLQAINHLSPIKYSIANLAPYAMRDQHFTCTASQLLANGTCPIQTGQQVLQLYNLDKNAPMNVMALGICTIIYRLVAYAMLKVMREGFWRRLR

>CaCDR6

MKEGASVLSISSENQVGVKVRNLTVSVKSQQQKTTKHTEDQEAQYEQGTSKILNNLSFDIECGQLVAIMGGSGSGKTTLLNTLSQRTNINNKNLGFSGSVTYETSSSNKHIKHAYLLQTDIFLPGLTVWETLSTQADLRLPSHVTKQEKIELIEYILDVLELSHLKNTYVASFSSNASTLSGGEQRRVSLAIQMLSKPAILFLDEPTTGLDTSSSLKLVHVLKKLASPEYGITIILSIHQPRPEIGQLFDKICLLTRGGRLVYFGNLANAEMYFNNLNFLGRDSDDHSKHILEYIMDLSVKDTTSVEKEQQTVERINKLVQTWSNNHQFQQQQEENRSSKESHQFKKNLTLFSKPKTDKISFWQEVNVLTKRTFKLTFRDYKRLLVFNVGIVIIGVTVGWMFYRPKHDLAGIRSLTSTLYVAMEIMGFVPMYFEIERLWETDGVFFYREYSENQVSIPGFLISRRLGKLFLEDLPMSLLFSIITFFMWGLRLGDGSHFGIYFVVVFLIELCCMGTAMFSFAIAPSYPISALLINLIYQIQNSACGYFVNAATMPVYVKWTKYLAYFWYAFGALTNNQFSGWHGKCPYNDINDPRCQEFSGDYQIKILGFPVGWVGAPIGYLVLWTVGFFVLSGILFYFKQHDVSMAKTKKNTIGEGEEDHEALHQKKTEREQDYITDKHDLEININNIHLDVTTSNLFGQTKSTKTLLDNVTASFQANKVNVIMGPSGSGKTTLLNYLSNRLSRNSKFIASGSIRLNGIQKISRDQLSKISAYVTQHDSSLIEQLTVRETLYYQAKLRLPLDQHKFIPTIINKLIRQTGLVDCADTLIGSEYVKGISGGEKRRVSIAIQLLSKPKVLFLDEPTSGLDSSTAETILTLLGELAKENNTTIILTIHQPSEQLFYKFGSLLLLGRGGKVIYDGTSVGIVEYLESLGYNNPEGHNIADYILDLISRGMNEDKMQSERRVAELISYWQANSIKKLCSTATFSQEIIDLPQYYYQRLPIFITFPAIFRRQLLTSYRAKDVVINRAGQTIFLAIVHTLYFTPLRNTQEGISNRLGLVQEVLNLYFAGLINNITLYPFERNLFYQEYRDGIYGVTEFGLSYLINELPTEVIPCFFFAALIVFACGLPRTPQMFFAMFGTGFVSINCGESLGIFVNSIFTHMGVATNVLSTFVSLAIFMGGTMSLHMPGFFKGINFISPMKYAVAICANLGFKNQSFKCNSEAADCLLTTGEDVLSYYNMKQNLGPMVGGLIGCLVIYRVVAILSIYVRVKWF

>ScYOL075C

MSQQENGDVATELIENRLSFSRIPRISLHVRDLSIVASKTNTTLVNTFSMDLPSGSVMAVMGGSGSGKTTLLNVLASKISGGLTHNGSIRYVLEDTGSEPNETEPKRAHLDGQDHPIQKHVIMAYLPQQDVLSPRLTCRETLKFAADLKLNSSERTKKLMVEQLIEELGLKDCADTLVGDNSHRGLSGGEKRRLSIGTQMISNPSIMFLDEPTTGLDAYSAFLVIKTLKKLAKEDGRTFIMSIHQPRSDILFLLDQVCILSKGNVVYCDKMDNTIPYFESIGYHVPQLVNPADYFIDLSSVDSRSDKEEAATQSRLNSLIDHWHDYERTHLQLQAESYISNATEIQIQNMTTRLPFWKQVTVLTRRNFKLNFSDYVTLISTFAEPLIIGTVCGWIYYKPDKSSIGGLRTTTACLYASTILQCYLYLLFDTYRLCEQDIALYDRERAEGSVTPLAFIVARKISLFLSDDFAMTMIFVSITYFMFGLEADARKFFYQFAVVFLCQLSCSGLSMLSVAVSRDFSKASLVGNMTFTVLSMGCGFFVNAKVMPVYVRWIKYIAFTWYSFGTLMSSTFTNSYCTTDNLDECLGNQILEVYGFPRNWITVPAVVLLCWSVGYFVVGAIILYLHKIDITLQNEVKSKQKKIKKKSPTGMKPEIQLLDDVYHQKDLEAEKGKNIHITIKLEDIDLRVIFSAPFSNWKEGNFHHETKEILQSVNAIFKPGMINAIMGPSGSGKSSLLNLISGRLKSSVFAKFDTSGSIMFNDIQVSELMFKNVCSYVSQDDDHLLAALTVKETLKYAAALRLHHLTEAERMERTDNLIRSLGLKHCENNIIGNEFVKGISGGEKRRVTMGVQLLNDPPILLLDEPTSGLDSFTSATILEILEKLCREQGKTIIITIHQPRSELFKRFGNVLLLAKSGRTAFNGSPDEMIAYFTELGYNCPSFTNVADFFLDLISVNTQNEQNEISSRARVEKILSAWKANMDNESLSPTPISEKQQYSQESFFTEYSEFVRKPANLVLAYIVNVKRQFTTTRRSFDSLMARIAQIPGLGVIFALFFAPVKHNYTSISNRLGLAQESTALYFVGMLGNLACYPTERDYFYEEYNDNVYGIAPFFLAYMTLELPLSALASVLYAVFTVLACGLPRTAGNFFATVYCSFIVTCCGEALGIMTNTFFERPGFVVNCISIILSIGTQMSGLMSLGMSRVLKGFNYLNPVGYTSMIIINFAFPGNLKLTCEDGGKNSDGTCEFANGHDVLVSYGLVRNTQKYLGIIVCVAIIYRLIAFFILKAKLEWIKW

>AsflB8NPU1

MESSGEIKEYDASEDSHQDAEIDRLVDDFLQNQTTSVSERLGDIIFKDLSVIGAGAGHQRMHDVPKTLQRLSKLANLATWSSRKPPPCRAILQRLTGTIRQGEMLMVVGRPGSGCTTVLKALANIREEYLAMEGDVWYGSMDAGTAKQARANQVAFVGEDDIHFPTLSVSTTLKFALNTRRSTSDPDRAQHLQQDLQTVLELMGLAQAAHVRIGSDHIRGVSGGQRRRVSLAEALCTRASLFCFDNPTRGLDSSTAIRFLTTMRKYTTRSQCMTAMSLYQASDLAVAMFDKVLVLNDGHVAYYGPATSAKAYFESLGFYCSPKISVSDFLASMSGTPEGRTPREALDRPVPIHPADFETRFRESSLYQQTVSDAATPPQSKTVGKPKASGYALPLYRQVYECTVRHYQIFLTDRAAWIAEAAGTIVQALLLGTLFRNQRDVTQGLYTRGSALFFCVLIMGLQASAEFGNTFVQRPILLKQKSLRFYRPGAYALGQILADIPWKFIFIMYSLPIYWMINFQRTAGHFFTWLVCLYMGLMALSVMFRAIAVFTNSITRAILPVGLLLNVFIIYTGFYITPPGMKVWLFWIRYLDPMYYIFESVALNEIGTSSYQCSSGDIVPRGSAYNETSYQACAVSGSVAGELSLSGRLYLMAEYGFKNTHLWRNVGINAGFFVFFSVVVMIGMERFRNAAEHMSTIFYRRLPSWVSASASRSADIEEPPIVAETKDSKPSSNHDVKTIGRLETTQSVFAWQELSLQLGDDKRLLHEVSGWLQPGKMTALMGMSGAGKTTLLDTLAQRIQIGRLSGGLYLNGQTLPASMGRRTGFVHQNDIHLASSTVREALQLSACLRRPATVSWDEKMDHVEMLIQLLEMEDIAEAIIGVPGAGLNLEQRKRVSIGVELAAKPDIVLFLDEPTSGLDGNSALSIVQLMRRLSDAGQTILCTIHQPSAQMIEQFDNLLLLVPGGKTVYFGPLGSRCQKILDYFARYTRRCEETENPADYLLAVSAEPDKDWFQVCCSHPFLLPLTWRQSPEYGSTQEQLQKMLQVQEIKDSSSSESDRTYAASYLNQLRVVTQRAFTNYWRDSDYVLGKVQLNIWMGLMNGLTFLQLSNDLTGARGRMFSIFVGVITGPVLSLQIEPRFILLRDQFLARENESRVYHWSIFTISALLVEIPFTLLGGLIYWLLWYYMVGYLTISTRAGYAFLMYELYSLFVASLAQLTASLFPTVLAAQVATGFIWLVVNTFNGPLSPPPLTPRGWRWFYNISPLFYFIEGIGTNAMHALQITCRDSELTTFQTPAGETCASYTAEFFGLANSTGYLVDPNATGLCEYCAYADGDEYVKQYDMSYSQRGNNVGIFIGFILFNYTMAVLATYLIFIFKWRKRRSN

>PechB6GX92

MTATKEQSIATATRTLAGDLVADFVTACQSSVRSKDNSIVWDHLEMEGGGQGHALAPSVGSMVERYARRATSVFGHRSEPSRKLLHYFRGHIKAGEMLMVIGRPGSGCTTFLKSLCHMHAEYKSTTGTLLYGGIQANFEEPAAPVETTFCAEEDIHFPSLTVEETLRFAVNSRFSNVISSAEAHKTVVNLARLFGIDHVLATKVGNEQIRGVSGGERRRVSLAEALVTCPDLICYDNPTAGLDSSTALEFVQMLREYANQSHCTIAMSLYQGSDDMVPLFDKVAVINPGHCIYYGNVVAAKSYFEDLGFYCPPTMSITDFLNSMSAEPEARQSRQAADSWSIPQTPEEFVTAFWKSEKGVRLGTQIEEAKNSASAAEKALGRRKESRQTYSIPILAQILLCAYRQYRIFITDYNAWIVEAACMVVQSIILGTVFRNLPHETSSLYQLGSVVFYAILVPGLQSMSEFGNTFAQRPLLLKHKRYRLYHPMSYGYGQILSDVVWKVVVIAYNIPMYFLAGLHRTAGHFFIFFLVAYISHLSLSMFFRFIAVLSPTVERAGLPVGIFLTTLVIYTGWYIPPPQMQVWLKWFRFLNPMYYAFEALMINEVGTTSYECTSSDLVPRGQTYTDVAYQACAIAGSEPGRTVVEGASYLRVYYDFDNSHLWRNVGINAGFFIFFAVLNVAHIVPSSTSSTENDLEVQGFARPTRKLSSPEEVRSRGGHSFAWKDLHLTLRKDGQERTLLQHIDGVSMTYLQILFSSPLYSDMRLNLGCIESGTLTALMGVSGAGKTTLLNVLAERMDIGKLTGTLYLDGSPLPKSFRWRMGYVQQQDIHLPSQTVREALQMTAHLRRPPSLSTDEKNAYVEEVLDMLGMQDISDALIGVPGAGLNLEQRKRVSMGIELAAKPDILLLDEPTSGLDGQSAISLVQLLKKLSRSGQTILCTIHQPAAAVIEAFDNLILLAKGGRVTYQGPLGEHSSTALKYFSQHVEACDPKRNPAEYLLDVVGAGSRSNVTADWAQIWSESSECRVQDAKLHDLKNEPTAEQSRPQLYATPLSHQFAIVLRRTWLWYWREPEYFSAKLWMNVANGLLNGLTFLNIPNSQQGAFDRVYTIFLSFLMGPPLGLSMEPRFTTFRDIFVYRERASRSYHWIVFVMSSIVIELPFTLITALIYWLLWYFPAGLQTDPTHAGYALLCYWLFSIFTVSLGYLIAAWMPNLNASLMANGFFFMFVNTFAGTLTAREKTPSGWSWYFNVSPLYYLAEGLTTNALYGHELGCTPSEATVFHAPANDTCISYAGSFLQSATGYLVNPDATGACDYCRYSIGQEYYQQFGYDNNRKYRDIGIFIGFIAFNFTAVIVGTYVTKIHKWKRKTE

>AsclPeaB1

MDMLRNRPVKGSLDHDPQLSADGTNTPAASDGSDQRSDVIEKTAQLSEPIADSIRRFFEIRKLDGPDGTGVVFENISVEGSGTGAQAAPTISSAARSAFGVLSPLQHRLAGQFSRPILSGFSGTIDAGEMLLVIGKPGSGCTTFLKTLSYMWDEYKDVHGDLTIGGHPIQESMVKRPQDIVFCAESDDHFPTLTVAETLRFAIRARCGPEASATEVDMMVAQLAKLVGLSQVMNTKVGDAYIRGVSGGERRRVSLAEALATCARLICLDNPTHGLDSSTALEFIETMREWTSQSRCVTAMSVYQASDAIMPYFDKVLVINSGRQVFYGRIGDAKAYFERLGFECLPTTTLSDFLNSMSADPEVRRVQDGKQHLVPRTSEEFEAVFHASTFYQDLQRSLETAKVEARTNPRPLVKARAFSLPLHHQIWYCAYRQFRIVTSDYSLWAVEPATIIVQSLVLGTLFRDQKRATQSLFIFASALFYSVLVPALQSMAEFGNGFAQRPLILKQKRYRICRPIAYALGLVTTDVVWKIAAICYNIPLYFLTGFQRTAGNFFTWFCIVYLEHLALSMFFRSVAIFSPNMHRAVLPVGIFFNMYVLYTGLYIPAPQMQVWLGWLRYLNPLYYAFESVMVNEFRDLSYQCSPSDLVPSGLGYTDMANQVCAVLGSRSGEESLSGMSYLEAQYGFGRSHLWRNVGINAAFFVFFALCSGIGMERLKTPAGRLATVFYKGRPSIRNSQADSESGAVHDDVPPDVSRQLSGDQHHLNANSERDKNHTLAWTGLCLDIETKDGTRRLLDNLNGWVKSGQLKALMGVSGAGKTTLLNTLAGRSSIGTLTGTLALNGQLLPKFFRSRMGYVQQQDIHLPTQSVREALQMTARLRRDESIPLEEKNAYVEKVIEWLDMEDIAEALVGVPGAGLNLEQRKRVSIGVEMASKPEILFLDEPTSGLDGQSAFSIVRLLRRLADSGQAIVCTIHQPAAELVEQFDELYLLSRGGKLVYDGPLGTHCDKAIEYFEQHSRACGQGENPAEYFLDAIGAGSRKEVQADWVGLWQQSQQSKDRERAEKALVPAEGQAPLAPARRSLYAVPFHVQLWVVVQRTWLYYWREPDYAMSKLWMSVGNALLNSLTYLQSPNTQRGAYNRVFSAFMSLIVGPPLGLQVQPRFVTLRDIFVHREREGFTYHWLAFVFAGIIVELPYTFLTSLVYWLLWYFPVGYFRTAPRAGYSFLMYELFAVFATSLAQMCASLMPNIEAAFAANGFFFMFCNTFAGTLSPKPVTPSGWRWYYKVSPLFYLGEGVTVDVLQDLPLRCAESEVSIFQPPNGTTCGQYAANFLQQATGFLLNTDSLSDCQYCRYRDGQSYYQQYGYDFANRYPNIGIFIGFIAFNFTMVLVMTYLTKIRR

>MlusPDR1

MTSTHHLQGSSGEKGREYDQHSSDGSTQTVYQNGLHEFENHGDAFGEPTANAVNIDAAKDEYVDLKRELSRISRLSTHASKLEEGAAEADEFNLDEFLHDLRKNQNENGHELKNLGLIWKNLTVKGQAADAHTIPTVFTFLQFWKFFGVGVSKNKKVILNDLTGHCKPGDMLLVLGRPGAGCTSFLKVMANMRGAYTDVEGTVSYGGIDAETFAKRYRGQVCYNEEEDQHYPTLTAKQTLQFALRMKTPGKRLPDESKTDFVNKVLYMLGNMLGLTKQMNTMVGNAWVRGLSGGERKRMSIAEQMTTSSSINCWDCSTRGLDAASALDYVRSLRIMTDVFKKTTVATLYQASNNIFTLFDKVLLLDEGRCLYFGPTAGAKEYFESLGFVCPKRKSIPDFLTGLCNPNEREVIPGWEDVAPKFASDFEQKYLASDVYKQMMAEFAEYEQTVQNENPADVFKKAVDEEHQKRAPKKAPFTASFYQQVKALTIRQYYLNITDLGALISRYGTILIQSLITASCFFKMTQDGTGAFARGGALFFAVLFNSFISQSELMGFLMGRPILEKHKQYALYRPSAFYIAQVVMDIPYAIVQVLLFEICAYFMMGLKLTAGAFFSFFIILFFMNLCMNGFFRFFGASTTSFFLATQVSGVILIAVTNYTGYTIPYNKMHPWLSWIYWINPMTYCYKALLINELKGVEYSCEGAGNAVPYGPGYDDWNYKVCTMAGGKPGNDYVLGDDYLNDYLSYNPSQMWAPDFIVIIAFFLLFTVMTAGAMELVGLSKSGTLTKLYLPGKAPKPRTAEEEDARRRKQADFGKNMESVSAGTTFSWQNIDYSVPIKGGSLQLLNHVSGVVRPGNLTALMGSSGAGKTTLLDVLARRKTIGKVEGRVYLNNEALMTDFERITGYCEQMDVHQPAVTVREALRFSAYLRQPADVPKEEKDAYVETILELLEMEDIGDAQIGVVEFGFGISVEERKRLTIGMELVGKPKLLFLDEPTSGLDAQSSYNIIRFIRKLADSGWPVLCTIHQPSAILFEHFDHLLLLVRGGRTAYHGEIGKDSQIMIDYFESNGGPKCSPDANPAEYILECVGAGTAGKAKADWATIWENSPEAKALTEELEEIHNNSNPNPTRDAKTYATGLGTQFKLVFSRMSLAYWRSPDYNIGRFLNVMLTSLVTGFTFWKLGSSSSDMLNKVFALFGTFIMAMTLIILAQPKFITERQYFRREYASRYYSWMPWAISAVLVELPYIFFYSACFMFGFYWTAGMNPSAESGGYFYIMFSMLVCWAVTLGFVIAAVSESPLMASVINPLVISILILFCGLMQAPSAMPKFWSSWMYWLDPFHYYIEGLAVNELGDLKVQCNEGDLLRFNAPPGQTCGEYTANFFSYGAPGYIANPNATSPEMCGYCTFNSGPEFYESRFDWSASHKWRNFGILCGYFAFNVILFIGLVYVFRKPRR

>MlusPDR2

MDMPSAIEGIRANYPNRDDRYDQRQQLRNENYPTSNLNTNEPAHHPDDDSISYDLERSETRRTEGGYGETEGNAVDIDGAIERYQSIRREFTAQSRRKSSIAAASAPAAAAADDVEKGEQQDFDLTEYLTEQHSQIMAAGLKPKNMGVIWKNLTVQGLGADAKVISTNWTWFTSFIQFWKWGKHQGTDFTILKDNNGFCKAGEMLLVLGRPGAGCTSLLRVLANMRASYTNIEGEVTYGGIDAEEFGKHFRGEVCYNEEEDLHYPTLTTQQTLRFALKNKTPGKRLPGESKAEFIDKLLYMLGNMLGLTKQMNTMVGNAFVRGLSGGERKRLSIAEQMTTRSSINCWDCATRGLDASSALDYVRSLRIMTDILGKTTISTLYQASDSIFHLFDKVMVLDEGRCIYFGPTSTAKQFFIDMGFYCPDRKSTPDFLTGLCNMNEREYREGFEGKVPINAVQFEKVYQESPLFAKMMQERDEYEQKINQDRPAETFREAFSDAHQKHAPKHSPFVATYLEQVKALTVRQFQLILGDKGALVSRYGGVVVKGLIMASVFYMMPQDASGAFSRGGAFLFSLLFNALIAQSELAAFMQGRRVLEKHKHFALYHPSAFYIATVIADIPLALIQVIIFELCVYFMMGLQLEAGRFFTFFIVLVMTNLCMNGFFRFWGAVSPNFFTASQLSSILLIAALIYCGYQIPYTQMHPWLFWIYWINPLAYGYKALISDEMRNLHFSCEGANSVPYGPTYTDQQYKTCILPGAAPGASYILGDDYLAVNYGYYVWQRWINFVAVVLFFFFFTILTALAMEYVDLQKEGSITKVYKKGCAPEADSDEKLMQQVTTVNDQEMEAVTDGTTFSWYDLHYCVPVKGGTRELLNGVGGIVKPGHLCALMGSSGAGKTTLLDVLAKRKTIGKVEGNIYLNGEALAADFERITGYCEQMDVHNPNATVREALRFSAYLRQPQEVSKEEKDEYVEQILGLMEMQKIGDALIGDLEAGIGISVEERKRLTIATELVGKPKLLFLDEPTSGLDAQSSYNIVRFIRKLADAGWPVLCTIHQPSATLFEHFDHLLLLMRGGRTAYFGEIGKDSRTMIDYFESNGGPKCSPQANPAEYILECVGAGTAGKSTQDWADVWAGSSQAKALEQELEHIHSTVDHSASRKVNTYALPFWEQLKLVYMRMNVSWWRCPTYNMGRLFNVCFIGLISGFSFWKLGSSPADMQNRMFSVFTTLLMSNALIILAQPRFMQERMWFRREYASKYYGWAPFALSCVLVEIPYLIVLGTIFLFCFYWTAGLQNESDRIGFFYIHFIMFLFYSVSLGFTIASFSATPPMAAVINPFFTSILILFAGIMQPPSAMPHFWSAWMYWLDPYHYLIEGLVVNVMDGVEVVCGEGDYLILNAPPGQTCGEYMQEFFNNGGPGYLGNPDATGSCNYCQYKTGNDFYEERIGWHYSHRWRNFAILCAYTVFNVMLFMFFVFLFRKQKR

>MlusPDR3

MIDPIARTYSQGGFGEANGNAVDIQSAFEEYEHVRRTLTERSNAAAATGQKVDIEKGEQVFDLTEYLANQHAQLTEAGLKAKNMGLVWKGLTVKGLGADARSILTNGSVILKMLQFWTWGKQAGSEVTILHDNDGFCKPGEMLIVLGRPNAGTSTLLRVLSNMRAAYTSVQGDVTYGGIDAQEFGKYFKGEVCYNEEEDLHYPTLTTEETLRFALKTKTPATRLPGESKTDFIENLLYMLGNMLGLTKQMKTMVGDAFIRGLSGGERKRLSIAEQMTTHSSINCWDGSTRGLDASSALDYVRSLRIMTDIMHKTTVATLYQASDSIFSLFDKVMVLDEGRCIYFGPIASAKAYFVEMGFYCPERKSTPDFLTGLCNLNEREVRPGFEDRVPLNAAQFEKVYKESSLYAQMMRERDAYEAEISKDEPQVSFREAFQQAHNTPFVVSYYQQVRALTVRQLQLIWGDKTSLIVRYVDIIAKGLITASIFYLMPLTVEGLFSRAGAYIFILFFNAVVAQAELPAFMNARGVLEKHKHFAMYRPSAFYVAQVIADFPLAVLQAILFELCCYFIMGFLLDAGRFFSFFINLVAITMCMNSFFRFWGAICPNFYTASQISSIIFTAFIIYVGYQLPYPYMHPWLMWIYWINPLAYSFKALIGTELTGARFSCDGVNGVPFGPTYTEQAYRSCVLPGAEPGASFVLGDSYLAQYYGYYTSQNWINFLAVVLLFVFFSILAALAMEFIDLKKGSTITKVYKKGFTPKVTTTTDHEKQLEQTESRAHQELEAVSDGTTFTWHALEYTVPIKKGKLKLLNNVGGYVKPGNLTALMGSSGAGKTTLLDVLSRRKTIGTIEGNIYMNGEHLANDFERLTGYCEQMDVHNPNATVREALQFSAYLRQPAHVPKQEKDAYVEQILDLLEMQNIGDALVGDLSEGTGISVEERKRLTIAVELVGKPKLLFLDEPTSGLDAQSSYNIIRFIRKLADAGWPVLCTIHQPSATLFEHFDHLLLLVRGGQTAYFGEIGKDSRTMIDYFESNGGPVCAPEANPAEYILECVGAGTAGKASKDWAQVWSDSPEAATLEKELEAIHSTIDHTVTRQVQTYALPFWQQLALVFGRMNTSWWRSPSYNLGRALTVCLIGFITGFTFWKVGSSPVDLQNRMFGLLTSLFMGNSMIILIQPRFIQERTWFRREYASKYYSTAPFALSCILVEIPYLIAVSALFMFFYYWTTGLQNESDRVGYFFIIFVVYLFHSVSFGYVIAAFCESPTLAAIINPFFLTVLLLFNGIFQPPSSMPAFWSSWMYWLNPYHYLVEGLVTNGLESVQVICEDKDYIKINAPPGSTCGEYMSTYFADGGLGYLGNPNSTDVCNYCQYSTGNEYYEHRIGWSFSNRWRNYGLLWLFTLFNVAVFFAFVYAFRKQKR

>MlusPDR4

MAYQNRISNDSTSRSFSSSSNEAATVVDAAAYPQLSQSQSRGGYGESEGNAVDIEVALQEYRSVQRELTRQSKRTQADADVEKAQDNDFDLTEYLANQHEQLTSAGLKSKNMGVIWKNLTVQGLGADARSIATNWSVLAKAAQFWEWAKHKGTDFTILHDNNGFCKSGEMLLVLGRPGAGCSTLLRLLANMRGSYTSIEGDVSYGGIDAHDFGKHFKGEVCYNEEEDLHYPTLTTEQTLRFALKNKTPSTRVPGESKQDFINSVLYLLGNMLGLTKQMKTMVGNAFVRGLSGGERKRLSIAEQMTTHSSINCWDCSTRGLDASSALDYVRSLRIMTDIMQKTTISTLYQASDSIFELFNKVMVLDEGRCIYFGPTSEAKAYFTDMGFYCPPRKSAPDFLTGLCNLNERQVQPEYQDRQVPMNASQFEKAYKESAMYSKMMAERDQYEQEINQDKPYESFREAFKQAHDTPAVVSYYDQVKALTVRQFQLIWGDKYSLFVRYGDVIVKGLITASVFYMMPLNGTGAFSRSGAYLFALIFNAFIAQSELPAFMQGRRVLEKHKHFALYHPSAFYFAQVFVDIPLAIIQAIVFELCIYFLMGLASDAGKFFTLLVNLIAITLCMNGFFRFLGAICPNFFLASQLSSFAFVALLINSGYQLPYPDMHPWFMWIYWINPIAYSYKSILSSELRGAHFSCDGVNGVPNGPSYTDPAYRTCVLPGAKPGASFVLGDDYLAQQYEYYTSQIWINFVAVVLFFVLFTVLTALCMEYVDLKQEGTITKVYKKGASPPVVSAETQLKQQQTREERQLEAVSGGTVFSWHSIKYTVPVKSGTRQLLDNVAGIVKPGHLCALMGSSGAGKTTLLDVLAKRKTIGTIEGNIYMNGEHLASDFERLTGYCEQMDVHNPNTTVREALQFSAYLRQSADVPKEEKDAYVEQILDLLEMQKIGDALVGDLEEGTGISVEERKRLTIAVELVGKPKLLFLDEPTSGLDAQSSYNIIRFIRKLADAGWPVLCTIHQPSATLFEHFDHLLLLMRGGRTAYFGEIGKDSRTMIDYFESNGGPTCSPEANPAEYILECVGAGTAGKVTTDWAQVWSNSPEAAALENELQTIHNSIDHTVNRKVEAYAQSFWSQLFFVFKRMNMSWWRSPSYNVGRLFNVVFIGLITGFTFYKVGNTPKDMQNRMFGLLTSMFMGNTMIILIQPRFMQERTWFRREYAGKFYGWVPFALSCILVEIPYLVFLSAVFLVCYYWTTGLQNVSERVGYFFIQFVVYMFHSITFGYAIAAFCESPTMAAILNPFFTTILYLFSGLFQTPKAMPKFWSSWMYWLDPYHYFIEGLVTNGLDSVPVVCNDSNYIKIKAPPGRNCGDYMADFFADGGLGYIGNPNGTDYCDYCQYSIGNDYYETNIGWSYANRWRNCGLLWVFTVFNVVLFVVFVYLFRKQKR

>MlusPDR5

MDMPALQGMPQERHNRDEEYQNRNHYSDESTIEGVHGKEEISSDNETSTANYPNGGAFGEAEGNAVNIEEAMSNYEEIRRELTQQSRISRRKSMTPDQAEKGDVKDFDLTDFLREQTESSESQGFHPKHMGVVWKDLVVQGLGADAKTIPTNWTWIRDSVQFWKWGKHEGHDFTILKGNDGFCKDGEMLLVLGRPGAGCTSLLRVLANMRASYTKIEGSVHYGGIEAREFSKHFRGEVCYNEEEDLHYPTLTCKQTLRFALKNKTPGKRLDGESKGEFINKVLYMLGNMLGLTKQMNTMVGNAFVRGLSGGERKRLSIAEQMTTRSSINCWDCATRGLDASSALDYVRSLRIMTDILHKTTISTLYQASDSIFHLFDKVMVLDEGRCIYFGPTSTAKNYFEEMGFFCPSRKSTPDFLTGLCNMNEREYREGYKNQVPVNAVQFEKAYKESALYSQMMQERDEYEQKINQDRPDEKFRQAFVDAHQKHAPKHSPFVATYFNQVKSLTVRQFELILGDKGALVSRYGGVVVKGLIMASVFYMMPQDASGAFSRGGSFLFSLLFNALIAQAELSAFMQGRRVLEKHKHFALYHPSAFYIATVIADIPLALIQVIVFELCVYFMMGLVLDAGKFFTFFIILVVTNLCMNGFFRFWGAVSPNFFTASQLSSILLIACLVYCGYQIPYNQMHPWLMWIYWINPLAYGYKALISNELHGMHFTCEGPNSVPYGPSYTNTDYQTCNLPGAVPGQTYVLGDDYLHTAYGYETWQRWINFVAVLLFFFFFTILTALAMEYVDLQKEGSVTKVYKAGRAPKEIDESQALEQTVTENDEKMEAVDNGTVFSWHKMNYTVPVKGGKLKLLNDIGGIVKPGHLTALMGSSGAGKTTLLDVLAKRKTIGTIEGRIYLNGEPLGADFERTTGYCEQMDVHNPNATVREALKFSAYLRQPAEVPKEEKDAYVEQIIRLMEMEKIADALVGDLEAGIGISVEERKRLTIATELVGKPKLLFLDEPTSGLDAQSSFNIVRFIRKLADAGWPVLCTIHQPSATLFEHFDHLVLLVRGGKTAYCGEIGPNSRTMIEYFESNGGPKCSPQANPAEYILECVGAGTAGKATKDWSEVWSGSPQAKALDEELEAIHQGITPGLKNHSTPYSLTFAQQFWLVYKRMNVSWWRCPTYNMGRLFNVCFIGLISGFSFWKLGATPSDLQNRMFSVFTTLLMSNALIILAQPRFMQERMWFRREYASKYYGWAPFALSCILVEIPYLIVFSAIFLFCFYWTAGLQNVSDRVGFFYIHFTVFLFYSVSLGFMIAAFSATPPMAAVINPFFTSILILFAGIMQPPASMPYFWRAWMYWLDPYHYVIEGLVVNVMDSVEVVCGDNDWTPINSPPGMNCGDYMADFFNAGGNGYLQSNSSTGVCNYCPYTKGNQFYEERIGWHFDNRWRDFGILCAYCVFNIFAFMFFVFLFRKAKR

>MlusPDR6

MEEKPTEHIEVPHHHPNYNYASSTEDTIAHDDNFGRNGTYGETDVNQVNIQGAKNEYQTLKRELSHMSRKSTPSKVEEGQADSDDFNLDEFLHGIHREQEENGQKRKHLGVSWKNLHVEGLGADAYTIPTVLSNIMAVLKFWKLFKKKDASTKVIIDDLTGCCRDGEMLLVLGRPGAGCTSFLKVIANMRGAYTYVGGEVNYGGINPKEFASKYRGQVCYNEEEDQHYPTLTTKQTLQFALRTKTPGKRLNEESKKDFVDRVIYLLGNMLGLTKQMNTMVGNAFVRGLSGGERKRLSIAEQMTTRSTINCWDCSTRGLDAASALDYVRSLRIMTDVFNITTIATLYQASNSIFNLFDKVLLLDEGYCIYYGPTSGARDYFDTLGFHRPSRKSMPDFLTGLCNPVEREFKPGFEDSAPKHASEFQAAYLQSEVYQTMLRDFESYNEAVEKENKAANFADAIQEEHQKRAKKSRPYIASFYQQVKALTIRQHHLLIKDREALISRYGTILIQSLITASCFFQLPLTGTGAFSRGGALFFSVLFNSFISQSELVRFLTGRPILEKHKQYALYRPSAFYIAQVIMDIPYALVQVLLFEICAYFMMGLNLTAGRFFTFFVVLFFLNMCMNGFFRFFGAITSSFFLATQITGVLLIAITSYTGYTIPYKKMHPWLFWIYYINPITYAYKALLSNEMSGQVYSCDGIGNAVPYGPGYDDWNYMVCTMQGGVAGENFVRGDSYLLEALSYKPWQLWAPDFVVVVAFFLFFTFITALAMELVGMSKSSTLTKLYLPGKAPKPRTIEEEDQRRLDQQKVTDNMDKMSTGTTFSWQHVNYWVPFSGGPLHLLNDISGIVKPGHLCALMGSSGAGKTTLLDVLARRKTIGKVEGNVFLNGEALMNDFERITGYCEQMDIHQPAVTVREALRFSAYLRQPADVPKEEKDAYVEQIILLLEMDDIGDAQIGDVGSGFGISVEERKRLTIGMELVGKPKLLFLDEPTSGLDAQSSFNIIRFIRKLADAGWPVLCTIHQPSAILFEYFDHLLLLVRGGRTAYYGEIGKDSRTMIGYFESNGGPKCSPEANPAEYILEVVGAGTAGKATKDWAEVWQSSEEAKALTAELDEIERTADKNPTREAKMYATPLSTQFRLVMGRMTLAYWRAPDYNIGRFLNIMFTSLVTGFTFWKLGSSSSDMMNKVFALFSTFIMAMTMIILAQPKFMTERLYFRREYASRYYSWLAFGISAVLVEIPYIIFFAAAFMFGFYWTAGMNNTPESCGYFYITFVVLVCWAVTLGFCIAAVAELPTMAAVINPLFISILILFCGLMQAPSAMPRFWSSWMYWLDPFHYYIEGLAVNELADLQVECSDEDLLKFPPPPGQTCGQYMANFFSNGATGYVANPDAVQPEQCGYCTYKSGPEFYETGMQWSASHKWRNFGILIAFFIFNVFVFILVVYLRRKGRR

>MlusPDR7

MEEKKNALYTEQVEDARLRDQPNYAHSNNSTIDADVSAHNKENCGQNDTFGESEANQVNIEDAKNEYQELRRELSTKSQRLSLSKAEEGASNSEDFDLDQFLHGISKAQDENGHKRKHLGVAWKNLHVEGLGADAFTIPTVFSNILSVVQFWKLFKKNKASTKVIIDDLSGCCKDGEMLLVLGRPGAGCTSFLKVIANMRDSYTYIGGDVSYGGIDPKTFAERFRGQVCYNEEEDQHYPTLTTKQTLQFALRTKTPGKRMPGETKADFVDRVLYMLGNMLGLTKQMNTMVGNAFVRGLSGGERKRLSIAEQMTTSSTINCWDCSTRGLDAASALDYVRSLRIMTDVFDITTIATLYQASNSIFNLFDKVLLLDEGRCIYFGPTSGAKQYFDGLGFHCPPRKSLPDFLTGLCNPLEREFKPGFEDSAPKHAVEFQEKYYQSEVHKTMMRDLEDYEKTIAAENKANAFEDAVHEEHQKRAPKKTPYIASFFQQVKALTIREHHLLIKDREALISRYGTMLIQGLITASCFFMLPFTGSGAFSRCGALFFSAMFNVFISQSELVRFLMGRPILEKHKQYALYRPSAYYIAQVTMDIPYSLAQVLLYNICSYFMMGLNLTAGRFFTSFLILFFLTMCMTGFFRFFGSITSSFFLATQITGVLLIATTTYTGYTIPYDKMHPWLFWIYYINPITYAYKALVSNEMEGQLYSCEGAGNAVPFGPTYQDWNYKVCTMAGGNPGENFVRGDDYLRTALSYNPEDLWAPDFVVVVAFFLFFTVLTALAMEYVKLNKAGSLTKLYLPGKAPKPRTTEEEDERRRQQDKITDNMDKMSTGTTFSWQHVNYRVPIKGGSLQLLNDINGIVKPGHLCALMGSSGAGKTTLLDVLARRKTIGTVEGDIFLNNEALMNDFERITGYCEQMDVHQPAVTVREALRFSAYLRQPAEVSKEEKDEYVEQIIQLLEMTDIGDAQIGQVETGFGISVEERKRLTIGMELVGKPQLLFLDEPTSGLDAQSSFNIIRFIRKLADAGWPVLCTIHQPSAILFEHFDHLLLLVRGGRTAYYGEIGQDSRTMIDYFESNGGPKCSPDANPAEYILEVVGAGTAGKASKDWAEVWQASEECKALGAELDEIMATANKNPTREAHTYATSLTTQFRLVYGRMALAYWRSSDYNIGRFLNLMFTSLITGFTFWKLGESSSDMMYKIFALFGTFIMCFTMIILAQPKFMTERMYFRREYSSKYYSWLPWGVSAILVEIPYVFFFSATFMFGFYWTTGMRNTPAACGYFYITFVILVCWAVTLGFVIAAVAELPTMAALINPLFISLLILFCGLMQSPKAMPKFWSSWMYWLDPFHYYIEGLTVNELEDLKIVCTDDDLLRFSVPEGQTCGQYTADFFANGAPGYIANPDAVQPEQCGYCTYKSGPEFYEGNMGWSAANKWRNFGILFAFFIFNIIVFLGLVYWKRKGRR

>MlusPDR8

MSNQKLELSDATPTAPPTRDVHQDAAKASSVSESQEEGTITDEGRYPNSGDMFVGNGDFGEIERDDINVEAAIDEFHELQKELSHISRRSSGMAPEKLEQGEASENDFNLSNFLHGMSDDRREAGHQLKHLGVIWKDLSVEGLGAEAFTIPTVISGLLKNIQFWKRFGVGATQSKKVILKNLSGFCKDGEMLLLLGRPGSGCTTLLRIISNMRGSFTAIKGTVSYGGFDHDTFAKRFHGQTCYNSEEDQHYPTLTTKQTLQFALRTKTPGTRLPNESKKEFINKILYLLGNMLGLTKQMQTMVGNPFFRGLSGGERKRLSIAEQMTTESTINCWDCSTRGLDAASALDYVRSLRIMTDVFKKTTVATLYQASNSIYNLFDKVILLDDGYCLYFGPVASAKQYFEELGFYCPPRKSTPDFLTGICNPLEREIREGYEDKVPVSGEQFQSVFYASPMYQSMMQELQAYEELIERERPSETFKEAMNQEHQKRASNRSPFIASYYQQVKALTIRQYHLLIKDMPALISRYGTILIQSLITASCFYNLPQDGSSSFSRGGALFFAVLFNALVSQTELVNFLMGRPILEKHKQYALYRPSAFYLAQVVMDIPYAIAQVLLFEICAYFLMGLKLTAGAFFTFFITLFFVNMCMNGFFRMFGAVTSSFFFATQFAGVIFISTITYCGYVIPYPDMHPWLYWIYWISPIAYGYKTLLINEMKGQEYSCEGAGNSVPYGPGYDLWDYKVCTMTGGHPGQNFVLGDDYLITKLQWDTRHLWAPDFVAVVGFFLLFTVLTALAMEFRAVNKIGSLTKLYIPGKAPKARSQEEIADQRRKQANKVETMEQISTGTNFSWQNVNYSVPIKGGEIQLLNSISGVVRPGHLCALMGSSGAGKTTLLDVLAKRKTIGKVDGRLYLNGEALINDFERITGYCEQMDIHQPAVTVREALQFSANLRQPYDTPQHEKDSYVEQIIHLLEMEDIADAQIGQVETGFGISVEERKRLTIGMELVGKPQLLFLDEPTSGLDAQSSFNIIRFIRKLADAGWPVLCTIHQPSAILFEHFDHLLLLVRGGRTAYYGEIGQDARTMIDYFEKNGGPKCSPAANPAEYILEVVGAGTAGKAKQDWAEVWEHSQEAKQLEQELEAVHQNADQNPQRHALTYATPMWNQFWLVHKRMALAYWRSPDYNIGRFTTIMFTSLLTGFTYWKLGSSVSDLQSRLFALFSSFLMANILIILAQPKFMTERLYFRREYASRYYGWIPFTISAILVEIPYILFLAAFFMFGFYWTAGLTNTSEAVGYFYLVLIFFVFWAVTLGFVIASVAENPTMAAVINPLVISMLILFAGFMQPEKSMPRFWSSWMYWLNPFHYFIEGLATNELSHINVRCTDKDLLKFLPPPNQTCRDYTNNFFSYGAPGYIDNPDAVQPELCGYCSFTSGEEFYSTTFGWSASHKWRNLGIIIAFFVFNVFCFGALVYWKRKGRR

>AfuabcI

MDEKPAVSESSNGSDVDSLSTASAYEQHRERLRDANPQGVTSHRSGVNVKEAEEEFSELNRQFSTISHQAHCLSKQISRASKPTGKTEDVERSDSPADSDEPWDLETALRGNRDAETAAGIRSKRIGVIWDNLTVRGMGGVKTYIKTFPDAIIDFFNVPETIMHMLGYGKKGKEFEILRNFRGVLQPGEMVLVLGRPGSGCTTFLKTITNQRFGYTSIDGDVLYGIFDADTFAKRFRGEAVYNQEDDVHQPTLTVKQTLGFALDTKTPGKRPLGVSKAEFREKVINMLLKMFNIEHTANTVIGNQFIRGVSGGERRRVSIAEMMITSATVLAWDNSTRGLDASTALDFAKSLRIMTNIYKTTTFVSLYQASENIYKQFDKVLVIDSGRQVFFGPASEARSYFESLGFKERPRQTTPDYLTGCTDPFEREFKEGRSEDDVPSTPDSLVEAFNRSSYSERLAQEMDAYRKKLEQEKHVYEDFEIANQEAKRKFTPKSSVYSIPFHLQIWALMQRQFLIKWQDRFAQTVSWITSTGVAIILGTVWLRLPKTSAGAFTRGGLLFISLLFNGFQAFSELVSTMMGRSIVNKHRQFTFYRPSALWIAQILVDTTFAIARILVFSIIVYFMCGLVLDAGAFFTFILIIVLGYLCMTCFFRVIGCMSPDFDYAMKFASVVITLFVLTSGYLIQWSSEQEWLRWLYYINPFGLGFAALMVNEFKDLTMTCTADSLVPSGPGYDDMASRVCTLAGGEPGSVIIPGASYLAKTFSYFPGDLWRNFGIMVALTVGFLTLNLYHGETLQFGAGGRTVTFYQKENKERRALNGALMEKRTNRESKDQSAANLKITSKSVFTWEDVCYDVPVPSGTRRLLQSVYGYVQPGKLTALMGASGAGKTTLLDVLASRKNIGVISGNILVDGAPPPGSFLRTVSYAEQLDIHEPMQTVREALRFSADLRQPYETPQSEKYEYVEGIIQLLELEDLADAIIGTPETGLSVEERKRVTIGVELAAKPELLLFLDEPTSGLDSQSAFNIIRFLRKLAAAGQAILCTIHQPNSALFENFDRLLLLQRGGECVYFGDIGEDSHVLLDYFRRNGADCPPDANPAEWMLDAIGAGQTRRIGDRDWGEIWRTSSEFEQVKREIIQIKAQRAEEVRQSGGSQIIVREYATPLWHQIKVVCKRTNIVFWRSRNYGFTRLFNHVVIALVTGLAFLNLDDSRASLQYRIFVIFNVTVLPAIILQQVEPRFEFSRLVFFRESACKSYSQFAFALSMVIAELPYSILCAVCFFLPLYYIPGFQAAPSRAGYQFLMVLITELFSVTLGQMISALTPNSFIASQINPPIVIIFSLFCGVAIPRPQMPGFWRAWLYQLDPFTRLISGMVTTELHGRTVSCSPSEFNRFQAPENQTCGEYMLPFFERGGLGYLADNTTQACEYCAYKIGDEFYSAFSMSFNTRWRDLGIFLAFIGSNLIILFLAVSFMSPRCRLRYKLIYDIF

>CneAFR1

MSAAGVPAELNNLGAPITATTQNPSGLANSQVTSGPVSSATQHDEHRSSAGNTLADEEDDKAVEAEKAEAIDAAGDGKQKRLPADSSEDIVAELEPHHVSVHRGKEEFAALERKYSNLSQRSQHELHRPTTRHSVRSSFSRKDRVVSRLTQDDAEKAKEGEGEFNLVEVLRSGRENQDEAGIKRKAVGVVWEDHEVIGAGGMRINIRNFSSAIIEQFMMPAIKVLGIFGFNPFAPKPKAILHPSSGLLKPGEMCLVLGRPEAGCTTFLKTITNQRAGYMEINGNVEYAGVGWKEMRKRYAGEVVYNQEDDDHLPTLTVAQTIRFALATKTPKKKIPGVSAKQFQDDMLDLLLSMLNIKHTANTIVGNAFVRGVSGGERKRVSIAEMFCSGATVCSWDNSTRGLDASTALDYAKSLRLLTDIMGQTTFVSLYQAGEGIYDQFDKVLVLNEGHVAYFGPAKEARQYMIGLGYRDLPRQTTADYLSGCTDVNERRFADGRDATNVPATPEEMGQAYRESEICARMTREREEYKHLMAEDATARENFKQAVLEQKHKGVGKKSPYTVSFLQQVFIIFKRQLRLKFQDHFGISTGFATSIIIALIVGSVYFRLPETASGAFTRGGLLFLGLLFNALTSFSELPSQMLGRSVLYRQNEYRFYRPAAFALAAVLADVPYNASVIFLFSIVLYFMGGLYSSGGAFFMFFLFVFLTFMVMSAFFRTLGVATSDYNVAARLASVLISFMVTYTGYMIPVQRMKRWLFWIFYLNPLSYGYEAIFANEFSRISLTCDSSYTIPRNIPEAGITGYPDTLGPNQMCSIFGSTPGDPNVSGSDYMAVGYSYYKAHIWRNFGILLGFFTFFMFLQMLFIEVLEQGAKHFSINVYKKEDKDLKAKNERLAERREAFRAGELEQDLSELKMRPEPFTWEGLSYTVPVPGGHRQLLNDIYGYVKPGSLTALMGASGAGKTTLLDVLASRKNIGVVEGDILMNGRPIGTDFQRGCAYAEQQDTHEWTTTVREALQYSAYLRQPQHVPKQEKDDYVEDIIELLELQELADAMIGFPNYGLSVEARKRVTIGVELAAKPELLLFLDEPTSGLDGQSAYNIVRFLKKLCAAGQKILCTIHQPNALLFQSFDRLLLLQRGGECVYFGDIGPDSKVLIDYLERNGAEVPHDANPAEFMLEAIGAGSRKRIGSDWGEKWRNSPEFAEVKREIQELKAEALAKPIEEKSNRTEYATSFLFQLKTVLHRTNVALWRNADYQWTRLFAHLAIGLIVTLTFLQLDNSVQSLQYRVFAIFFATVLPALILAQIEPQYIMSRMTFNREASSKMYSSTVFALTQLLSEMPYSLGCAVSFFLLLYYGVGFPYASSRAGYFFLMILVTEVYAVTLGQAVAALSPTILIAALFNPFLLVLFSIFCGVTAPPPTLPYFWRKWMWPLDPFTRLISGLVSTVLQDQEVVCKDGEYQVFPAPSGQTCQQWAGAFAEAVGGYINNPDSTGDCQFCQYRSGQAFFVPLEISFSTRWRDFGIFICYVVFNILVLLIAARFLKWQRR

>Mygratr1

MWGYSVDERKLQREDTNGPPTNQWHNAQRTGGTHPTEEVEGEGEGTWGENDVGGFTTRQAMEDYEALRKDLTQLSKTRSRDTQHSLKRTTTGQTAKSGRKSLTSRQATHTSEAAEQDVEAGPQEEVEESKKDDDDEDDFELDRFMREGHFEKRSDGTSDKRVGVVYKDLTVKGIGSTTSFVRTLPDAIIGTFGPDLFKIICRFVPALAKRTGETRTLLNGFTGCVRDGEMMLVLGRPGSGCSTFLKAISNNRETYAEVTGDVSYGGIPADKQKKMYRGEVVYNQEDDVHFATLNVWQTFIFALMNKTKKKETGNIPVIAEALMKMFGIPHTKYTLVGDDFVRGVSGGERKRVSIAETLASKSTVVCWDNSTRGLDASTALDYARSLRVMTDVSNRTTLVTLYQAGEGIYEVMDKVLVIDEGREIYSGPAKEARQYFIDLGYEAPERQTTADFLTAVTDPVERKFRKGYEHKAPKGPEALEKAFRESPNYQKVLEDITDYENYLKETDYNDAREFEDAVQDGKSKRVSNKSSYTVSFQRQVLACVKREAWLLWGDKTTLWTKLFIIISNGLIVGSLFYGESFDTSGAFTRGGALFFSILFLGWLQLTELMKAVSGRAVVKRHEDYAFYRPSAVTIARVVMDLPVILVQVLIFGIIMFFMTNMTISASQFFIYMLFVYITTILLTALYRMFASLSPEIDTAVRFSGIALNLLVIYTGYVIPRPQLLTKYIWFGWIYWINPLSYSFEAVITNEFAGRTMACAPSQLVPQGPGIDPAYQGCALAGADVNAQSVDGSAYLATQFNYSRSNLWRNFGVVIAFIVLYILVTVIATETVSFAGGGGGALIFKKSKKAKKQVKHAKHADEEKGGIAEDSSSSSKKNASLGDAPNEDKEDEALDKLTKSESIFTWKDVEYTVPYMGGERKLLNKVNGYAKPGVMVALMGASGAGKTTLLNTLAQRQSMGVVSGEMFVDGRPLGREFQRNTGFCLQGDLHDGTATIREALEFSAILRQDASVSREEKIAYVDTVIDLLELNDMQDAIISSLGVEQRKRLTIGVELAAKPSLLLFLDEPTSGLDSQSAYSIVRFLKKLASAGQAIVCTIHQPSSVLIQQFDMILALNPGGNTFYFGPVGENGKDVTKYFSDRGVDCPPHKNVAEFILETAAKPHKRKDGKKIDWNQEWVESQQAKDVLEEIDGLKQTRSHVSTSQKNKDDEKEFAASTMLQCTELLRRTFRQYWRDPSYLYGKFFVSVIVGIFNGFTFWQLGNTQQDMQNRMFTAFLIITIPPTIVNAVVPKFYTNMALWQAREYPSRIYGYFAFVTAQVVAEIPPAIIGAVLYWVLWYWPTGLPTDSSTSGYVFFMTLLFFLFQASWGQWITAFSPSFTVISNVLPFFFVMFSLFNGVVRPYASLPVFWRYWMYYVNPSTWWIGGVLAATLDGIPVQCAETETAHFDAPPGQTCASYAGAFAQSAGGYLLNPQDNTNCMYCPLSTGNQYLAQLNINASDKWRDLGIFVVFVFSNWFLVYFFIYTVRVKGWTFGFGPLFGALGKGVELIKKPFKKGEKKEQSEE

>PechB6HGI7

MEGHSEQSHTVSDSQAHAQSSRQQPSSTGETYHHDASSGPTTEQIDQTTEAPIDATLTDSSTASSSVSDEDGRWGEQKAGKAVSRSGAMEDMEEMRRELTRLSLSRTRSATKSIRRRKSQASRRDEEKAQDEEETEDEAADSFDLGEFLTGGHLERRTTAGEPAKKVGVVFKNLTVQGVETGASFVRTLPQAVVGTFGPDLYNIVCRFVPQLRFGKHPPVRDLIHDFNGAVREGEMMLVLGRPGAGCSTFLKAIANDRGAFAGVNGEVSYGGLSAEDQNKHFRGEVNYNPEDDQHFPSLTVWQTLKFSLINKTRKHDRESIPIIIDALLKMFGITHTRNTLVGNEYVRGVSGGERKRVSIAETLATKSSVVCWDNSTRGLDASTALDYAKSLRIMTDVSKRTTFVTLYQAGESIYELMDKVMVIDEGRMLYQGPANEARQYFVDLGFYCPPQSTTADFLTSLCDPNAREFQPGREASTPKTAEELENAFKNSLAHKRILEDVSSYEKRLQDTQQEDTRRFQSTVAQSKSKSVSKKSPYTVSFVRQVMACVQREFWLLWGDRTSLYTKYFIVISNALIVSSLFYGESLDTSGAFSRGGALFFSILFLGWMQLTELMPAVTGRGIVARHKDYAFYRPSAVSIARVIVDFPAILAMVIPFTIVVYFMSGLDVTASKFFIYFLFVYTTTFCITSLYRMFAALSPTIDDAVRFSGIALNLLILFVGYVIPKQNLISDSIWFGWLFYVNPIAYSYEAVLTNEFSDRVMKCNPSQLVPQGPGVDPRYQGCALTGSTLGESSITGSQYLTANFQFTRSHLWRNFGVVIAFTVLYLLVTVIAAEVLSFVGGGGGALVFKKSKRTKKVAAPATNDEEKVANSNDNAALARGQASSDNGASFNRLSSSERCFTWQNVEYTVPYGNGTRKLLNGVNGYAKPGVMIALMGASGAGKTTLLNTLAQRQKMGVVTGDMLVDGHKLGPDFQRGTGFCEQMDLHDNTATIREAFEFSAILRQPRDVSRQEKLDYVDRIIDLLELEDIQDAIIGCLTVEQKKRVTIGVELAAKPSLLLFLDEPTSGLDSQAAFSIVRFLRKLSQAGQAIVCTIHQPSSMIIQQFDMILALNPGGNTFYFGPVGKDGSAVIKYFGDRGVVCPPSKNVAEFILETAAKPNHRNGKLLDWNEEWRNSDQNREMLAEIENIRTERSKVPIEETGSAQYEFAASTLTQTTQLTKRLFTNYWRDPSYYYGKLFVSVIIGIFNGFTFYKLGNDVASMQNRMFSVFLIILIPPIVLNSIVPKFYINRALWEAREYPSRIYGWVAFCTANVVCEIPAAIISGLIYWLLWYYPVGFPTDSSNAGYVFLMSMLFFFFQASWGQWICAFAPSFTVISNVLPFFFVMVNLFNGIIRPYADYPVFWKYWMYYVNPVTWWLRGVLSAVLPDVQIECAPLEATHFNPPPGQTCDAYAGGFVETAKVGYLVNPQATADCQYCPYTDGVQYMANLNVHLEDKWRCFGIFLAFVIINWALVYFFIYTVRVRGWSFGIGSLFGVAGLMVDRVKGLFKGKKSDEA

>AfuabcG

MDTPSSGTIDLEHGEAGLRKRLTLTFRSVSVHVTAPDAALGDTLLSVADPRQFLGFLKGSRPKRTILKDVSGQVKPGEMLLVLGRPGSGCTSLLRVLSNDRESFDEVIGETRYGSMDHVAARRFRQQIMFNNEDDVHFPTLTVNRTMKFALRNKVPRERPDGQGSKEFVQEQRDNILSALGIRHTTKTLVGNEFIRGVSGGERKRVSLAEVIAGQSPIQVWDNPTRGLDSKTAVEFARLLRREADMNQKTMVATMYQAGNGIYNEFDQVLVLADGRVTYYGPRQLAKSYFEDMGFVCPKGANVADFLTSVTVLTERIVRPGMEDKVPSTAEEFEARYRQSDIHQKAMEGFDPPEKLTHEVDELTAAVASEKRKRHLPRSPSVYTTSLWEQIQACTIRQFQIMAGDRLSLIIKVVSAILQALVCGSLFYNLKDDSSSIFLRPGALFFPVLYFLLESMSETTASFMGRPILSRQKRFGFYRPTAFCIANAITDIPVVLVQVSCFCIILYFMAALQMDAGRFFTYWIIVIANTLCFMQMFRAVGALCKRFGNASKITGLLSTIFFVYGGYLIPYEKMHVWFRWIFYLNPGAYAFEALMANEFVGKSLQCVQPDYIPYGSGYPGSESPYRGCSIPGSEGDVILGAAYIRAQYNYSWHHIWRSFGVIIGFWVFFIVLTALGLELLNSQGGSSVLLYKRGSQKTRSEDTTTPVQEAARASHAKQSTFTWHDLDYHVPYQGQKKQLLDKVFGFVKPGNLVALMGCSGAGKTTLLDVLAQRKDSGEIYGSILIDGRPQGISFQRTTGYCEQMDVHEPTATVREALVFSALLRQPAHVPREEKLAYVDHIIDLLELRDISDALIGVPGAGLSIEQRKRVTLGVELVAKPTLLFLDEPTSGLDGQSAYNIIRFLRKLVDGGQAVLCTIHQPSAVLFEAFDSLLLLARGGKMAYFGETGKDSQTVLDYFARHGAPCPPDENPAEHIVEVIQGNTDKPIDWVQVWNESEEKQRALAQLQTLNARGKADADYVEDTADYATSKWFQFTMVTKRLMVQLWRSPDYVWNKVILHVFAALFSGFTFWKIGDGAFDLQLRLFAIFNFIFVAPGCINQMQPFFLHNRDIFEAREKKSKIYHWLAFIGAQTVSEIPYLILCATLYFACWYFTAGFPTTASISGHMYLQMIFYEFLYTSIGQGIAAYAPNEYFAAVMNPVLIGAGLVSFCGVVVPFSQMQPFWRDWLYYLDPFTYLVGGLLGEVLWDVEVRCDPSELVRFRAPLGQTCGEYMAAFLAEKPGYLVDGNATACEFCQYSTGADYARTFNLKERYYSWRDTGITALFCVSSYAMVFLMMKLRSKKTKSARSE

>AfuabcH

MEDQGHLPSEPRALFDRRDDTDSTNTALDETDLSRTPLQDTSHTPHAEDWSLMPDLKKQHDRNVASGFRRRELGVTWKNLSVDVVSADAAINENVLSQFNIPQHIRESRNKAPLRTILHESHGCVKPGEMLLVLGRPGSGCTTLLRMLSNHRLGYKAIRGDVRFGSLTPEEASKYRGQIVMNTEEELFFPTLTVAQTLDFATRLKVPFNLPDGVTSPEAFRQETREFLLKSMGISHTSDTKVGNEYVRGVSGGERKRVSIIECLATRGSVFCWDNSTRGLDASTALEWAKAVRAMTDVFGLSSIVTLYQAGNGIYDLFDKVLVLDEGKQIYYGPMSQARPFMEEQGFVCREGSNVADFLTGVTVPTERKIRPGYENRFPRNADELLAAYEKSPIRAQMAIEYDYPDTESTRERTEEFKLGVLDEKAKRLSKNSPFTVDFLQQVKACIIRQYQIIWTDKATFAIKQISTVIQALVAGSLFYNAPDNSGGLFIKSGALFFSLLYNSLLAMSEVTDSFSGRPVLIKHKYFAFFHPAAFCIAQIAADIPVLLFQISMFAVVVYFMVGLTTSAGAFFSYWIIIFVATMVMTALFRAIGALFSTFDGASKVSGFLISALIMYCGYLEPYHAMHPWFIWIYWINPLAYAFDALLSIEFHNKIIPCVGNNLVPFGPGYDDTTFQSCAGVGGAVRGMTYVTGDQYLASLTYSYSHVWRNFGILWAWWALFVAVTIIATSRWKSAAEAGNSLLIPRETVAKHHAVVRKDEEAQLNEKAGHKGTGTDSEAQSNVDQHLVRNTSVFTWKNLTYTVKTPSGDRVLLDNVYGWVKPGMLGALMGSSGAGKTTLLDVLAQRKTDGTIRGSIMVDGRPLPVSFQRSAGYCEQLDVHEPFATVREALEFSALLRQPRHIPREEKLKYVDVIIDLLELHDLEHTLIGRVGAGLSVEQRKRVTIGVELVSKPSILIFLDEPTSGLDGQSAFNTVRFLRKLADVGQAVLVTIHQPSAQLFAEFDTLLLLAKGGKMVYFGDIGDNAQTVKDYFARYGAPCPANVNPAEHMIDVVSGHLSQGRDWNQVWLESPEHSSASRELDSIISEAASKPPGTVDDGYEFAMPLWEQTKIVTQRMSTSLYRNCDYIMNKIALHIGSALFNGFSFWMIGDSVADMQLKLFTIFNFIFVAPGVINQLQPLFIERRDIYDAREKKSKMYSWVAFVTALIVSEFPYLCVCAVLYFVCWYYTVGFPSDSDKAGAIFFIMLCYEFLYTGIGQFIAAYAPNATFAALTNPLILGTLVSFCGVLVPYAQIQAFWRYWIYWLNPFNYLMGSMLVFSVFDTDVKCKEGEFAVFDTPNGTTCADYLSTYLQGVGSRANLVNPEATSGCRVCQYRYGSDYLYTINLKDYYYGWRDTAIVCIFVLSSYALVYALMKLRTKASKKAE

>PechB6HHZ7

MALSTSSTDSNIEDISNLEKLPDETREIEAQYPTGDVFPGTQATWHMADELQALKERDEQNGEKARKLGVTWQNLTVKGVSSDATFNENVLSQFNLFGNHGSKSPMKTILHNSHGCVKPGEMLLVLGRPGSGCTTLLNMLSNNRRGYAEVSGDIAFGNMSAEEAKQYRGQIIMNSEEEIFFPTLTVGETIDFAARMKVPSQLPPGIKSAEEYAELNKKFLLRSVGISHTESTKVGDAFTRGVSGGERKRVSILECLTTRASVFCWDNPTRGLDASTALEWTKAMRTMTDVFGLTTIVTLYQAGNGIYENFDKVLVLDEGKQIFYGPQRNAVPFMENLGFRRDSGSNRADFLTGVTVPTERIIAPGYESTFPRTSDAIRSAYESSSSKSEIQAECSYAQSKEAAENTAIFKEMVAREKHHGVREKSPVTTDFLSQVKASVTRQYQIMWGDKATLAMKQGATVIQALLGGSLFYNAPDNSIGLFLKGGALFFSILYNALIALSEVTDSFTGRPILAKHRSFALYHPAAICISQIVADFPILLFQVSHFGLVLYFMVGLNRTAEAFFTYWITNFMTAMSMTALFRLIGAAFPTFDAATKVSGLTIVSCFVYTGYMIIKPEMHPWFVWLFWINPMAYGFEALLGNEFHSSIIPCVGPNLIPNGPGYTNGEGGQSCAGVGGASPGATSVTGREYLASMSFSHSHVWRNFGIICAWWVLFVALTIFFTSRWKLPGEGARSLLVPREQQYKSKHLLLGDEESQSMKTLPNSEANTSQETIGKELNGNRSIFTWKNLTYTVKTSSGDRVLLDNVQGYVKPGMLGALMGSSGAGKTTLLDVLAQRKTDGTIHGSVLVDGRPIPISFQRSAGYVEQLDVHESLATVREALEFSALLRQPRDTPIDEKLRYVDTIIDLLELRDLEFTLVGRPGAGLSVEQRKRLTIAVELVAKPSILIFLDEPTSGLDGQAAFNIVRFLRKLAEAGQAVLVTIHQPSAQLFAQFNTLLLLAKGGETVYFGDIGDNASTVKAYFARHGAPCPPEANPAEHMIDVVSGAASENADWNKIWLESPEHDQLTTELDAMATEAAARPSGTVDDGHEFAASMWTQVKLVTHRMNVSLFRNTEYIDNKFALHISLALLNGFSFWMIGDRLTDLQKNLFTVFNFIFVAPGVISQLQPLFIDRRDLYETREKKSKMYHWAPFVAGLIISEIPYLIVCALLYYFCWYFTCGLPTAPGNAGSVFFVVVMYECLYTGIGQMIAAYAPNAVFASLVNPLVITTLVSFCGVMVPYSQIEPFWKYWMYYIDPFNYLMSSLLVFTTWSKPVTCTPDEVALFNPPVNQTCGEYLATYQQGMGVGTNLLNPSANADCQVCQYTTGGDYLKSLNLAEEYFGWRNAGLVALFVLGIYGLVFLMMKLRTKATKKAEN

# References

1. Lax C, Mondo SJ, Osorio-Concepción M *et al.* Symmetric and asymmetric DNA N6-adenine methylation regulates different biological responses in Mucorales. *Nat Commun* 2024; **15**: 6066.

2. Lamping E, Monk BC, Niimi K *et al.* Characterization of three classes of membrane proteins involved in fungal azole resistance by functional hyperexpression in *Saccharomyces cerevisiae*. *Eukaryot Cell* 2007; **6**: 1150-65.

3. Sagatova AA, Keniya MV, Wilson RK *et al.* Structural insights into binding of the antifungal drug fluconazole to *Saccharomyces cerevisiae* lanosterol 14alpha-demethylase. *Antimicrob Agents Chemother* 2015; **59**: 4982-9.

4. James JE, Lamping E, Santhanam J *et al.* PDR transporter *ABC1* is involved in the innate azole resistance of the human fungal pathogen *Fusarium keratoplasticum*. *Front Microbiol* 2021; **12**: 673206.

5. European Committee on Antimicrobial Susceptibility Testing. *The European Committee on Antimicrobial Susceptibility Testing. Routine and extended internal quality control for MIC determination and agar dilution for yeasts, moulds and dermatophytes as recommended by EUCAST. Version 6.0*, 2022.

6. Corrochano LM, Kuo A, Marcet-Houben M *et al.* Expansion of signal transduction pathways in fungi by extensive genome duplication. *Curr Biol* 2016; **26**: 1577-84.

7. Lee SC, Billmyre RB, Li A *et al.* Analysis of a food-borne fungal pathogen outbreak: virulence and genome of a *Mucor circinelloides* isolate from yogurt. *mBio* 2014; **5**: e01390-14.

8. Lebreton A, Corre E, Jany JL *et al.* Comparative genomics applied to *Mucor* species with different lifestyles. *BMC Genom* 2020; **21**: 135.

9. Grigoriev IV, Nikitin R, Haridas S *et al.* MycoCosm portal: gearing up for 1000 fungal genomes. *Nucleic Acids Res* 2014; **42**: D699-704.

10. James JE, Lamping E, Santhanam J *et al.* A 23 bp cyp51A promoter deletion associated with voriconazole resistance in clinical and environmental isolates of *Neocosmospora keratoplastica*. *Front Microbiol* 2020; **11**.

11. Livak KJ, Schmittgen TD. Analysis of relative gene expression data using real-time quantitative PCR and the 2(-Delta Delta C(T)) Method. *Methods* 2001; **25**: 402-8.

12. Boeke JD, LaCroute F, Fink GR. A positive selection for mutants lacking orotidine-5'-phosphate decarboxylase activity in yeast: 5-fluoro-orotic acid resistance. *Mol Gen Genet* 1984; **197**: 345-6.

13. Alani E, Cao L, Kleckner N. A method for gene disruption that allows repeated use of URA3 selection in the construction of multiply disrupted yeast strains. *Genetics* 1987; **116**: 541-5.

14. Wilson RB, Davis D, Enloe BM *et al.* A recyclable *Candida albicans* URA3 cassette for PCR product-directed gene disruptions. *Yeast* 2000; **16**: 65-70.

15. Lamping E, Tanabe K, Niimi M *et al.* Characterization of the *Saccharomyces cerevisiae* sec6-4 mutation and tools to create *S. cerevisiae* strains containing the sec6-4 allele. *Gene* 2005; **361**: 57-66.

16. Laemmli UK. Cleavage of structural proteins during the assembly of the head of bacteriophage T4. *Nature* 1970; **227**: 680-5.

17. Madani G, Lamping E, Lee HJ *et al.* Small-Scale Plasma Membrane Preparation for the Analysis of *Candida albicans* Cdr1-mGFPHis. *J Vis Exp* 2021.

18. Pemberton LF. Preparation of Yeast Cells for Live-Cell Imaging and Indirect Immunofluorescence. In: Smith JS, Burke DJ, eds. *Yeast Genetics: Methods and Protocols*. New York, NY: Springer New York, 2014; 79-90.

19. Gustafsson MG. Surpassing the lateral resolution limit by a factor of two using structured illumination microscopy. *J Microsc* 2000; **198**: 82-7.
